# Exploration of Pyrido[3,4-*d*]pyrimidines as Antagonists of the Human Chemokine Receptor CXCR2

**DOI:** 10.3390/molecules28052099

**Published:** 2023-02-23

**Authors:** Max Van Hoof, Sandra Claes, Katrijn Boon, Tom Van Loy, Dominique Schols, Wim Dehaen, Steven De Jonghe

**Affiliations:** 1 Molecular Design and Synthesis, Department of Chemistry, KU Leuven, Celestijnenlaan 200F, B-3001 Leuven, Belgium; 2 Department of Microbiology, Immunology and Transplantation—Laboratory of Virology and Chemotherapy, KU Leuven—Rega Institute for Medical Research, Herestraat 49, B-3000 Leuven, Belgium

**Keywords:** CXCR2 antagonists, pyrido[3,4-*d*]pyrimidines, SAR study

## Abstract

Upregulated CXCR2 signalling is found in numerous inflammatory, autoimmune and neurodegenerative diseases, as well as in cancer. Consequently, CXCR2 antagonism is a promising therapeutic strategy for treatment of these disorders. We previously identified, via scaffold hopping, a pyrido[3,4-*d*]pyrimidine analogue as a promising CXCR2 antagonist with an IC_50_ value of 0.11 µM in a kinetic fluorescence-based calcium mobilization assay. This study aims at exploring the structure–activity relationship (SAR) and improving the CXCR2 antagonistic potency of this pyrido[3,4-*d*]pyrimidine via systematic structural modifications of the substitution pattern. Almost all new analogues completely lacked the CXCR2 antagonism, the exception being a 6-furanyl-pyrido[3,4-*d*]pyrimidine analogue (compound **17b**) that is endowed with similar antagonistic potency as the original hit.

## 1. Introduction

Upon detection of a pathogen or in response to trauma an acute inflammatory response is mounted by tissue-resident immune cells, such as macrophages, dendritic cells and mast cells. It involves the release of a number of pro-inflammatory chemokines such as CXCL1, CXCL2 and CXCL8, resulting in the chemo-attraction of circulating immune cells [1,2,3]. Neutrophils are among the first to be recruited to the site of inflammation, in order to clear the infection, to release cytotoxic agents and other effector proteins that attract a variety of immune cells [4]. Neutrophils are primarily recruited to the injury site via the CXCL8 and human chemokine receptor CXCR2 axis [5]. Hence, it is clear that both CXCL8 and CXCR2 are essential mediators of the immune defense. For example, it has been demonstrated that CXCL8 or CXCR2 knockout in zebrafish hinders wound healing and reduces neutrophil recruitment [6]. CXCR2 deletion in mice resulted in failure to clear bacteria from the bladder and kidneys due to reduced neutrophil infiltration [7].

CXCR2 upregulation has been linked to a myriad of inflammatory disorders (such as asthma, chronic obstructive pulmonary disease, cystic fibrosis), autoimmune diseases (e.g., rheumatoid arthritis and psoriasis), and even neurodegenerative diseases (such as multiple sclerosis and Alzheimer’s disease) [8]. Hence, CXCR2 antagonism is a promising therapeutic strategy for treating these inflammatory illnesses.

Furthermore, elevated expression and signaling of CXCR2 is correlated with aggressive cancer phenotypes and a poor prognosis in various cancers, such as lung cancer, colorectal cancer, pancreatic cancer, prostate cancer, breast cancer, gastric cancer and squamous cell carcinoma [9]. In addition, the glutamate-leucine-arginine positive (ELR+) CXC chemokines, which all interact with CXCR2, directly function as growth factors for tumor cells and stimulate metastases. For example, in melanoma cells, CXCL1 and CXCL8 promote cell growth and metastases, and CXCR2 inhibition has been shown to counteract these processes [9,10,11,12]. A crucial step in tumor development that enables exponential cell growth is the onset of angiogenesis or the angiogenic switch, preventing the tumor cells from becoming necrotic [13]. CXCR2 is responsible for this angiogenic switch by stimulating the migration, survival and proliferation of microvascular endothelial cells, via its interaction with ELR+ CXC chemokines, which have shown to be potent angiogenic factors [14].

CXCR2 recruits myeloid derived suppressor cells (MDSCs) to the tumor microenvironment [15]. High MDSCs numbers are associated with an increased resistance of the tumor to therapy, including both chemotherapy and immunotherapy [16]. It has been shown that malignant cells surviving chemo- or radiotherapy had greater expression of CXCR2 ligands, and knockdown of CXCR2 improved the response to treatment with paclitaxel and doxorubicin in a mammary tumor model. Additionally, this knockdown inhibited spontaneous lung metastases [16]. Moreover, it has been demonstrated in multiple preclinical studies that combination treatments of a CXCR2 antagonist with an immune checkpoint inhibitor inhibited tumor growth, resulting in improved survival [17,18]. Overall, there are many reports showing that upregulation of CXCR2 signaling in different cancers contributes to tumor immunity and development, angiogenesis, metastasis and drug resistance. Consequently, CXCR2 antagonists are a promising class of drugs in (immuno)oncology.

**Figure 1:**
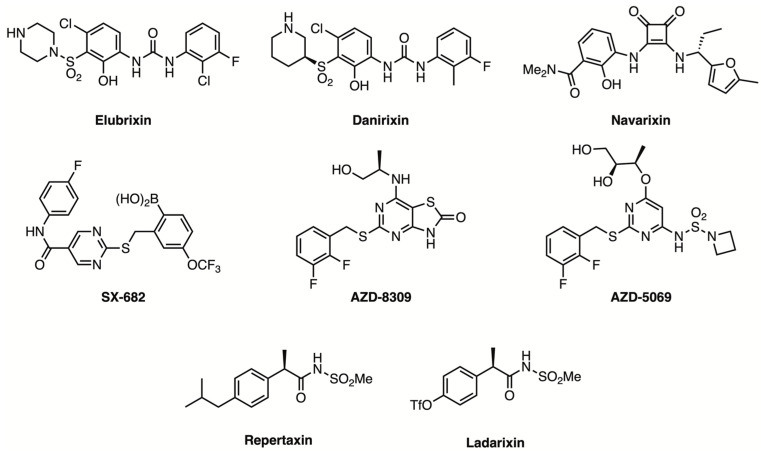
CXCR2 antagonists evaluated in clinical trials.

Because of the crucial role of CXCR2 in inflammation and oncology, various small molecule CXCR2 antagonists have been developed [19]. Several of these are currently being evaluated in clinical trials. The first class of CXCR2 antagonists to be discovered were the diarylurea CXCR1/2 antagonist [20,21]. Two urea compounds entered clinical trials, namely elubrixin [22] and danirixin (Figure 1) [23]. However, both compounds failed to show a therapeutic effect in ulcerative colitis, cystic fibrosis, chronic obstructive pulmonary disease (COPD), treatment of influenza and their effect on neutrophil extracellular traps. Considering the multiple developability issues that urea compounds often suffer from, such as a limited aqueous solubility, poor membrane permeability and poor metabolic and chemical stability [24], several bioisosteric replacement of the urea core have been explored. These include diarylguanines and analogues thereof [25,26,27,28], and diarylsquaramides [29,30,31,32]. Squaramide navarixin has been explored in numerous clinical trials (Figure 1) [30]. It failed in trials against asthma, psoriasis and COPD due to reduced neutrophil counts. Additionally, it was evaluated as a combination therapy with the immune checkpoint inhibitor pembrolizumab in patients with stage III or stage IV castration resistant prostate cancer, positive refractory non-small cell lung cancer and colorectal cancer.

Besides the urea and urea isosteres class, several derivatives with an aromatic ring core have been explored, such as SX-682 [33], AZD-8309 [34], AZD-5069 [35], reparixin and ladarixin [36], which all went into clinical trials (Figure 1). Phase II clinical trials for a combination of SX-682 with nivolumab for the treatment of metastatic colorectal cancer, and a combination of SX-682 with pembrolizumab for metastatic melanoma are ongoing. AZD-8309 was evaluated in a phase I trial for rheumatoid arthritis, COPD and pancreatitis. AZD-8309 was the starting point for the discovery of AZD-5069 via a ring opening strategy [35], which has gone into several clinical trials for COPD, asthma and bronchiectasis treatment, which were all terminated. Additionally, different combination therapies including AZD-5069 have been explored. The combination with MEDI4736 was evaluated for treatment of metastatic pancreatic ductal adenocarcinoma, and with enzalutamide with metastatic castration resistant prostate cancer. Reparixin was evaluated in several phase III clinical trials for preventing graft rejection after organ transplantation, in a phase I trial for treating metastatic breast cancer, and a phase II trial for the combination of reparixin and paclitaxel in treatment of metastatic breast cancer. A phase III trial for ladarixin in the treatment of recent onset type 1 diabetes is ongoing.

**Figure 2:**
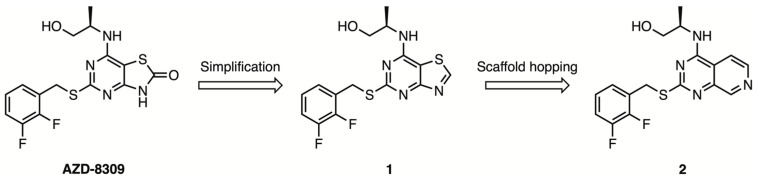
Previous scaffold hopping study leading to pyrido[3,4-*d*]pyrimidine **2**.

Based on the thiazolo[4,5-*d*]pyrimidine core of the known CXCR2 antagonist AZD-8309 [34,37,38], we previously reported on a scaffold hopping study in which the central core was replaced by various other pyrimidine fused bicyclic heterocycles, with the aim of identifying CXCR2 antagonists based on underexplored chemotypes (Figure 2) [39]. The pyrido[3,4-*d*]pyrimidine analogue 2 emerged as a promising hit, displaying an IC_50_ value of 0.11 µM as CXCR2 antagonist, as measured in a kinetic fluorescence-based calcium mobilization assay [39]. Although pyrido[3,4-*d*]pyrimidines with CXCR2 antagonistic properties are not known, this scaffold has been associated with various other bioactivities. Pyrido[3,4-*d*]pyrimidin-4-ones were shown to be potent and selective matrix metalloproteinase-13 inhibitors [40]. 8-Substituted pyrido[3,4-*d*]pyrimidin-4(3H)-ones were potent inhibitors of histone lysine demethylase [41], whereas 2,8-disubstituted pyrido[3,4-*d*]pyrimidines displayed potent inhibition of monopolar spindle kinase 1 [42].

In this study, a systematic exploration of the substitution pattern of the pyrido[3,4-*d*]pyrimidine scaffold is pursued in an effort to explore the structure–activity relationship (SAR) of pyrido[3,4-*d*]pyrimidine 2 and to improve its CXCR2 antagonistic potency.

## 2. Results and Discussion

### 2.1. Chemistry

#### 2.1.1. Synthesis of 5-Substituted Pyrido[3,4-*d*]pyrimidines

**Scheme 1:**
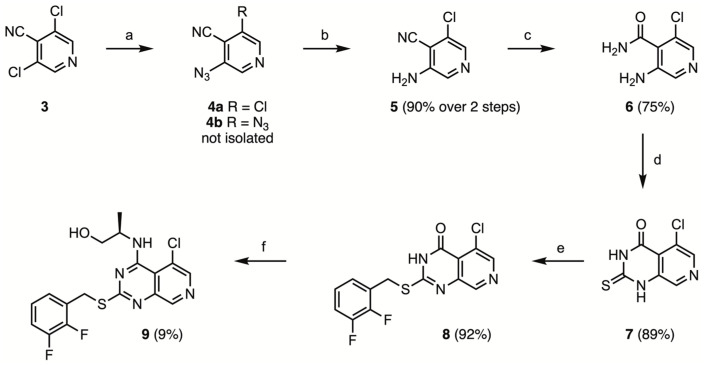
Synthesis of a 5-chloro-pyrido[3,4-*d*]pyrimidine analogue. Reagents and conditions. (a) NaN_3_, DMF, 80 °C; (b) FeCl_3_, NaI, ACN, RT; (c) CsOH (cat.), aqueous ammonia, 100 °C; (d) CS_2_, DBU, DMF, 60 °C; (e) 2,3-difluorobenzyl bromide, Et_3_N, DMF, RT; (f) (i) POCl_3_, 120 °C, (ii) (*R*)-alaninol, Et_3_N, DMF, RT.

The 5-chloropyrido[3,4-*d*]pyrimidine was synthesized starting from commercially available 3,5-dichloropyridine-4-carbonitrile **3** (Scheme 1). A nucleophilic aromatic substitution of **3** with sodium azide gave a mixture of the desired azide analogue **4a**, along with the disubstituted derivative **4b**, in a 9:1 ratio [43]. The reduction of azide **4a** towards aminopyridine **5**, under Staudinger reaction conditions using triphenylphosphine, was unsuccessful since an intractable mixture of products was obtained with the iminophosphorane functionality still intact. Likely, this is the consequence of the chloropyridine being more susceptible to hydrolysis than the iminophosphorane. In a second attempt, the azide **4a** and diazide **4b** were not separated and the mixture was directly reduced using iron trichloride/sodium iodide as reductant in acetonitrile,[44] from which the desired compound **5** was isolated in high yield by flash chromatography. Hydrolysis of nitrile **5** using catalytic cesium hydroxide in aqueous ammonia gave amide **6** in good yield, isolated via simple filtration.[45] The 1,8-diazabicyclo(5.4.0)undec-7-ene (DBU)-mediated cyclocondensation of **6** with carbonyl disulfide afforded the 2-thioxopyrido[3,4-*d*]pyrimidine **7**.[46] Benzylation of compound **7** proceeded in moderate yield affording intermediate **8**. Chlorination with phosphorus oxychloride, followed by nucleophilic aromatic substitution with (*R*)-alaninol gave compound **9** in low yield, because of its low solubility resulting in product losses during purification.

The Suzuki-coupling of 5-chloropyrido[3,4-*d*]pyrimidine **9** with phenylboronic acid gave no conversion when standard reaction conditions were applied, i.e., tetrakis(triphenylphosphine)palladium(0) as catalyst and using potassium carbonate as a base in a mixture of water and dioxane. When transitioning to a more catalytically active system [47], using tris(dibenzylideneacetone)dipalladium(0) (Pd_2_dba_3_), dicyclohexyl-(2′,4′,6′-triisopropylbiphenyl)phosphine (XPhos) and K_2_CO_3_ in *n*BuOH, exclusively hydrolysis of the (*R*)-alaninol moiety was observed, yielding the 4-oxo-pyrido[3,4-*d*]pyrimidine derivative **8**. The poor outcome of these Suzuki reactions can be ascribed to different factors. Lewis-basic heterocycles or compounds having coordinating groups are known to behave poorly in palladium-catalyzed cross-coupling reactions [47]. Compound **9** has a Lewis-basic aminopyridine core and an (*R*)-alaninol coordinating group. In addition, since the coupling partner is a heteroaryl chloride, the rate of oxidative addition is low. Moreover, steric hindrance of the chlorine at position 6 of the pyrido[3,4-*d*]pyrimidine scaffold prevents an efficient Suzuki reaction.

#### 2.1.2. Synthesis of 6-Substituted Pyrido[3,4-*d*]pyrimidines

**Scheme 2:**
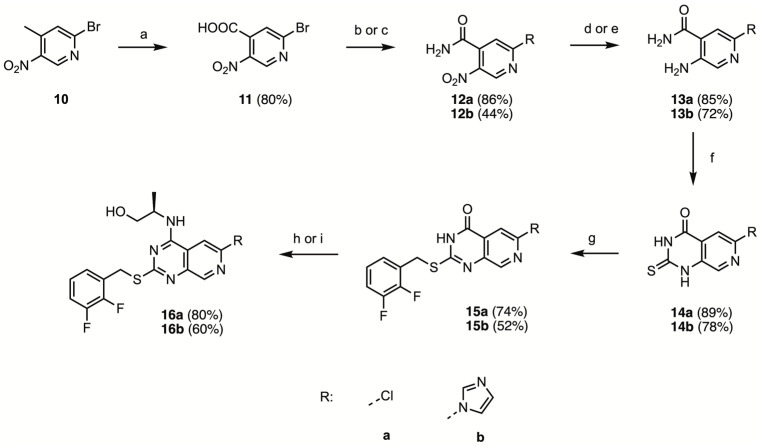
Synthesis of 6-substituted pyrido[3,4-*d*]pyrimidines. Reagents and conditions. (a) K_2_Cr_2_O_7_, H_2_SO_4_, 0 °C—RT; (b) (i) (COCl)_2_, DMF (cat.), DCM, (ii) aqueous ammonia; (c) (i) CDI, THF, RT, (ii) aqueous ammonia, RT; (d) Fe, AcOH, EtOH, H_2_O, RT; (e) H_2_, Pd/C, THF, RT; (f) CS_2_, DBU, DMF, 60 °C; (g) 2,3-difluorobenzyl bromide, Et_3_N, DMF, RT; (h) (i) SOCl_2_, DMF (cat.), 80 °C, (ii) (*R*)-alaninol, Et_3_N, DMF, RT; (i) (i) BOP, DBU, DMF, RT, (ii) (*R*)-alaninol, DMF, RT—60 °C.

The 6-chloropyrido[3,4-*d*]pyrimidine analogue was prepared from commercially available 5-bromo-4-methyl-3-nitropyridine **10** (Scheme 2). The Jones oxidation of the 4-methyl substituent gave carboxylic acid **11** in good yield. Carboxamide **12a** was prepared via treatment of **11** with oxalyl chloride, followed by a Schotten–Bauman reaction with aqueous ammonia. This resulted in a concomitant transhalogenation of the 6-bromo group, yielding the 6-chloropyridine analogue **12a**. Reduction of the nitro group using iron powder under acidic conditions with ultrasonication (Béchamp reaction circumstances) [48], gave the aniline **13a**, which was cyclocondensed with carbon disulfide in the presence of DBU to afford the 6-chloro-2-thioxopyrido[3,4-*d*]pyrimidine-4-one **14a** [46]. Subsequent benzylation gave compound **15a**. Chlorination of the lactam moiety with thionyl chloride and a catalytic amount of DMF yielded the 4-chloro intermediate, which was not isolated, but directly subjected to an amination reaction, furnishing compound **16a** in good yield.

For derivatization at position 6 of the pyrido[3,4-*d*]pyrimidine scaffold via palladium-catalyzed cross-coupling reactions, the 6-bromo analogue is preferred. Therefore, several attempts to prevent this transhalogenation were made. Performing the Béchamp reduction of the nitro group of compound **11** prior to oxalyl chlorination should greatly reduce the chance of transhalogenation, since the aminopyridine is far less electrophilic than the corresponding nitropyridine [49]. However, purification of the 5-bromo-3-aminopyridine-carboxamide proved unsuccessful because of its low solubility. Attempts to prepare the bromo-analogue of **12a** via an intermediate acid bromide using phosphorus tribromide, followed by the dropwise addition of an aqueous ammonia solution while cooling to −78 °C, afforded only trace amounts of the desired primary amide. Alternatively, the carbonyl 1,1-carbonyldiimidazole (CDI) mediated coupling with ammonia (alleviating the need of a chlorine source) resulted in concomitant nucleophilic aromatic substitution of the bromide by imidazole [50], giving the 5-imidazolyl-2-nitropyridine-4-carboxamide **12b** in low yield. Catalytic hydrogenation of the nitro group of compound **12b** furnished aminopyridine **13b**. Cyclocondensation with carbon disulfide towards 6-imidazolyl-2-thioxopyrido[3,4-*d*]pyrimidin-4-one **14b** was followed by benzylation yielding compound **15b**. Next, the one-pot benzotriazol-1-yloxytris(dimethylamino)phosphonium hexafluorophosphate (BOP)-mediated amination with (*R*)-alaninol gave the 4-amino-6-imidazolyl-pyrido[3,4-*d*]pyrimidine **16b** in good yield [51]. In a final attempt to prepare the 6-bromo analogue, a BOP-mediated amidation of the carboxylic acid **11** with ammonia was applied. Unfortunately, this gave a highly complex mixture, from which the desired compound could not be isolated.

**Scheme 3:**
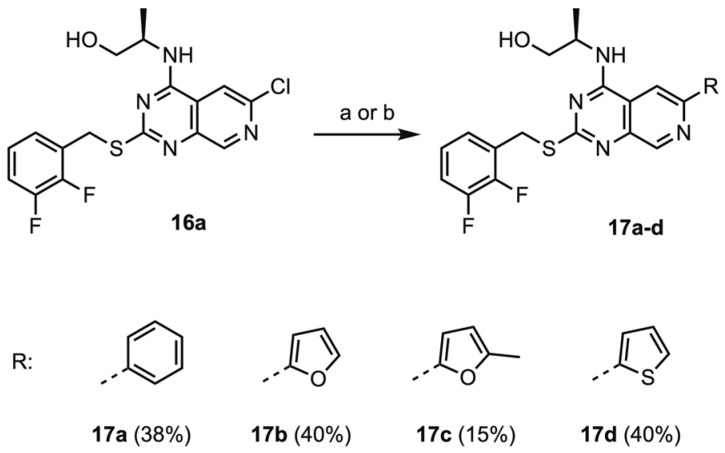
Suzuki couplings of 6-chloropyrido[3,4-*d*]pyrimidine. Reagents and conditions. (a) Pd(PPh_3_)_4_, K_2_CO_3_, 1,4-dioxane/water, 120 °C; (b) Pd_2_(dba)_3_, XPhos, nBuOH, 120 °C.

Since various attempts to synthesize the 6-bromopyrido[3,4-*d*]pyrimidine analogue were unsuccessful, the derivatization of the 6-chloropyrido[3,4-*d*]pyrimidine **16a** via palladium-catalyzed cross-coupling was considered (Scheme 3). The Suzuki coupling with phenylboronic acid, 2-furanylboronic acid and 5-methylfuran-2-boronic acid under the standard conditions gave the desired compounds **17a**–**c** in low to moderate yields. The low yields can partly be explained by challenging purification, since the reactions did not proceed to full conversion. However, for the reaction with 5-methylfuran-2-boronic acid, the low yield resulted from hydrolysis of the (*R*)-alaninol group towards **15a**. For the Suzuki coupling with 2-thienylboronic acid, the more active catalyst system, Pd_2_dba_3_/Xphos [47], was used, giving the desired 6-thienylpyrido[3,4-*d*]pyrimidine **17d** in fair yield.

**Scheme 4:**
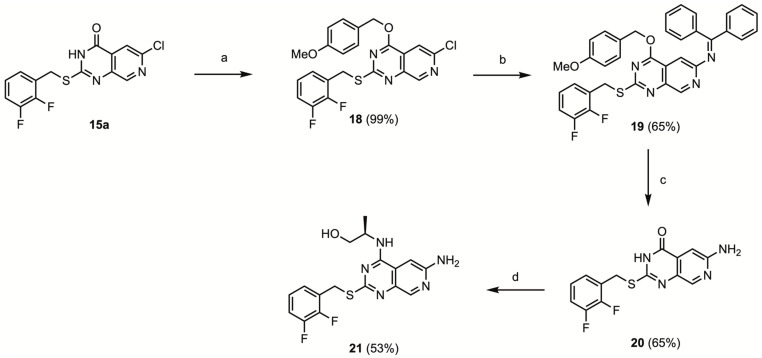
Synthesis of a 6-amino-pyrido[3,4-*d*]pyrimidine analogue. Reagents and conditions. (a) *p*-methoxybenzyl chloride, K_2_CO_3_, DMF, RT—60 °C; (b) benzophenone imine, Pd(OAc)_2_, (±)-BINAP, Cs_2_CO_3_, 1,4-dioxane, 120 °C; (c) TFA, 75 °C; (d) (i) BOP, DBU, DMF, RT, (ii) (*R*)-alaninol, DMF, RT—60 °C.

Finally, the formal introduction of ammonia at position 6 of the pyrido[3,4-*d*]pyrimidine scaffold was performed using benzophenone imine as ammonia surrogate (Scheme 4) [52]. The pyrimidin-4-one **15a** was protected with *p*-methoxybenzyl chloride which proceeded quantitatively to compound **18**.[53] Buchwald–Hartwig amination with benzophenone imine gave the secondary imine **19** in fair yield. Both the benzophenone and *p*-methoxybenzyl protecting moieties were simultaneously cleaved off by heating in neat trifluoroacetic acid (TFA), giving 6-aminopyrido[3,4-*d*]pyrimidine-4-one **20** which was isolated by simple filtration. Finally, compound **21** was obtained via a BOP-mediated amination with (*R*)-alaninol.

#### 2.1.3. Synthesis of 2,4-Disubstituted Pyrido[3,4-*d*]pyrimidines

**Scheme 5:**
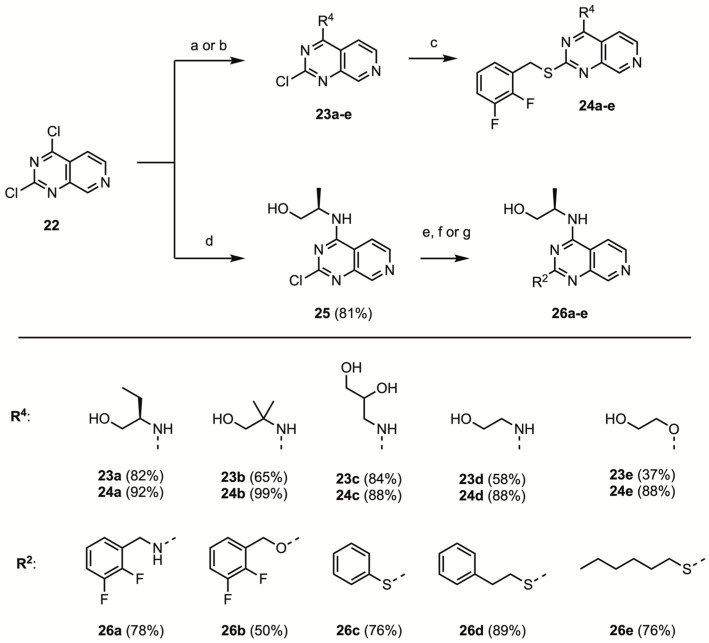
Synthesis of 2,4-disubstituted pyrido[3,4-*d*]pyrimidines. Reagents and conditions. (a) amine, Et_3_N, ACN, 0 °C—RT; (b) HO(CH_2_)_2_OH, NaH, ACN, 0 °C—RT; (c) 2,3-difluorobenzyl thiol, NaH, 1,4-dioxane, RT—60 °C; (d) (*R*)-alaninol, Et_3_N, ACN, 0 °C—RT; (e) 2,3-difluorobenzylamine, 1,4-dioxane, 100 °C; (f) 2,3-difluorobenzylalcohol, K_2_CO_3_, 1,4-dioxane, 120 °C; (g) thiol, NaH, 1,4-dioxane, RT—60 °C.

For the introduction of structural modifications at positions 2 and 4 of the pyrido[3,4-*d*]pyrimidine scaffold, 2,4-dichloropyrido[3,4-*d*]pyrimidine **22**, prepared according to a literature procedure [54], was selected as key intermediate (Scheme 5). As nucleophilic aromatic substitution on compound **22** is selective for the 4-position, this position was varied first while keeping the 2,3-difluorobenzylthio group fixed. Insertion of selected amines in the presence of triethylamine in DMF proceeded smoothly to give compounds derived from (*R*)-2-aminobutan-1-ol (compound **23a**), 2-amino-2-methylpropan-1-ol (compound **23b**), 3-aminopropan-1,2-diol (compound **23c**) and aminoethanol (compound **23d**) in good yields. For introduction of ethylene glycol, the sodium salt of ethylene glycol, prepared by treating dry ethylene glycol with NaH, was added dropwise to a solution of compound **22** at 0 °C. Despite this controlled addition, the yield of 2-[(2-chloro-pyrido[3,4-*d*]pyrimidine-4-yl)oxy]ethanol **23e** was only moderate. Finally, introduction of the 2,3-difluorobenzylthio moiety on 2-chloropyrido[3,4-*d*]pyrimidines **23a**–**e** proceeded in high yields affording target compounds **24a**–**e**.

In another series, the 2-position substituent was varied while retaining the (*R*)-alaninol group at position 4. The introduction of the (*R*)-alaninol substituent on intermediate **22** gave the key intermediate **25**. This was aminated with a stoichiometric amount of 2,3-difluorobenzylamine, giving the 2,4-diaminopyrido[3,4-*d*]pyrimidine **26a** in good yield. Introduction of 2,3-difluorobenzylalcohol required using the alcohol nucleophile as cosolvent and a long reaction time. Even then, the reaction did not proceed to full conversion, resulting in tedious purification and a low yield of compound **26b**. For the thiol nucleophiles (thiophenol (compound **26c**), phenylethanethiol (compound **26d**), and hexanethiol (compound **26e**)), the nucleophilic aromatic substitution proceeded smoothly and the target compounds **26c**–**e** were isolated in high yields.

### 2.2. Biological Evaluation

All pyrido[3,4-*d*]pyrimidines were evaluated as potential CXCR2 antagonists in an in vitro cell-based calcium mobilization assay. Stimulating human glioblastoma U87 cells overexpressing CXCR2 with CXCL8 results in increased intracellular calcium levels, allowing for the determination of functional CXCR2 antagonism. Navarixin, a well-known CXCR2 antagonist, was included as positive control, giving an IC_50_ value of 0.0049 µM, in agreement with literature values [55]. Since the original pyrido[3,4-*d*]pyrimidine lead compound **2** was derived from a thiazolo[4,5-*d*]pyrimidine scaffold, the 2-amino-thiazolo[4,5-*d*]pyrimidine analogue, endowed with an IC_50_ value of 0.079 µM, was also included for comparison purposes.

In a first round of SAR, the substitution pattern of the pyridine moiety of the pyrido[3,4-*d*]pyrimidine scaffold was explored (Table 1). The rationale of selecting halogens was driven by the fact that they can function as handle for subsequent introduction of structural diversity by palladium-catalyzed cross couplings or nucleophilic aromatic substitutions. The 5-chloro compound **9** was one hundred fold less potent as CXCR2 antagonist (IC_50_ = 11 µM) when compared to the original hit **2**. Synthesis problems (vide supra) prevented us from further elaboration of this particular position. The 6-chloro analogue **16a** was completely devoid of CXCR2 antagonism. Further elaboration at the 6-position by the synthesis of a 6-*N*-imidazolyl, 6-phenyl- and 6-thienyl-pyrido[3,4-*d*]pyrimidine analogue (compounds **16b**, **17a** and **17d**) did not have any beneficial effect on CXCR2 antagonism. Remarkably, the 6-furanyl congener **17b** (IC_50_ = 0.54 µM) showed comparable activity to its unsubstituted congener **2** (IC_50_ = 0.11 µM). However, introduction of a methyl group on the furanyl group (compound **17c**) led again to complete loss of activity. It is well known that an amino group on the thiazolo moiety of thiazolo[4,5-*d*]pyrimidine yields potent CXCR2 antagonists.[37] In contrast, within the pyrido[3,4-*d*]pyrimidine series, the presence of an amino group at position 6 (compound **21**) led to a complete loss of CXCR2 antagonistic activity. This suggests that the structure–activity relationship (SAR) in the thiazolo[4,5-*d*]pyrimidines and pyrido[3,4-*d*]pyrimidines is different and does not run in parallel. Although compounds **2** and **17b** are the most potent CXCR2 antagonists within this series, they are still clearly much less active than navarixin and the reference thiazolo[4,5-*d*]pyrimidine analogue.

Since the 5- and 6-substituted derivatives displayed strongly reduced activity compared to the unsubstituted pyrido[3,4-*d*]pyrimidine **2**, structural modifications of the substituents at positions 2 and 4 were explored. Subtle structural variations of the (*R*)-alaninol group of hit compound **2** (Table 2), such as elongation of the methyl to an ethyl group (compound **24a**), insertion of an additional methyl group yielding the *gem*-dimethyl analogue **24b**, introducing a hydroxyl functionality (compound **24c**) or removal of the methyl group (compound **24d**), afforded pyrido[4,3-*d*]pyrimidines that were completely devoid of CXCR2 antagonism, as evidenced by IC_50_ values exceeding 30 µM. Similarly, an ethyleneglycol linker at position 4 gave compound **24e** also lacking CXCR2 antagonism. This situation is different from the thiazolo[4,5-*d*]pyrimidine series, since for this latter scaffold, CXCR2 antagonism tolerates structural variation at this position [37].

To probe the importance of the sulfur linker for CXCR2 antagonism, the corresponding amino and oxygen linkers were prepared, yielding compounds **26a** and **26b**, respectively, that were both devoid of CXCR2 antagonistic activity. Since a sulfur was found to be important for imparting CXCR2 antagonism, a limited number of thiols was appended to position 2 of the pyrido[3,4-*d*]pyrimidine scaffold. Shortening (compound **26c**) or elongation (compound **26d**) of the linker between the sulfur and the phenyl moiety furnished analogues that were inactive as CXCR2 antagonists. Since it has been shown before that in the thiazolo[4,5-*d*]pyrimidine series, an aliphatic side chain is tolerated for CXCR2 antagonism, an *n*-hexyl linker was installed (compound **26e**), which was inactive as CXCR2 antagonist, with an IC_50_ value exceeding 30 µM (Table 3).

## 3. Materials and Methods

### 3.1. Materials

#### 3.1.1. Chemistry

All chemicals were purchased from Acros Organics (Geel, Belgium), Merck (Darmstadt, Germany), Alfa Aesar (Kandel, Germany), Fluorochem (Hadfield, UK) and TCI Europe (Zwijndrecht, Belgium) and used as received. Moisture sensitive reactions were carried out under nitrogen or argon-atmosphere, using flame dried glassware. Reactions were stirred magnetically. Reaction conversion was monitored via TLC analysis using MilliporeSigma™ Silica Gel 60 F254 Coated Aluminum-Backed TLC Sheets or Macherey-Nagel SILPre-coated ALUGRAM^®^ Xtra SIL G/UV_254_ TLC sheets. Compounds were visualized via UV irradiation (254 nm), iodine coated silica or KMnO_4_-staining. Column chromatography was performed via standard column chromatography or using an MPLC apparatus. For column chromatography, 70–230 mesh silica 60 (Acros, Geel, Belgium) was used as the stationary phase. MPLC was performed using a CombiFlash EZ prep apparatus with BGB Scorpius Silica 60Å Irregular—50 µm cartridges. ^1^H NMR spectra were recorded on a Bruker Avance 300 (300 MHz working frequency) or Bruker Avance 400 (400 MHz working frequency). Proton-decoupled ^13^C NMR spectra were recorded on a Bruker Avance 400 (101 MHz working frequency). ^19^F NMR spectra were recorded on a Bruker Avance 400 (377 MHz working frequency). Samples were dissolved in d_6_-DMSO or CDCl_3_, and chemical shifts (δ) were reported in parts per million (ppm) referenced to tetramethylsilane (^1^H), or the internal (NMR) solvent signal (^1^H and ^13^C) as internal standards [56]. All NMR spectra are presented in Supplementary Materials. High-resolution mass spectra were acquired on a quadrupole orthogonal acceleration time-of-flight mass spectrometer (Synapt G2 HDMS, Waters, Milford, MA, USA). Samples were infused at 3 μL/min and spectra were obtained in positive ionization mode with a resolution of 15,000 (FWHM—full width at half maximum) using leucine enkephalin as a lock mass. Melting points were determined using a Reichert Thermovar apparatus and are uncorrected.

#### 3.1.2. Biology

Recombinant human CXCL8 was obtained from Peprotech (Rocky Hill, NJ, USA).

### 3.2. Methods

#### 3.2.1. Synthesis

**3-Amino-5-chloropyridine-4-carbonitrile (5)**

A mixture of 3,5-dichloropyridine-4-carbonitrile **3** (13.76 g, 80.0 mmol, 1.00 eq.) and NaN_3_ (5.72 g, 8.80 mmol, 1.10 eq.) in DMF (100 mL) was heated to 80 °C for 19 h. After cooling to room temperature, the reaction mixture was transferred to a separatory funnel together with H_2_O (200 mL) and EtOAc (200 mL). After phase separation the aqueous phase was extracted with EtOAc (2 × 200 mL). The combined organic phases were washed with water (2 × 200 mL) and brine (2 × 200 mL), dried over Na_2_SO_4_ and coated on Celite. Filtration over silica using 30% EtOAc/PE gave the crude intermediated product **4a**, which was used without further purification in the next step. The obtained solid was dissolved ACN (600 mL) and NaI (107 g, 720 mmol, 9.00 eq.) was added. The suspension was cooled in an ice bath and FeCl_3_ (19.4 g, 120 mmol, 1.50 eq.) was added. After stirring for 10 min at 0 °C, the mixture was stirred for 30 min at room temperature, when full conversion was observed by TLC. The reaction mixture was transferred to a separatory funnel along with EtOAc (200 mL) and water (100 mL) and quenched with Na_2_S_2_O_3_. After phase separation, the aqueous phase was extracted with EtOAc (2 × 200 mL). The combined organic phases were washed with H_2_O (2 × 100 mL) and brine (2 × 100 mL), dried over Na_2_SO_4_ and filtered over silica. The obtained brown solid was recrystallized twice from methanol to afford the title compound (11.041 g, 71.89 mmol, 90%) as an off-white solid. mp 170—172 °C.

^1^H NMR (400 MHz, d_6_-DMSO): δ (ppm) 8.14 (s, 1H), 7.85 (s, 1H), 6.85 (s, 2H); ^13^C NMR (101 MHz, d_6_-DMSO): δ (ppm) 147.9, 137.5, 133.8, 130.8, 113.5, 98.7.

**3-Amino-5-chloropyridine-4-carboxamide (6)**

3-Amino-5-chloropyridine-4-carbonitrile (9.55 g, 62.2 mmol, 1.00 eq.), which had been finely ground with a pestle and mortar, was added to solution of 50% aqueous CsOH (1.4 mL, 7.8 mmol, 13 mol%) in 20% aqueous ammonia (80 mL) in a 100 mL screw-capped reaction tube equipped with a stir bar. After heating to 100 °C for 1 h, all material had dissolved, and the mixture was cooled to 0 °C in an ice bath. The resulting white crystalline precipitate was filtered and washed with ice water and dried under vacuum (40 °C) to afford the title compound as a white solid (7.957 g, 46.37 mmol, 75%).

^1^H NMR (400 MHz, d_6_-DMSO): δ (ppm) 7.99 (s, 1H), 7.82 (br. s, 2H), 7.77 (s, 1H), 5.50 (br. s, 2H); ^13^C NMR (101 MHz, d_6_-DMSO): δ (ppm) 165.5, 142.3, 136.2, 135.3, 127.1, 126.7.

**2-Thioxo-5-chloropyrido[3,4-*d*]pyrimidine-4-one (7)**

DBU (5.0 mL, 33.5 mmol, 2.3 eq.) was added dropwise to a suspension of 2-chloro-5-aminopyridine-4-carboxamide (2.50 g, 14.6 mmol, 1.00 eq.) in dry DMF (25 mL) and CS_2_ (5.0 mL, 83 mmol, 5.7 eq.), under N_2_-atmosphere, while cooling in an ice bath. Following complete addition, the reaction mixture was heated to 60 °C for 3 h and then cooled in an ice bath, and poured over 100 mL ice. Next, the reaction mixture was acidified (pH = 2–3) with 1M HCl under continuous stirring. The obtained precipitate was washed with H_2_O, MeOH, and EtOAc, and dried under vacuum to afford the title compound (2.783 g, 13.0 mmol, 89%) as a green solid that was used without further purification. mp > 300 °C.

**2-(2,3-Difluorobenzylmercapto)-5-chloropyrido[3,4-*d*]pyrimidine-4-one (8)**

2-Thioxo-5-chloropyrido[3,4-*d*]pyrimidine-4-one (427 mg, 2.00 mmol, 1.00 eq.) and dry DMF (20 mL) were added to a flame dried, N_2_-flushed 100 mL round-bottom flask under N_2_-atmosphere. The suspension was sonicated for 15 min, after which triethylamine (0.3 mL, 2.2 mmol, 1.1 eq.) was added, resulting in dissolution of the starting material. Next, a solution of 2,3-difluorobenzyl bromide (414 mg, 2.00 mmol, 1.00 eq.) in dry DMF (5 mL) was added dropwise. The reaction mixture was stirred at room temperature for 1 h, concentrated in vacuo and 20 mL H_2_O was added. The formed precipitate was filtered, washed with water, MeOH and Et_2_O, and dried under vacuum to afford the title compound (621 mg, 1.83 mmol, 92%) as a beige solid. mp > 300 °C.

^1^H NMR (400 MHz, d_6_-DMSO): δ (ppm) 13.04 (br. s, 1H), 8.85 (s, 1H), 8.50 (s, 1H), 7.51–7.42 (app. t, *J* = 7.0 Hz, 1H), 7.39–7.28 (m, 1H), 7.22–7.11 (m, 1H), 4.55 (s, 2H); HRMS (ESI-Q-TOF): *m*/*z* [M+H]^+^ calcd. for C_14_H_8_Cl_1_F_2_N_3_O_1_S_1_: 340.01173; found: 340.0114.

**(2*R*)-2-[(5-Chloro-2-(2,3-difluorobenzylmercapto)-pyrido[3,4-*d*]pyrimidine-4-yl)amino]propanol (9)**

In a flame dried, N_2_-flushed screw capped reaction tube 2-(2,3-difluorobenzylmercapto)-5-chloropyrido[3,4-*d*]pyrimidine-4-one (600 mg, 1.76 mmoL, 1.00 eq.) and POCl_3_ (10 mL) were added. After heating to 120 °C for 2 h, the mixture was cooled down to room temperature, and concentrated to dryness in vacuo. The obtained material was resuspended in ethyl acetate, poured over ice and neutralized with saturated aqueous NaHCO_3_. The obtained aqueous solution was extracted with ethyl acetate, and the combined organic phases were dried over MgSO_4_ and concentrated in vacuo. The crude intermediate was then dissolved in dry DMF (6 mL), and Et_3_N (0.50 mL, 3.6 mmol, 2.0 eq.) and (*R*)-alaninol (0.50 mL, 6.40 mmol, 3.6 eq.) were added dropwise while cooling in an ice bath. After stirring overnight at room temperature, the reaction mixture was concentrated in vacuo. Water (20 mL) and EtOAc (20 mL) were added. Following phase separation, the aqueous phase was extracted with EtOAc (2 × 20 mL). The combined organic phases were washed with water (2 × 20 mL) and brine (2 × 20 mL), dried over Na_2_SO_4_ and coated on Celite. Filtration over silica using 1:1 EtOAc/Et_2_O followed by column chromatography using 40–100% Et_2_O/PE afforded the title compound (61 mg, 0.15 mmol, 9%) as a white solid. mp 146–148 °C.

^1^H NMR (400 MHz, d_6_-DMSO): δ (ppm) 8.83 (s, 1H), 8.43 (s, 1H), 8.04 (d, *J* = 7.4 Hz, 1H), 7.45–7.37 (app t., *J* = 7.1 Hz, 1H), 7.34–7.23 (m, 1H), 7.19–7.09 (m, 1H), 5.15 (t, *J* = 4.5 Hz, 1H), 4.47 (s, 2H), 4.37–4.24 (m, 1H), 3.64–3.48 (m, 2H), 1.21 (d, *J* = 6.6 Hz, 3H); ^13^C NMR (101 MHz, d_6_-DMSO): δ (ppm) 168.0, 156.2, 149.6 (dd, *J* = 12.6, 245.4 Hz), 149.5, 148.1 (12.9, 246.8 Hz), 146.3, 142.9, 127.9 (d, *J* = 11.1 Hz), 126.2 (t, *J* = 2.6 Hz), 124 (t, *J* = 2.6 Hz), 124.6 (d, *J* = 4.7, 7.0 Hz), 124.5, 116.2 (d, *J* = 11.1 Hz), 114.3, 63.0, 48.9, 27.3, 16.3; ^19^F NMR (377 MHz, d_6_-DMSO): δ (ppm) −139.30 (m, 1F), −142.74 (m, 1F); HRMS (ESI-Q-TOF): *m*/*z* [M+H]^+^ calcd. for C_17_H_15_Cl_1_F_2_N_4_O_1_S_1_: 397.06958; found: 397.0697.

**2-Bromo-5-nitropyridine-4-carboxylic acid (11)**

2-Bromo-4-methyl-5-nitropyridine **10** (25.0 g, 115 mmol, 1.00 eq.) was dissolved in concentrated H_2_SO_4_ (100 mL). The mixture was cooled in an ice bath, and K_2_Cr_2_O_7_ (50.83 g, 173 mmol, 1.5 eq.) was added portion-wise. Following complete addition, the mixture was stirred in an ice bath for 1 h and at room temperature for 24 h. The resulting green solution was slowly poured onto 1L of ice. The obtained precipitate was filtered, washed with water until the eluent was no longer green, and dried under vacuum to afford the title compound as white solid (22.780 g, 92.23 mmol, 80%).

^1^H NMR (400 MHz, d_6_-DMSO): δ (ppm) 13.03 (br. s, 1H), 9.11 (s, 1H), 8.14 (s, 1H); ^13^C NMR (101 MHz, d_6_-DMSO): δ (ppm) 163.7, 146.39, 146.31, 142.2, 138.5, 127.1;

**2-Chloro-5-nitropyridine-4-carboxamide (12a)**

Oxalyl chloride (20 mL) was added to an ice-cold solution of 2-bromo-5-nitropyridine-4-carboxylic acid **11** (10.00 g, 40.49 mmol) in dry DCM (200 mL) at 0 °C and under N_2_-atmosphere. After stirring for 5 min, dry DMF (0.2 mL) was added. The reaction mixture was stirred at 0 °C for 1 h and at room temperature overnight. The resulting solution was concentrated in vacuo. The obtained residue was dissolved in dry DCM (50 mL) and added dropwise to a heavily stirred, ice cold 25% aqueous ammonia solution (300 mL). After stirring for 15 min, the formed precipitate was filtered, washed with saturated aqueous NaHCO_3_ and water, and dried under vacuum overnight to afford the title compound (7.498 g, 34.71 mmol, 86%) as a white solid that was used without further purification.

**2-Imidazolyl-5-nitropyridine-4-carboxamide (12b)**

In a flame dried N_2_-flushed round-bottom 100 mL two-necked flask, equipped with a N_2_-balloon, septum and stir bar, 2-chloro-5-nitropyridine-4-carboxylic acid **11** (1 g, 4.049 mmol, 1.00 eq.), 1,1-carbonyldiimidazole (985 mg, 6.07 mmol, 1.50 eq.) and dry THF (20 mL) were combined. The reaction mixture was stirred at room temperature for 30 min, then aqueous ammonia (3.0 mL, 40 mmol, 10 eq.) was added in one portion. The mixture was stirred overnight at room temperature, and then was transferred to a separatory funnel along with EtOAc (50 mL) and water (20 mL). Following phase separation, the aqueous phase was extracted with EtOAc (1 × 20 mL). The combined organic phases were washed with water and brine, dried over Na_2_SO_4_ and concentrated to dryness in vacuo. The obtained solid was triturated with ethanol, filtered and washed with ice cold ethanol to afford the title compound as a white solid (416 mg, 1.78 mmol, 44%). mp 259–261 °C.

^1^H NMR (400 MHz, d_6_-DMSO): δ (ppm) 9.20 (s, 1H), 8.72 (s, 1H), 8.31 (br. s, 1H), 8.16 (br. s, 1H), 8.11 (s, 1H), 8.13–8.05 (m, 2H), 7.20 (s, 1H); ^13^C NMR (101 MHz, d_6_-DMSO): δ (ppm) 164.9, 151.3, 145.9, 144.0, 140.1, 136.0, 131.1, 117.0, 111.4; HRMS (ESI-Q-TOF): *m*/*z* [M+H]^+^ calcd. for C_9_H_7_N_5_O_3_: 234.06216; found: 234.0625.

**2-Chloro-5-aminopyridine-4-carboxamide (13a)**

Activated iron powder (8.31g, 149 mol, 5 eq.) was added to a mixture of 2-chloro-5-nitropyridine-4-carboxamide **12a** (6.00 g, 29.8 mmol, 1.00 eq.) in water (15 mL), EtOH (30 mL) and AcOH (30 mL). The resulting suspension was sonicated for 1 h and then filtered over 1:1 Celite/MgSO_4_, which was washed with EtOAc (500 mL). The filtrate was transferred to a separatory funnel along with water (100 mL). After phase separation, the aqueous phase was extracted with EtOAc (2 × 100 mL). The combined organic phases were washed with saturated aqueous NaHCO_3_ (3 × 100 mL), water (3 × 100 mL) and brine (2 × 100 mL), dried over Na_2_SO_4_ and coated on Celite. Flash chromatography using 50–100% EtOAc/PE afforded the title compound (4.340 g, 25.29 mmol, 85%) as an off-white solid.

^1^H NMR (400 MHz, d_6_-DMSO): δ (ppm) 8.12 (br. s, 1H), 7.93 (s, 1H), 7.57 (br. s, 1H), 7.54 (s, 1H), 6.65 (s, 2H); ^13^C NMR (101 MHz, d_6_-DMSO): δ (ppm) 168.4, 144.5, 139.3, 135.0, 121.7, 121.5.

**5-Amino-2-imidazolyl-pyridine-4-carboxamide (13b)**

In a round-bottom 100 mL two-necked flask, equipped with a hydrogen-balloon, septum and stir bar, 2-imidazolyl-5-nitropyridine-4-carboxamide **12b** (300 mg, 1.287 mmol, 1.00 eq.) and Pd/C (15 mg, 5 w%) were combined in THF (20 mL). The reaction mixture was stirred for 24 h at room temperature and filtered over Celite. The Celite was thoroughly washed with EtOAc. The solution was coated on Celite. Flash chromatography using 0–20% MeOH/EtOAc afforded the title compound (188 mg, 0.925 mmol, 72%) as an off-white solid. mp 248–251 °C.

^1^H NMR (400 MHz, d_6_-DMSO): δ (ppm) 8.28 (s, 1H), 8.14 (br. s, 1H), 8.05 (s, 1H), 7.79 (s, 1H), 7.75 (s, 1H), 7.67 (br. s, 1H), 7.08 (s, 1H), 6.67 (br. s, 2H); ^13^C NMR (101 MHz, d_6_-DMSO): δ (ppm) 168.9, 144.1, 138.2, 137.9, 134.3, 129.4, 120.8, 116.6, 111.1; HRMS (ESI-Q-TOF): *m*/*z* [M+H]^+^ calcd. for C_9_H_9_N_5_O_1_: 204.08798; found: 204.0871.

**2-Thioxo-5-chloropyrido[3,4-*d*]pyrimidine-4-one (14a)**

DBU (5.0 mL, 33.5 mmol, 2.3 eq.) was added dropwise to a suspension of 2-chloro-5-aminopyridine-4-carboxamide **13a** (2.50 g, 14.6 mmol, 1.00 eq.) in dry DMF (25 mL) and CS_2_ (5.0 mL, 83 mmol, 5.7 eq.), under N_2_-atmosphere, while cooling in an ice bath. Following complete addition, the reaction mixture was heated to 60 °C for 3 h, and then cooled in an ice bath and poured over 100 mL ice. While stirring, the reaction mixture was acidified (pH = 2–3) with 1M HCl. The obtained precipitate was washed with H_2_O, MeOH, and EtOAc and dried under vacuum to afford the title compound (2.783 g, 13.0 mmol, 89%) as a green solid that was used without further purification.

**6-Chloro-2-thioxopyrido[3,4-*d*]pyrimidine-4-one (14b)**

A suspension of 5-amino-2-imidazolyl-pyridine-4-carboxamide **13b** (150 mg, 0.738 mmol, 1.00 eq.) in dry DMF (1.2 mL), in a flame dried, N_2_-flushed reaction tube sealed with a septum screw cap, was cooled to 0 °C in an ice bath. Carbon disulfide (0.22 mL, 3.7 mmol, 5 eq.) was added in one portion and DBU (0.22 mL, 1.5 mmol, 1.5 eq.) was added dropwise. After heating to 60 °C for 3 h, the reaction mixture was cooled in an ice bath and poured onto 10 mL ice. The mixture was acidified with 1M HCl (pH 3–4) and filtered. The obtained solid was washed with water, methanol and EtOAc and dried under vacuum to afford the title compound as a yellow solid (142 mg, 0.579 mmol, 78%), which was used without further purification.

**2-(2,3-Difluorobenzylmercapto)-6-chloropyrido[3,4-*d*]pyrimidine-4-one (15a)**

2-Thioxo-6-chloropyrido[3,4-*d*]pyrimidine-4-one **14a** (4.180 g, 19.57 mmol, 1.00 eq.) and dry DMF (40 mL) were added to a flame dried, N_2_-flushed 250 mL round-bottom flask under N_2_-atmosphere. The suspension was sonicated for 15 min, after which triethylamine (3.0 mL, 22 mmol, 1.1 eq.) was added, resulting in dissolution of the starting material. Next, a solution of 2,3-difluorobenzyl bromide (4.324 g, 20.88 mmol, 1.05 eq.) in dry DMF (20 mL) was added dropwise. The reaction mixture was stirred at room temperature for 2 h, concentrated in vacuo and 50 mL H_2_O was added. The formed precipitate was filtered, washed with water (2 × 10 mL), MeOH (10 mL) and Et_2_O (10 mL) and dried under vacuum to afford the title compound (4.996 g, 14.705 mmol, 74%) as a beige solid. mp 235–237 °C

^1^H NMR (400 MHz, d_6_-DMSO): δ (ppm) 13.11 (br. s, 1H), 8.82 (s, 1H), 7.88 (s, 1H), 7.51–7.43 (m, 1H), 7.41–7.29 (m, 1H), 7.21–7.12 (m, 1H), 4.56 (s, 2H); ^19^F NMR (377 MHz, d_6_-DMSO): δ (ppm) −139.17 (m, 1F), −142.14 (m, 1F) HRMS (ESI-Q-TOF): *m*/*z* [M+H]^+^ calcd. for C_14_H_9_Cl_1_F_2_N_3_O_1_S_1_: 340.0117; found: 340.0119.

**2-(2,3-Difluorobenzylthio)-6-imidazolyl-pyrido[3,4-*d*]pyrimidine-4-one (15b)**

To a flame dried, N_2_-flushed reaction tube equipped with a stirring bar and a septum screw cap, 6-chloro-2-thioxopyrido[3,4-*d*]pyrimidine-4-one **14b** (123 mg, 0.50 mmol, 1.00 eq.), and dry DMF (2.0 mL) and Et_3_N (76 μL, 0.55 mmol, 1.1 eq.) were added. A solution of 2,3-difluorobenzyl bromide (109 mg, 0.525 mmol, 1.05 eq.) in dry DMF (1 mL) was added dropwise. After stirring at room temperature for 1 h, the reaction mixture was concentrated to dryness in vacuo, and coated on Celite. Flash chromatography using 0–10% MeOH/EtOAc afforded the title compound (95 mg, 0.26 mmol, 52%).

^1^H NMR (400 MHz, d_6_-DMSO): δ (ppm) 13.06 (br. s, 1H), 8.91 (s, 1H), 8.64 (s, 1H), 8.21 (s, 1H), 8.08 (s, 1H), 7.64–7.26 (m, 2H), 7.25–6.93 (m, 2H), 4.49 (s, 2H); ^19^F NMR (377 MHz, d_6_-DMSO): δ (ppm) −139.17 (m, 1F), −142.15 (m, 1F); HRMS (ESI-Q-TOF): *m*/*z* [M+H]^+^ calcd. for C_17_H_11_F_2_N_5_O_1_S_1_: 372.07250; found: 372.0727.

**(2*R*)-2-[(6-Chloro-2-(2,3-difluorobenzylmercapto)-pyrido[3,4-*d*]pyrimidine-4-yl)amino]propanol (16a)**

In a flame dried, N_2_-flushed screw capped reaction tube 2-(2,3-difluorobenzylmercapto)-6-chloropyrido[3,4-*d*]pyrimidine-4-one **15a** (960 mg, 2.83 mmol, 1.00 eq.) and SOCl_2_ (10 mL) and 1 drop of DMF were added. After heating to 80 °C for 4 h, the mixture was cooled down to room temperature, and concentrated to dryness in vacuo to afford the crude intermediate as a yellow solid. The intermediate was then dissolved in dry DMF (10 mL), and Et_3_N (1.00 mL, 7.2 mmol, 2.5 eq.) and (*R*)-alaninol (0.50 mL, 6.4 mmol, 2.3 eq.) were added dropwise while cooling in an ice bath. After stirring for 1 h at room temperature, the reaction mixture was concentrated to dryness in vacuo, transferred to a separatory funnel along with water (10 mL) and EtOAc (20 mL). After extracting with EtOAc (3 × 20 mL), the combined organic phases were washed with water (3 × 20 mL) and brine (2 × 20 mL), dried over Na_2_SO_4_ and coated on Celite. Flash chromatography using 50% PE/Et_2_O–1% MeOH/Et_2_O, followed by trituration with 50% pentane/Et_2_O afforded the title compound (902 mg, 2.27 mmol, 80%) as a white solid. m.p. 211–213 °C.

^1^H NMR (400 MHz, d_6_-DMSO): δ (ppm) 8.79 (s, 1H), 8.39 (d, *J* = 7.9 Hz, 1H), 8.37 (s, 1H), 7.46–7.37 (app. t, *J* = 7.1 Hz, 1H), 7.34–7.23 (m, 1H), 7.17–7.07 (m, 1H), 4.81 (t, *J* = 5.8 Hz, 1H), 4.47 (s, 2H), 4.42–4.31 (m, 1H), 3.57–3.41 (m, 2H), 1.19 (d, *J* = 6.7 Hz, 3H); ^13^C NMR (101 MHz, d_6_-DMSO): δ (ppm) 168.0, 156.9, 150.2, 149.6 (dd, *J* = 12.5, 245.5 Hz), 148.1 (dd, *J* = 12.9, 246.8 Hz), 143.7, 143.0, 128.1 (d, *J* = 11.2 Hz), 126.3 (t, *J* = 2.7 Hz), 124.5 (dd, *J* = 4.7, 6.9 Hz), 119.9, 116.3, 116.2, 116.1, 63.5, 48.8, 27.3, 16.4; ^19^F NMR (377 MHz, d_6_-DMSO): δ (ppm) −139.30 (m, 1F), −142.77 (m, 1F); HRMS (ESI-Q-TOF): *m*/*z* [M+H]^+^ calcd. for C_17_H_15_Cl_1_F_2_N_4_O_1_S_1_: 397.06958; found: 397.0693.

**(2*R*)-2-[(6-Imidazolyl-2-(2,3-difluorobenzylmercapto)-pyrido[3,4-*d*]pyrimidine-4-yl)amino]propanol (16b)**

DBU (0.05 mL, 0.3 mmol, 1.3 eq.) was added dropwise to a solution of 6-chloro-2-(2,3-difluorobenzylthio)-pyrido[3,4-*d*]pyrimidine-4-one **15b** (75 mg, 0.20 mol, 1.00 eq.) and BOP-reagent (116 mg, 0.26 mmol, 1.30 eq.) in dry DMF (1 mL) under N_2_-atmosphere. Following complete addition, the reaction mixture was stirred for 10 min, after which (*R*)-alaninol (0.05 mL, 0.6 mmol, 3 eq.) was added dropwise. The reaction mixture was stirred at room temperature overnight, and then heated to 60 °C for 2–3 h. Next, the reaction mixture was concentrated in vacuo and redissolved in 20 mL EtOAc and NH_4_Cl (10 mL) was added. The aqueous phase was extracted with EtOAc (2 × 20 mL). The combined organic phases were washed with saturated NH_4_Cl (3 × 10 mL), with water (2 × 10 mL) and brine (2 × 10 mL), and dried over Na_2_SO_4_. After coating on Celite, flash chromatography using 0–5% MeOH/DCM afforded the title compound (51 mg, 0.12 mmol, 60%) as a white-yellow solid. mp 245–247 °C.

^1^H NMR (400 MHz, d_6_-DMSO): δ (ppm) 8.89 (s, 1H), 8.53 (s, 1H), 8.48 (s, 1H), 8.32 (d, *J* = 7.2 Hz, 1H), 7.90 (s, 1H), 7.49–7.38 (m, 1H), 7.37–7.25 (m, 1H), 7.24–7.06 (m, 2H), 4.91 (s, 1H), 4.50 (s, 2H), 4.35–4.34 (m, 1H), 3.62–3.45 (m, 2H), 1.23 (d, *J* = 6.4 Hz, 3H); ^13^C NMR (101 MHz, d_6_-DMSO): δ (ppm) 167.1, 157.5, 149.6 (dd, *J* = 12.5, 245.3 Hz), 149.4, 148.1 (dd, *J* = 12.7, 246.7 Hz), 143.5, 143.4, 130.2, 128.2 (d, *J* = 11.1 Hz), 127.3, 126.4 (t, *J* = 2.7 Hz), 124.6 (dd, *J* = 4.5, 7.1 Hz), 119.6, 116.2 (d, *J* = 16.8 Hz), 109.6, 104.1, 63.5, 48.8, 27.3, 16.5; ^19^F NMR (377 MHz, d_6_-DMSO): δ (ppm) −139.33 (m, 1F), −142.79 (m, 1F); HRMS (ESI-Q-TOF): *m*/*z* [M+H]^+^ calcd. for C_20_H_18_F_2_N_6_O_1_S_1_: 429.13035; found: 429.1311.

**(2*R*)-2-[(2-(2,3-Difluorobenzylmercapto)-6-phenyl-pyrido[3,4-*d*]pyrimidine-4-yl)amino]propanol (17a)**

To a flame dried, N_2_-flushed 8 mL reaction tube equipped with a stir bar and a septum screw cap, (2*R*)-2-[(6-chloro-2-(2,3-difluorobenzylmercapto)-pyrido[3,4-*d*]pyrimidine-4-yl)amino]propanol **16a** (100 mg, 0.250 mmol, 1.00 eq.) K_2_CO_3_ (104 mg, 0.75 mmol, 3.00 eq.), 2-phenylboronic acid (56 mg, 0.50 mmol, 2.0 eq.) and Pd(PPh_3_)_4_ (30 mg, 0.025 mmol, 10 mol%) were added. Following three cycles of backfilling with nitrogen, 1,4-dioxane (3.0 mL) and water (1.0 mL) were added. After heating to 120 °C for 48 h, the reaction mixture was transferred to a separatory funnel and water (10 mL) was added. The aqueous phase was extracted with EtOAc (3 × 20 mL). The combined organic phases were washed with saturated aqueous NaHCO_3_, water and brine, dried over Na_2_SO_4_ and coated on Celite. Flash chromatography using 20–500% EtOAc/isohexane gave a mixture of starting material and title compound (70 mg, in a 1:9 ratio). This crude product was recrystallized by dissolving in EtOAc and layering with pentane. The obtained crystals were filtered and washed with 1:1 Et_2_O/pentane to afford the title compound (42 mg, 0.096 mmol, 38%) as an off-white/light-yellow solid. mp 202–204 °C.

^1^H NMR (400 MHz, d_6_-DMSO): δ (ppm) 9.02 (s, 1H), 8.75 (s, 1H), 8.47 (d, *J* = 7.8 Hz, 1H), 8.24–8.16 (m, 2H), 7.54 (t, *J* = 7.6 Hz, 2H), 7.49–7.41 (m, 2H), 7.38–7.27 (m, 1H), 7.20–7.10 (m, 1H), 4.87 (t, *J* = 5.7 Hz, 1H), 4.52 (s, 2H), 4.48–4.38 (m, 1H), 3.60–3.46 (m, 2H), 1.24 (d, *J* = 6.7 Hz, 3H); ^13^C NMR (101 MHz, d_6_-DMSO): δ (ppm) 167.2, 157.9, 149.9, 149.6 (dd, *J* = 12.3, 245.1 Hz), 148.2 (dd, *J* = 12.9, 246.6 Hz), 143.6, 138.2, 128.7, 128.3 (d, *J* = 11.0 Hz), 126.3, 124.6 (dd, *J* = 4.9, 6.7 Hz), 119.1, 116.1 (d, *J* = 16.8 Hz), 111.6, 63.6, 48.7, 27.2, 16.6;

^19^F NMR (377 MHz, d_6_-DMSO): δ (ppm) −139.35 (m, 1F), −142.82 (m, 1F); HRMS (ESI-Q-TOF): *m*/*z* [M+H]^+^ calcd. for C_23_H_20_F_2_N_4_O_1_S_1_: 439.13985; found: 439.1395.

**(2*R*)-2-[(2-(2,3-Difluorobenzylmercapto)-6-(2-furanyl)-pyrido[3,4-*d*]pyrimidine-4-yl)amino]propanol (17b)**

To a flame dried, N_2_-flushed 8 mL reaction tube equipped with a stir bar and a septum screw cap, (2R)-2-[(6-chloro-2-(2,3-difluorobenzylmercapto)-pyrido[3,4-*d*]pyrimidine-4-yl)amino]propanol **16a** (100 mg, 0.250 mmol, 1.00 eq.) K_2_CO_3_ (104 mg, 0.75 mmol, 3.00 eq.), 2-furanyl boronic acid (56 mg, 0.50 mmol, 2.0 eq.) and Pd(PPh_3_)_4_ (30 mg, 0.025 mmol, 10 mol%) were added. Following three cycles of backfilling with nitrogen, 1,4-dioxane (3.0 mL) and water (1.0 mL) were added. After heating to 120 °C for 48 h, the reaction mixture was transferred to a separatory funnel, and water (10 mL) was added. The aqueous phase was extracted with EtOAc (3 × 20 mL). The combined organic phases were washed with saturated aqueous NaHCO_3_, water and brine, and dried over Na_2_SO_4_. After coating on Celite, flash chromatography using 70–100% Et_2_O/PE to 1–2 MeOH/Et_2_O afforded the title compound (43 mg, 0.10 mmol, 40%) as a light brown solid. mp 163–165 °C.

^1^H NMR (400 MHz, d_6_-DMSO): δ (ppm) 8.91 (s, 1H), 8.54 (d, *J* = 7.9 Hz, 1H), 8.50 (s, 1H), 7.86 (d, *J* = 1.0 Hz, 1H), 7.47–7.38 (m, 1H), 7.34–7. 23 (m, 1H), 7.17–7.09 (m, 1H), 7.06 (d, *J* = 3.2 Hz, 1H), 6.68 (d, *J* = 1.8 Hz, 1H), 6.68 (dd, *J* = 1.8, 3.3 Hz, 1H), 4.84 (t, *J* = 5.7 Hz, 1H), 4.49 (s, 2H), 4.45–4.36 (m, 1H), 3.63–3.53 (m, 1H), 3.53–3.44 (m, 1H), 1.21 (d, *J* = 6.7 Hz, 3H); ^13^C NMR (101 MHz, d_6_-DMSO): δ (ppm) 167.1, 157.7, 153.3, 150.2, 149.6 (dd, *J* = 12.5, 245.3 Hz), 148.2 (dd, *J* = 12.9, 246.8 Hz), 143.8, 143.5, 142.7, 128.3 (d, *J* = 11.4 Hz), 126.3 (t, *J* =2.7 Hz), 124.6 (dd, *J* = 4.7, 6.9 Hz), 117.9, 116.1 (d, *J* = 16.8 Hz), 112.5, 109.6, 108.4, 63.7, 48.7, 27.2, 16.5; ^19^F NMR (377 MHz, d_6_-DMSO): δ (ppm) −139.32 (m, 1F), −142.80 (m, 1F);

HRMS (ESI-Q-TOF): *m*/*z* [M+H]^+^ calcd. for C_21_H_18_F_2_N_4_O_2_S_1_: 429.1191; found: 429.1195.

**(2*R*)-2-[(2-(2,3-Difluorobenzylmercapto)-6-(5-methylfuran-2-yl)-pyrido[3,4-*d*]pyrimidine-4-yl)amino]propanol (17c)**

To a flame dried, N_2_-flushed 8 mL reaction tube equipped with a stir bar and a septum screw cap, (2R)-2-[(6-chloro-2-(2,3-difluorobenzylmercapto)-pyrido[3,4-*d*]pyrimidine-4-yl)amino]propanol **16a** (100 mg, 0.250 mmol, 1.00 eq.) K_2_CO_3_ (104 mg, 0.75 mmol, 3.00 eq.), 2-furanyl boronic acid (56 mg, 0.50 mmol, 2.0 eq.) and Pd(PPh_3_)_4_ (30 mg, 0.025 mmol, 10 mol%) were added. Following three cycles of backfilling with nitrogen, 1,4-dioxane (3.0 mL) and water (1.0 mL) were added. After heating to 120 °C for 10 h, the reaction mixture was transferred to a separatory funnel, and water (10 mL) was added. The aqueous phase was extracted with EtOAc (3 × 20 mL). The combined organic phases were washed with saturated aqueous NaHCO_3_, water and brine, and dried over Na_2_SO_4_. After coating on Celite, flash chromatography using EtOAc/DCM afforded the title compound (17 mg, 0.038 mmol, 15%) as an off-white solid. mp 162–164 °C.

^1^H NMR (400 MHz, d_6_-DMSO): δ (ppm) 8.89 (s, 1H), 8.50 (d, *J* = 7.9 Hz, 1H), 8.37 (s, 1H), 7.48–7.38 (m, 1H), 7.36–7.25 (m, 1H), 7.19–7.09 (m, 1H), 6.96 (d, *J* = 3.1 Hz, 1H), 6.30 (dd, *J* = 0.8, 3.1 Hz, 1H), 4.83 (t, *J* = 5.7 Hz, 1H), 4.50 (s, 2H), 4.47–4.35 (m, 1H), 3.61–3.52 (m, 1H), 3.51–3.44 (m, 1H), 2.42 (s, 3H), 1.21 (d, *J* = 6.8 Hz, 3H); ^13^C NMR (101 MHz, d_6_-DMSO): δ (ppm) 166.7, 157.7, 153.0, 151.8, 150.2, 149.6 (dd, *J* = 12.5, 245.1 Hz), 148.2 (dd, *J* = 12.6, 246.8 Hz), 143.2, 142.9, 128.3 (d, *J* = 11.2 Hz), 126.3 (t, *J* = 2.8 Hz), 124.6 (dd, *J* = 4.5, 7.2 Hz), 118.0, 116.1 (d, *J* = 16.8 Hz), 109.5, 108.7 (d, *J* = 15.1 Hz), 63.6, 48.7, 27.2, 16.5, 13.7;

^19^F NMR (377 MHz, d_6_-DMSO): δ (ppm) −139.36 (m, 1F), −142.83 (m, 1F); HRMS (ESI-Q-TOF): *m*/*z* [M+H]^+^ calcd. for C_22_H_20_F_2_N_4_O_2_S_1_: 443.13476; found: 443.1345.

**(2*R*)-2-[(2-(2,3-Difluorobenzylmercapto)-6-(2-thienyl)-pyrido[3,4-*d*]pyrimidine-4-yl)amino]propanol (17d)**

To a flame dried, N_2_-flushed 8 mL reaction tube equipped with a stir bar and a screw cap septum, Pd_2_(dba)_3_ (6 mg, 7 μmol, 3 mol%), XPhos (24 mg, 0.05 mmol, 20 mol%) (2R)-2-[(6-chloro-2-(2,3-difluorobenzylmercapto)-pyrido[3,4-*d*]pyrimidine-4-yl)amino]propanol **16a** (100 mg, 0.250 mmol, 1.00 eq.), 2-thienyl boronic acid (65 mg, 0.50 mmol, 2.0 eq.), and K_3_PO_4_ (107 mg, 0.50 mmol, 2.00 eq.) were added. Following three cycles of backfilling with nitrogen, degassed nBuOH (2.0 mL) was added. After heating to 120 °C for 20 h, the mixture was concentrated in vacuo, and transferred to a separatory funnel along with saturated NH_4_Cl (8 mL), H_2_O (2 mL) and EtOAc (20 mL). Following phase separation, the mixture was extracted with EtOAc (1 × 20 mL). The combined organic phases were washed with water (10 mL) and brine (10 mL), and dried over Na_2_SO_4_. After coating on Celite. Flash chromatography using 5–40% EtOAc/isohexane afforded the title compound (49 mg, 0.11 mmol, 44%) as an off-white solid. mp 176–178 °C.

^1^H NMR (400 MHz, d_6_-DMSO): δ (ppm) 8.89 (s, 1H), 8.65 (s, 1H), 8.35 (d, *J* = 7.8 Hz, 1H), 7.77 (dd, *J* = 0.7, 3.5 Hz, 1H), 7.63 (dd, *J* = 0.7, 5.0 Hz, 1H), 7.47–7.40 (m, 1H), 7.36–7.26 (m, 1H), 7.22 (d, *J* = 3.7, 5.0 Hz, 1H), 7.18–7.09 (m, 1H), 4.89 (t, *J* = 5.6 Hz, 1H), 4.50 (s, 2H), 4.46–4.35 (m, 1H), 3.62.–3.45 (m, 2H), 1.24 (d, *J* = 6.7 Hz, 3H); ^13^C NMR (101 MHz, d_6_-DMSO): δ (ppm) 166.9, 157.7, 149.8, 149.6 (dd, *J* = 12.6, 245.3 Hz), 148.2 (dd, *J* = 12.8, 246.6 Hz), 146.0, 144.3, 143.4, 128.4, 128.2 (d, *J* = 11.2 Hz), 127.9, 126.3 (t, *J*= 2.7 Hz), 124.6 (d, *J* = 4.6, 7.0 Hz), 124.4, 118.1, 116.2 (d, *J* = 16.8 Hz), 109.9, 63.6, 48.7, 27.3, 16.5; ^19^F NMR (377 MHz, d_6_-DMSO): δ (ppm) −139.32 (m, 1F), −142.80 (m, 1F); HRMS (ESI-Q-TOF): *m*/*z* [M+H]^+^ calcd. for C_21_H_18_F_2_N_4_O_1_S_2_: 445.09627; found: 445.0955.

**6-Chloro-2-(2,3-difluorobenzylmercapto)-4-(4-methoxybenzyl)-pyrido[3,4-*d*]pyrimidine (18)**

To a flame dried N_2_-flushed reaction tube equipped with a screw-cap septum and a stirring bar, 2-(2,3-difluorobenzylmercapto)-6-chloropyrido[3,4-*d*]pyrimidin-4-one **15a** (1.359 g, 4.00 mmol, 1.00 eq.), K_2_CO_3_ (0.829 g, 6.00 mmol, 1.5 eq.) and dry DMF (10 mL) were added, resulting in the formation of a clear orange solution. 4-Methoxybenzyl chloride (0.65 mL, 4.8 mmol, 1.2 eq.) was added dropwise while stirring at room temperature. The mixture was stirred at room temperature for 1 h. After a few minutes, a white precipitate started to form. After stirring at 60 °C for 22 h, the mixture was cooled to room temperature; 20 mL ice water was added. The resulting precipitate was filtered, and washed with water, methanol and diethyl ether. After drying in vacuo, the obtained solid was purified via flash chromatography using 40% EtOAc/PE to afford the title compound as a white solid (1.826, 3.970 mmol, 99%). mp 137–139 °C.

^1^H NMR (400 MHz, d_6_-DMSO): δ (ppm) 8.89 (s, 1H), 7.97 (s, 1H), 7.51–7.42 (app. t, *J* = 7.1 Hz, 1H), 7.40–7.29 (m, 1H), 7.21 (d, *J* = 8.6 Hz, 2H), 7.19–7.10 (m, 1H), 6.89 (d, *J* = 8.7 Hz, 2H), 5.21 (s, 2H), 4.61 (s, 2H), 3.71 (s, 3H); ^13^C NMR (101 MHz, d_6_-DMSO): δ (ppm) 159.2, 158.9, 158.7, 149.6, 145.2, 140.8, 128.5, 127.0, 126.7, 126.6, 126.2 (d, *J* = 9.9 Hz), 124.7, 119.0, 116.8 (d, *J* = 16.6 Hz), 113.9, 55.1, 48.9, 29.1; ^19^F NMR (377 MHz, d_6_-DMSO): δ (ppm) −139.15 (m, 1F), −141.77 (m, 1F); HRMS (ESI-Q-TOF): *m*/*z* [M+H]^+^ calcd. for C_22_H_16_Cl_1_F_2_N_3_O_2_S_1_: 460.0693; found: 460.0690.

**2-(2,3-Difluorobenzylmercapto)-4-(4-methoxybenzyl)-(*N*-diphenylmethylene)-pyrido[3,4-*d*]pyrimidine-6-amine (19)**

To a flame dried, N_2_-flushed 8 mL reaction tube equipped with a stir bar and a septum screw cap, Pd(OAc)_2_ (23 mg, 0.10 mmol, 10 mol%), (±)-BINAP (93 mg, mg, 0.15 mmol, 15 mol%), 6-chloro-2-(2,3-difluorobenzylmercapto)-4-(4-methoxybenzyl)-pyrido[3,4-*d*]pyrimidine **18** (460 mg, 1.00 mmol, 1.00 eq.) and Cs_2_CO_3_ (456 mg, 1.40 mmol, 1.40 eq.) were added. Following three cycles of backfilling with N_2_, dry 1,4-dioxane (6.0 mL) and benzophenone imine (370 mg, 2.04 mmol, 2.04 eq.) were added. The mixture was heated to 120 °C for 24 h, then cooled to room temperature, and transferred to a separatory funnel. Water (20 mL) was added, and the aqueous phase was extracted with EtOAc (3 × 20 mL). The combined organic phases were washed with water and brine, dried over Na_2_SO_4_ and coated on Celite. Flash chromatography using 0–40% EtOAc/isohexane afforded the title compound (392 mg, 0.648 mmol, 65%) as an orange solid. mp 188–191 °C.

^1^H NMR (400 MHz, d_6_-DMSO): δ (ppm) 8.73 (d, *J* = 0.8 Hz, 1H), 7.72 (d, *J* = 7.4 Hz, 2H), 7.63–7.55 (m, 1H), 7.55–7.47 (m, 2H), 7.46–7.40 (m, 1H), 7.39–7.27 (m, 4H), 7.25–7.09 (m, 6H), 6.90–6.83 (m, 2H), 5.14 (s, 2H), 4.56 (s, 2H), 3.71 (s, 3H); ^1^H NMR (400 MHz, CDCl_3_): δ (ppm) 8.80 (s, 1H), 7.85 (d, *J* = 7.2 Hz, 2H), 7.57–7.41 (m, 3H), 7.39–7.17 (m, 9H), 7.15–6.99 (m, 2H), 6.89–6.81 (m, 2H), 5.22 (s, 2H), 4.56 (s, 2H), 3.79 (s, 3H); ^13^C NMR (101 MHz, d_6_-DMSO): δ (ppm) 171.3, 160.9, 160.5, 159.4, 155.8, 150.6 (dd, *J* = 12.6, 248.6 Hz), 149.3 (dd, *J* = 13.0, 249.8 Hz), 148.6, 138.8, 138.2, 136.0, 132.5, 131.5, 130.1, 129.9, 129.6, 129.4, 129.1, 128.3, 128.2, 127.0, 126.15 (t, *J* = 3.2 Hz), 126.05 (d, *J* = 10.6 Hz), 124.08 (dd, *J* = 4.8, 6.7 Hz), 116.8 (d, *J* = 17.0 Hz), 114.0, 109.6, 55.3, 47.3, 29.7; ^19^F NMR (377 MHz, d_6_-DMSO): δ (ppm) −137.73 (m, 1F), −141.08 (m, 1F); HRMS (ESI-Q-TOF): *m*/*z* [M+H-Benzophenone]^+^ calcd. for C_22_H_16_Cl_1_F_2_N_3_O_2_S_1_: 441.1191; found: 441.1142.

**4-Amino-2-(2,3-difluorobenzylmercapto)-pyrido[3,4-*d*]pyrimidine-4-one (20)**

In a screw-capped reaction tube equipped with a stir bar, 2-(2,3-difluorobenzylmercapto)-4-(4-methoxybenzyl)-(*N*-diphenylmethylene)-pyrido[3,4-*d*]pyrimidine-6-amine **19** (324 mg, 0.536 mmol) and TFA (6.0 mL) were combined and heated to 75 °C for 1 h. Next, the reaction mixture was concentrated in vacuo. Ethyl acetate and saturated aqueous NaHCO_3_ were added; the mixture was stirred and filtered. The obtained solid was washed with water and methanol, and dried under vacuum to afford impure 6-amino-2-(2,3-difluorobenzylmercapto)-pyrido[3,4-*d*]pyrimidine-4-one (112 mg, 0.350 mmol, 65%), which was used without further purification. mp > 300 °C.

**(2*R*)-2-[(6-Amino-2-(2,3-difluorobenzylmercapto)-pyrido[3,4-*d*]pyrimidine-4-yl)amino]propanol (21)**

The crude 6-amino-2-(2,3-difluorobenzylmercapto)-pyrido[3,4-*d*]pyrimidine-4-one **20** (100 mg, 0.312 mmol, 1.00 eq.), BOP (180 mg, 0.406 mmol, 1.30 eq.) and dry DMF (2.0 mL) were combined. DBU (0.07 mL, 0.5 mmol, 1.5 eq.) was added dropwise, and the reaction mixture was stirred for 10 min at room temperature. Next, (*R*)-alaninol (0.05 mL, 0.6 mmol, 2 eq.) was added dropwise. The reaction mixture was stirred for 23 h at room temperature and for 2 h at 60 °C. After concentrating in vacuo, EtOAc (40 mL) and saturated NH_4_Cl (10 mL) were added and the mixture was transferred to a separatory funnel. After phase separation, the aqueous phase was extracted with EtOAc (3 × 20 mL). The combined organic phases were washed with saturated NH_4_Cl (3 × 10 mL), water (2 × 10 mL) and brine (2 × 10 mL), dried over Na_2_SO_4_ and coated on Celite. Flash chromatography using 0–10% MeOH/DCM afforded the title compound (62 mg, 0.16 mmol, 53%) as an off-white solid. mp 174–176 °C.

^1^H NMR (400 MHz, d_6_-DMSO): δ (ppm) 8.47 (s, 1H), 7.84 (d, *J* = 7.9 Hz, 1H), 7.46–7.37 (m, 1H), 7.35–7.24 (m, 1H), 7.19–7.08 (m, 1H), 6.94 (s, 1H), 5.98 (s, 2H), 4.76 (t, *J* = 5.7 Hz, 1H), 4.42 (s, 2H), 4.37–4.27 (m, 1H), 3.56–3.47 (m, 1H), 3.46–3.37 (m, 1H), 1.16 (d, *J* = 6.7 Hz, 3H); ^13^C NMR (101 MHz, d_6_-DMSO): δ (ppm) 160.8, 156.9, 156.4, 149.6 (dd, *J* = 12.1, 245.7 Hz), 148.4, 148.1 (dd, *J* = 12.8, 246.5 Hz), 137.2, 128.7 (d, *J* = 11.1 Hz), 126.3 (t, *J* = 2.8 Hz), 124.5 (dd, *J* = 4.6, 6.9 Hz), 120.5, 116.0 (d, *J* = 16.9 Hz), 94.6, 63.7, 48.3, 27.0, 16.6; HRMS (ESI-Q-TOF): *m*/*z* [M+H]^+^ calcd. for C_17_H_17_F_2_N_5_O_1_S_1_: 378.11945; found: 378.1200.

**2,4-Dichloro-pyrido[3,4-*d*]pyrimidine (22)**

This compound was prepared following a reported procedure [54].

A mixture of 3-aminoisonicotinic acid (10.00 g, 72.4 mmol, 1.00 eq.) and urea (21.74 g, 181.0 mmol) in a screw-capped sealed 80 mL reaction tube was heated to 210 °C for 1 h. After cooling to room temperature, a solution of NaOH (14.2 g, 360 mmol) in 60 mL water was added and the mixture was heated to 100 °C for 1 h. Next, the reaction mixture was cooled down to room temperature, the solid was filtered and washed with water. The obtained crude product was suspended in AcOH (60 mL) and heated to 100 °C for 1 h. After cooling to room temperature, the reaction mixture was filtered and the obtained solid was washed with a large amount of water, and a small amount of methanol and diethyl ether. After drying in vacuo (40 °C) pyrido[3,4-*d*]pyrimidine-2,4-dione (9.948 g, 61.0 mmol, 84%) was obtained as a white solid.

Pyrido[3,4-*d*]pyrimidine-2,4-dione was first finely ground with a pestle and mortar. To a flame dried two-necked 500 mL round bottom flask, equipped with an Ar-balloon, septum and stir bar, pyrido[3,4-*d*]pyrimidine-2,4-dione (9.800 g, 60.07 mmol, 1.00 eq.), dry toluene (100 mL) and POCl_3_ (55 mL, 92 mmol, 10 eq.) were added. While cooling to 0 °C in an ice bath, DIPEA (21.5 mL, 15.9 mmol, 123.1 eq.) was added dropwise. After stirring at 0 °C for 30 min, the reaction mixture was stirred at room temperature for 24 h and concentrated in vacuo. To the obtained oil, diethyl ether and ice water were added. The mixture was neutralized with saturated aqueous NaHCO_3_ (pH = 7) while cooling in an ice bath. Due to extensive tar formation, phase separation of the aqueous and organic layers did not occur. This was solved by pouring the mixture into a large beaker (2L) and adding a lot of paper towel to adsorb the tar. This mixture was extracted 5 times with 300 mL diethyl ether, by manually stirring the contents of the beaker with a large spatula and decanting off the organic phase. The combined organic phases were washed with water (2 × 50 mL) and brine (2 × 50 mL), decolorized with activated charcoal and dried over Na_2_SO_4_. After concentrating to dryness in vacuo, while keeping the heating bath at room temperature, and drying in vacuo (30 °C), the title compound (6.544 g, 32.72 mmol, 55%) was obtained as a beige-brown solid which was used without further purification.

**General procedure A: Nucleophilic aromatic substitution of 2,4-dichloro-pyrido[3,4-*d*]pyrimidine with amines**

To a flame dried, N_2_-flushed 8 mL reaction tube equipped with a stirring bar 2,4-dichloro-pyrido[3,4-*d*]pyrimidine **22** (200 mg, 1.00 mmol, 1.00 eq.) and dry ACN (4.0 mL) were added. While cooling to 0 °C in an ice bath, Et_3_N (0.21 mL, 1.5 mmol, 1.5 eq.) and an amine nucleophile (1.5 mmol, 1.5 eq.) were added dropwise. After stirring for 10 min at 0 °C, the reaction mixture was stirred at room temperature for the specified time. During and after the addition of Et_3_N and nucleophile, generally, the formation of a yellow precipitate occurred. Next, the reaction mixture was transferred to a 100 mL round bottom flask with methanol; the entire sample was dissolved under gentle heating, and coated on Celite. Flash chromatography using 0–20% MeOH/DCM afforded the title compounds as white or beige solids.

**General procedure B: Nucleophilic aromatic substitution of 2,4-dichloro-pyrido[3,4-*d*]pyrimidine with alcohols**

NaH (60% dispersion) was added to a solution of alcohol nucleophile (1.1 eq.) in dry acetonitrile (2.0 mL), in a flame dried N_2_-flushed 8 mL screw capped reaction tube, and the mixture was sonicated for 30 min. In a separate flame dried, N_2_-flushed 8 mL reaction tube equipped with a stir bar, 2,4-dichloro-pyrido[3,4-*d*]pyrimidine **22** (200 mg, 1.00 mmol, 1.00 eq.) and dry ACN (3.0 mL) were added. While cooling to 0 °C in an ice bath, the solution of alcohol sodium salt was added dropwise to the 2,4-dichloro-pyrido[3,4-*d*]pyrimidine solution. After stirring for 10 min at 0 °C, the reaction mixture was stirred at room temperature for the specified time. Next, the reaction mixture was transferred to a 100 mL round bottom flask with methanol; the entire sample was dissolved under gentle heating, and coated on Celite. Flash chromatography using 0–100% EtOAc/DCM, followed by trituration with pentane and drying under vacuum (40 °C), afforded the title compounds as white or beige solids.

**General procedure C: Nucleophilic aromatic substitution of 2-chloropyrido[3,4-*d*]pyrimidines with thiols**

To a flame dried, N_2_-flused 8 mL reaction tube equipped with a stir bar, the appropriate 2-chloropyrido[3,4-*d*]pyrimidine (0.25 mmol, 1.00 eq.), thiol nucleophile (0.50 mmol, 2.00 eq.) and dry 1,4-dioxane (1 mL) were combined. NaH 60% dispersion in mineral oil (20 mg, 0.50 mmol, 0.50 eq.) was added in one portion. After stirring at room temperature overnight, methanol (1.0 mL) was added and the mixture was heated to 60 °C for 30 min. After cooling to room temperature, the reaction mixture was transferred to a 100 mL round bottom flask with methanol; the entire sample was dissolved under gentle heating, and coated on Celite. Flash chromatography using 0–20 MeOH/DCM, followed by trituration with pentane and drying under vacuum (40 °C), afforded the title compounds as white solids.

The following compounds were made according to these procedures:

**(2*R*)-2-[(6-Chloro-2-pyrido[3,4-*d*]pyrimidine-4-yl)amino]butanol (23a)**

This compound was prepared following general procedure A. (*R*)-2-Aminobutan-1-ol (0.14 mL, 1.5 mmol, 1.5 eq.) was reacted for 3h at room temperature. Flash chromatography was performed using 0–20% MeOH/DCM to afford the title compound (206 mg, 0.815 mmol, 82%) as an off-white solid. mp> 300 °C.

^1^H NMR (400 MHz, d_6_-DMSO): δ (ppm) 8.98 (s, 1H), 8.72–8.55 (m, 2H), 8.29 (d, *J* = 5.4 Hz, 1H), 4.82 (t, *J* = 5.2 Hz, 1H), 4.35–4.19 (m, 1H), 3.65–3.50 (m, 2H), 1.83–1.68 (m, 1H), 1.67–1.51 (m, 1H), 0.90 (t, *J* = 7.3 Hz, 3H); ^13^C NMR (400 MHz, d_6_-DMSO): δ (ppm) 160.8, 158.3, 150.5, 145.1, 144.2, 118.0, 115.9, 62.1, 54.9, 23.2, 10.6; HRMS (ESI-Q-TOF): *m*/*z* [M+H]^+^ calcd. for C_11_H_13_Cl_1_N_4_O_1_: 253.0851; found: 253.0848.

**2-[(2-Chloro-pyrido[3,4-*d*]pyrimidine-4-yl)amino]-2-methyl-propan-1-ol (23b)**

This compound was prepared following general procedure A. 2-Amino-2-methylpropan-1-ol (0.14 mL, 1.5 mmol, 1.5 eq.) was reacted for 4h at room temperature. Flash chromatography was performed using 0–20% MeOH/DCM, followed by a second column using 0–10 MeOH/Et_2_O to afford the title compound (165 mg, 0.653 mmol, 65%) as an off-white solid. mp > 300 °C.

^1^H NMR (400 MHz, d_6_-DMSO): δ (ppm) 8.96 (s, 1H), 8.59 (d, *J* = 5.6 Hz, 1H), 8.41 (d, *J* = 5.6 Hz, 1H), 5.16 (br. s, 1H), 3.70 (s, 2H), 1.44 (s, 6H); ^13^C NMR (101 MHz, d_6_-DMSO): δ (ppm) 160.0, 157.2, 150.5, 144.9, 144.1, 118.5, 116.3, 65.8, 57.5, 23.1; HRMS (ESI-Q-TOF): *m*/*z* [M+H]^+^ calcd. for C_11_H_13_Cl_1_N_4_O_1_: 253.08505; found: 253.0854.

**3-[(2-Chloro-pyrido[3,4-*d*]pyrimidine-4-yl)amino]propan-1,2-diol (23c)**

This compound was prepared following general procedure A. 3-Aminopropan-1,2-diol (0.12 mL, 1.5 mmol, 1.5 eq.) was reacted for 1h30 at room temperature. Flash chromatography was performed using 0–20% MeOH/DCM to afford the title compound (214 mg, 0.840 mmol, 84%) as a beige solid. mp > 300 °C.

^1^H NMR (400 MHz, d_6_-DMSO): δ (ppm) 9.06 (t, *J* = 5.0 Hz, 1H), 8.98 (s, 1H), 8.61 (d, *J* = 5.5 Hz, 1H), 8.19 (d, *J* = 5.5 Hz, 1H), 4.96 (d, *J* = 4.9 Hz, 1H), 4.68 (t, *J* = 5.5 Hz, 1H), 3.90–3.75 (m, 1H), 3.65 (dt, *J* = 4.9, 13.2 Hz, 1H), 3.51–3.38 (m, 3H); ^13^C NMR (101 MHz, d_6_-DMSO): δ (ppm) 160.7, 158.2, 150.5, 144.9, 144.3, 118.1, 115.8, 69.1, 64.0, 44.8; HRMS (ESI-Q-TOF): *m*/*z* [M+H]^+^ calcd. for C_10_H_11_Cl_1_N_4_O_2_: 255.06432; found: 255.0643.

**2-[(2-Chloro-pyrido[3,4-*d*]pyrimidine-4-yl)amino]ethanol (23d)**

This compound was prepared following general procedure A. Aminoethanol (0.1 mL, 1.7 mmol, 1.7 eq.) was reacted for 2h at room temperature. Flash chromatography was performed using 0–20% MeOH/DCM followed by a second manual column using 0–10% MeOH/Et_2_O to afford the title compound (131 mg, 0.583 mmol, 58%) as an off-white solid. mp > 300 °C.

^1^H NMR (400 MHz, d_6_-DMSO): δ(ppm) 9.14 (s, 1H), 8.99 (d, *J* = 0.5 Hz, 1H), 8.63 (d, *J* = 5.6 Hz, 1H), 8.17 (dd, *J* = 0.7, 5.6 Hz, 1H), 4.87 (t, *J* = 5.4 Hz, 1H), 3.69–3.55 (m, 2H); ^13^C NMR (400 MHz, d_6_-DMSO): δ(ppm) 160.6, 158.2, 150.5, 144.9, 118.1, 115.7, 58.5, 43.8; HRMS (ESI-Q-TOF): *m*/*z* [M+H]^+^ calcd. for C_9_H_9_Cl_1_N_4_O_1_: 225.05376; found: 225.0539.

**3-[(2-(2,3-Difluorobenzylmercapto)-pyrido[3,4-*d*]pyrimidine-4-yl)oxy]ethanol (23e)**

This compound was prepared following general procedure B. 3-[(2-Chloro-pyrido[3,4-*d*]pyrimidine-4-yl)amino]ethanol (56 mg, 0.25 mmol, 1.00 eq.) and 2,3-difluorobenzylthiol (80 mg mL, 0.50 mmol, 2.00 eq.) were used and reacted at 60 °C for 30 min. Flash chromatography was performed using 0–10% MeOH/DCM to afford the title compound (77 mg, 0.22 mmol, 88%) as a beige-brown solid. mp 181–183 °C.

^1^H NMR (400 MHz, DMSO): δ(ppm) 8.94 (s, 1H), 8.76 (t, *J* = 4.9 Hz, 1H), 8.50 (d, *J* = 5.5 Hz, 1H), 8.07 (d, *J* = 5.2 Hz, 1H), 7.47–7.38 (m, 1H), 7.35–7.23 (m, 1H), 7.18–7.08 (m, 1H), 4.83 (t, *J* = 5.3 Hz, 1H), 4.49 (s, 2H), 3.66–3.52 (m, 2H); ^13^C NMR (101 MHz, DMSO): δ(ppm) 163.4, 158.2, 150.1, 149.6 (dd, *J* = 12.6, 245.3 Hz), 148.2 (dd, *J* = 12.8, 246.8 Hz), 144.3, 143.0, 128.3 (d, *J* = 11.4 Hz), 126.3 (t, *J* = 11.4 Hz), 126.3 (t, *J* = 2.8 Hz), 124.6 (dd, *J* = 4.7, 6.9 Hz), 117.3, 116.3 (d, *J* = 16.9 Hz), 115.6, 58.7, 43.5, 27.2; ^19^F NMR (377 MHz, DMSO): δ(ppm) −139.35 (m, 1F), −148.50 (m, 1F) HRMS (ESI-Q-TOF): *m*/*z* [M+H]^+^ calcd. for C_16_H_14_F_2_N_4_O_1_S_1_: 349.09290; found: 349.0922.

**(2*R*)-2-[(2-(2,3-Difluorobenzylmercapto)-pyrido[3,4-*d*]pyrimidine-4-yl)amino]butanol (24a)**

This compound was prepared following general procedure C. (2*R*)-2-[(6-Chloro-2-pyrido[3,4-*d*]pyrimidine-4-yl)amino]butanol (63 mg, 0.25 mmol, 1.00 eq.) and 2,3-difluorobenzylthiol (80 mg mL, 0.50 mmol, 2.00 eq.) were used and reacted at 60 °C for 30 min. Flash chromatography was performed using 0–10% MeOH/DCM to afford the title compound (87 mg, 0.23 mmol, 92%) as a white solid. mp 174–176 °C.

^1^H NMR (400 MHz, d_6_-DMSO): δ (ppm) 8.93 (s, 1H), 8.50 (d, *J* = 5.5 Hz, 1H), 8.25 (d, *J* = 8.1 Hz, 1H), 8.19 (d, *J* = 5.5 Hz, 1H), 7.51–7.37 (m, 1H), 7.36–7.23 (m, 1H), 7.20–7.07 (m, 1H), 7.77 (t, *J* = 5.4 Hz, 1H), 4.49 (d, *J* = 1.9 Hz, 2H), 4.37–4.17 (m, 1H), 3.59–3.43 (m, 2H), 1.79–1.65 (m, 1H), 1.64–1.49 (m, 1H), 0.86 (t, *J* = 7.4 Hz, 3H); ^13^C NMR (101 MHz, d_6_-DMSO): δ (ppm) 167.4, 158.3, 150.1, 149.6 (dd, *J* = 12.5, 245.4 Hz), 148.1 (dd, *J* = 12.9, 246.6 Hz), 144.4, 142.8, 128.3 (d, *J* = 11.1 Hz), 126.3 (t, *J* = 2.7 Hz), 124.6 (dd, *J* = 4.7, 6.9 Hz), 117.3, 116.1 (d, *J* = 16.9 Hz), 115.8, 62.2, 54.4, 27.2, 23.3, 10.6; ^19^F NMR (377 MHz, d_6_-DMSO): δ (ppm) −139.39 (m, 1F), −142.85 (m, 1F); HRMS (ESI-Q-TOF): *m*/*z* [M+H]^+^ calcd. for C_18_H_18_F_2_N_4_O_1_S_1_: 377.12420; found: 377.1239.

**2-[(2-(2,3-Difluorobenzylmercapto)-pyrido[3,4-*d*]pyrimidine-4-yl)amino]-2-methyl-propan-1-ol (24b)**

This compound was prepared following general procedure C. 2-[(2-Chloro-pyrido[3,4-*d*]pyrimidine-4-yl)amino]-2-methyl-propan-1-ol (63 mg, 0.25 mmol, 1.00 eq.) and 2,3-difluorobenzylthiol (80 mg mL, 0.50 mmol, 2.00 eq.) were used and reacted at 60 °C for 30 min. Flash chromatography was performed using 0–10% MeOH/DCM to afford the title compound (93 mg, 0.247 mmol, 99%) as a white solid. mp 166–168 °C.

^1^H NMR (400 MHz, d_6_-DMSO): δ (ppm) 8.94 (s, 1H), 8.49 (d, *J* = 5.5 Hz, 1H), 8.23 (d, *J* = 5.5 Hz, 1H), 7.49 (s, 1H), 7.47–7.40 (m, 1H), 7.35- 7.25 (m, 1H), 7.20–7.10 (m, 1H), 4.88 (t, *J* = 5.9 Hz, 1H), 4.49 (s, 2H), 3.66 (d, *J* = 5.8 Hz, 2H), 1.40 (s, 6H); ^13^C NMR (101 MHz, d_6_-DMSO): δ (ppm) 166.7, 157.7, 150.1, 149.6 (dd, *J* = 12.6, 245.4 Hz), 148.2 (dd, *J* = 12.8, 246.7 Hz), 144.2, 142.9, 127.9 (d, *J* = 11.2 Hz), 126.3 (t, *J* = 2.9 Hz), 124.6 (dd, *J* = 4.7, 6.9 Hz), 117.7, 116.2 (d, *J* = 16.9 Hz), 116.0, 65.9, 56.9, 27.2, 23.3; ^19^F NMR (377 MHz, d_6_-DMSO): δ (ppm) −139.33 (m, 1F), −142.78 (m, 1F); HRMS (ESI-Q-TOF): *m*/*z* [M+H]^+^ calcd. for C_18_H_18_F_2_N_4_O_1_S_1_: 377.12420; found: 377.1238.

**3-[(2-(2,3-Difluorobenzylmercapto)-pyrido[3,4-*d*]pyrimidine-4-yl)amino]propan-1,2-diol (24c)**

This compound was prepared following general procedure C. 3-[(2-Chloro-pyrido[3,4-*d*]pyrimidine-4-yl)amino]propan-1,2-diol (64 mg, 0.25 mmol, 1.00 eq.) and 2,3-difluorobenzylthiol (80 mg mL, 0.50 mmol, 2.00 eq.) were used and reacted at 60 °C for 30 min. Flash chromatography was performed using 0–10% MeOH/DCM to afford the title compound (84 mg, 0.22 mmol, 88%) as an off-white solid. mp = 156–158 °C.

^1^H NMR (400 MHz, d_6_-DMSO): δ (ppm) 8.94 (s, 1H), 8.72 (t, *J* = 5.5 Hz, 1H), 8.50 (d, *J* = 5.4 Hz, 1H), 8.11 (d, *J* = 5.4 Hz, 1H), 7.50–7.37 (app. t, *J* = 6.7 Hz, 1H), 7.36–7.23 (m, 1H), 7.19–7.07 (m, 1H), 4.92 (d, *J* = 4.7 Hz, 1H), 4.65 (t, *J* = 5.4 Hz, 1H), 4.50 (s, 2H), 3.88–3.75 (m, 1H), 3.74–3.61 (m, 2H), 3.45–3.37 (m, 2H); ^13^C NMR (101 MHz, d_6_-DMSO): δ (ppm) 167.3, 158.2, 150.1, 149.6 (dd, *J* = 12.5, 245.2 Hz), 148.2 (dd, *J* = 12.8, 246.8 Hz), 144.3, 143.0, 128.3 (d, *J* = 11.2 Hz), 126.3 (t, *J* = 2.6 Hz), 124.6 (dd, *J* = 4.7, 7.0 Hz), 117.3, 116.2 (d, *J* = 16.9 Hz), 115.7, 69.3, 64.0, 44.5, 27.3; ^19^F NMR (377 MHz, d_6_-DMSO): δ (ppm) −139.31 (m, 1F), −142.70 (m, 1F); HRMS (ESI-Q-TOF): *m*/*z* [M+H]^+^ calcd. for C_17_H_16_F_2_N_4_O_2_S_1_: 379.10347; found: 379.1031.

**3-[(2-(2,3-Difluorobenzylmercapto)-pyrido[3,4-*d*]pyrimidine-4-yl)amino]ethanol (24d)**

This compound was prepared following general procedure C. 3-[(2-Chloro-pyrido[3,4-*d*]pyrimidine-4-yl)amino]ethanol (56 mg, 0.25 mmol, 1.00 eq.) and 2,3-difluorobenzylthiol (80 mg mL, 0.50 mmol, 2.00 eq.) were used and reacted at 60 °C for 30 min. Flash chromatography was performed using 0–10% MeOH/DCM to afford the title compound (77 mg, 0.22 mmol, 88%) as a white solid. mp 181–183 °C.

^1^H NMR (400 MHz, d_6_-DMSO): δ (ppm) 8.94 (s, 1H), 8.76 (t, *J* = 4.9 Hz, 1H), 8.50 (d, *J* = 5.5 Hz, 1H), 8.07 (d, *J* = 5.2 Hz, 1H), 7.47–7.38 (m, 1H), 7.35–7.23 (m, 1H), 7.18–7.08 (m, 1H), 4.83 (t, *J* = 5.3 Hz, 1H), 4.49 (s, 2H), 3.66–3.52 (m, 2H); ^13^C NMR (101 MHz, d_6_-DMSO): δ (ppm) 163.4, 158.2, 150.1, 149.6 (dd, *J* = 12.6, 245.3 Hz), 148.2 (dd, *J* = 12.8, 246.8 Hz), 144.3, 143.0, 128.3 (d, *J* = 11.4 Hz), 126.3 (t, *J* = 11.4 Hz), 126.3 (t, *J* = 2.8 Hz), 124.6 (dd, *J* = 4.7, 6.9 Hz), 117.3, 116.3 (d, *J* = 16.9 Hz), 115.6, 58.7, 43.5, 27.2; ^19^F NMR (377 MHz, d_6_-DMSO): δ (ppm) −139.35 (m, 1F), −148.50 (m, 1F); HRMS (ESI-Q-TOF): *m*/*z* [M+H]^+^ calcd. for C_16_H_14_F_2_N_4_O_1_S_1_: 349.09290; found: 349.0922.

**3-[(2-(2,3-Difluorobenzylmercapto)-pyrido[3,4-*d*]pyrimidine-4-yl)oxy]ethanol (24e)**

This compound was prepared following general procedure B. 3-[(2-Chloro-pyrido[3,4-*d*]pyrimidine-4-yl)amino]ethanol (56 mg, 0.25 mmol, 1.00 eq.) and 2,3-difluorobenzylthiol (80 mg mL, 0.50 mmol, 2.00 eq.) were used and reacted at 60 °C for 30 min. Flash chromatography was performed using 0–10% MeOH/DCM to afford the title compound (77 mg, 0.22 mmol, 88%) as a beige-brown solid. mp 181–183 °C.

^1^H NMR (400 MHz, d_6_-DMSO): δ (ppm) 8.94 (s, 1H), 8.76 (t, *J* = 4.9 Hz, 1H), 8.50 (d, *J* = 5.5 Hz, 1H), 8.07 (d, *J* = 5.2 Hz, 1H), 7.47–7.38 (m, 1H), 7.35–7.23 (m, 1H), 7.18–7.08 (m, 1H), 4.83 (t, *J* = 5.3 Hz, 1H), 4.49 (s, 2H), 3.66–3.52 (m, 2H); ^13^C NMR (101 MHz, d_6_-DMSO): δ (ppm) 163.4, 158.2, 150.1, 149.6 (dd, *J* = 12.6, 245.3 Hz), 148.2 (dd, *J* = 12.8, 246.8 Hz), 144.3, 143.0, 128.3 (d, *J* = 11.4 Hz), 126.3 (t, *J* = 11.4 Hz), 126.3 (t, *J* = 2.8 Hz), 124.6 (dd, *J* = 4.7, 6.9 Hz), 117.3, 116.3 (d, *J* = 16.9 Hz), 115.6, 58.7, 43.5, 27.2; ^19^F NMR (377 MHz, d_6_-DMSO): δ (ppm) −139.35 (m, 1F), −148.50 (m, 1F); HRMS (ESI-Q-TOF): *m*/*z* [M+H]^+^ calcd. for C_16_H_14_F_2_N_4_O_1_S_1_: 349.09290; found: 349.0922.

**(2*R*)-2-[(6-Chloro-2-pyrido[3,4-*d*]pyrimidine-4-yl)amino]propanol (25)**

This compound was prepared following general procedure A. 2,4-Dichloropyrido[3,4-*d*]pyrimidine (1 g, 5.00 mmol, 1.00 eq.) and (*R*)-alaninol (0.59 mL, 7.6 mmol, 1.5 eq.) was reacted for 3h at room temperature. Flash chromatography was performed using 0–20% MeOH/DCM, followed by trituration with pentane afforded the title compound (970 mg, 4.06 mmol, 81%) as an off-white solid. mp > 300 °C.

^1^H NMR (400 MHz, d_6_-DMSO): δ (ppm) 8.97 (s, 1H), 8.81 (d, *J* = 7.8 Hz, 1H), 8.61 (d, *J* = 5.6 Hz, 1H), 8.32 (d, *J* = 5.6 Hz, 1H), 4.91 (br. s, 1H), 4.47–4.30 (m, 1H), 3.61–3.46 (m, 2H), 1.23 (d, *J* = 6.7 Hz, 3H); ^13^C NMR (101 MHz, d_6_-DMSO): δ (ppm) 160.2 158.3, 150.5, 145.1, 144.2, 118.1, 116.0, 63.5, 49.1, 16.3; HRMS (ESI-Q-TOF): *m*/*z* [M+Na]^+^ calcd. for C_10_H_11_Cl_1_N_4_O_1_: 261.0514 found: 261.0512.

**(2*R*)-2-[(2-(2,3-Difluorobenzylamino)-pyrido[3,4-*d*]pyrimidine-4-yl)amino]propanol (26a)**

In a flame dried, N_2_-flushed 8 mL screw-capped reaction tube equipped with a stirring bar (2*R*)-2-[(6-chloro-2-pyrido[3,4-*d*]pyrimidine-4-yl)amino]propanol **25** (119 mg, 0.500 mmol, 1.00 eq.), 2,3-difluorobenzylamine (161 mg, 1.13 mmol, 2.25 eq.) and dry 1,4-dioxane (5 mL) were added. The mixture was stirred at 100 °C for 22 h. After cooling to room temperature, the reaction mixture was dissolved in methanol and coated on Celite. Flash chromatography using 0–20% MeOH/DCM, followed by trituration with pentane, afforded the title compound (135 mg, 0.391 mmol, 78%) as a white-yellow solid. mp 136–140 °C.

^1^H NMR (400 MHz, d_6_-DMSO): δ (ppm) 8.59 (s, 1H), 8.15 (d, *J* = 4.9 Hz, 1H), 7.93 (d, *J* = 5.4 Hz, 1H), 7.75 (br. s, 1H), 7.39 (br. s, 1H),7.31–7.17 (m, 2H), 7.16–7.08 (m, 2H), 4.74 (br. s, 1H), 4.62 (d, *J* = 6.0 Hz, 2H), 4.35 (br. s, 1H), 3.60–3.39 (m, 2H), 1.27–1.02 (m, 3H); ^13^C NMR (101 MHz, d_6_-DMSO): δ (ppm) 160.1, 15.9, 149.5 (dd, *J* = 12.7, 245.0 Hz), 148.9, 147.7 (dd, *J* = 12.6, 245.3 Hz), 146.8, 138.9, 130.3, 124.4 (dd, *J* = 4.5, 7.0 Hz), 115.6, 115.4 (d, *J* = 17.0 Hz), 63.8, 48.1, 37.6, 16.8; ^19^F NMR (377 MHz, d_6_-DMSO): δ (ppm) −140.15 (m, 1F), −145.26 (m, 1F); HRMS (ESI-Q-TOF): *m*/*z* [M+H]^+^ calcd. for C_17_H_17_F_2_N_5_O_1_: 346.1474; found: 346.145.

**(2*R*)-2-[(2-(2,3-Difluorobenzyloxy)-pyrido[3,4-*d*]pyrimidine-4-yl)amino]propanol (26b)**

In a flame dried, N_2_-flushed 8 mL screw-capped reaction tube equipped with a stir bar (2*R*)-2-[(6-chloro-2-pyrido[3,4-*d*]pyrimidine-4-yl)amino]propanol **25** (119 mg, 0.500 mmol, 1.00 eq.), 2,3-difluorobenzylalcohol (0.56 mL, 5.0 mmol, 10 eq.), dry 1,4-dioxane (4 mL) and K_2_CO_3_ (138 mg, 1.00 mmol, 2.00 eq.) were added. The mixture was stirred at 120 °C for 48 h. After cooling to room temperature, the reaction mixture was dissolved in methanol and coated on Celite. Flash chromatography using 0–10% MeOH/DCM, followed by trituration with pentane, afforded the title compound (87 mg, 0.25 mmol, 50%) as a light-brown solid. mp 167–169 °C.

^1^H NMR (400 MHz, d_6_-DMSO): δ (ppm) 8.88 (s, 1H), 8.43 (d, *J* = 5.2 Hz, 1H), 8.38 (d, *J* = 7.6 Hz, 1H), 8.15 (d, *J* = 5.3 Hz, 1H), 7.70–7.33 (m, 2H), 7.32–7.12 (m, 1H), 5.48 (s, 2H), 4.82 (t, *J* = 5.5 Hz, 1H), 4.47–4.26 (m, 2H), 3.62–3.50 (m, 1H), 3.50–3.40 (m, 1H), 1.21 (d, *J* = 6.6 Hz, 3H); ^13^C NMR (101 MHz, d_6_-DMSO): δ (ppm) 162.7, 160.9, 149.9, 149.6 (dd, *J* = 12.3, 245.4 Hz), 148.2 (dd, *J* = 12.9, 247.7 Hz), 145.6, 141.7, 126.7 (d, *J* = 11.4 Hz), 125.9 (t, *J* = 2.6 Hz), 124.9 (dd, *J* = 4.7, 6.7 Hz), 117.3 (d, *J* = 16.8 Hz), 116.8, 115.8, 63.7, 61.4, 48.7, 16.5; ^19^F NMR (377 MHz, d_6_-DMSO): δ (ppm) −139.34 (m, 1F), −143.8 (m, 1F); HRMS (ESI-Q-TOF): *m*/*z* [M+H]^+^ calcd. for C_17_H_16_F_2_N_4_O_2_: 347.1314; found: 347.1319.

**(2*R*)-2-[(2-(Phenylmercapto)-pyrido[3,4-*d*]pyrimidine-4-yl)amino]propanol (26c)**

This compound was prepared following general procedure C. (2*R*)-2-[(6-Chloro-2-pyrido[3,4-d]pyrimidine-4-yl)amino]propanol (60 mg, 0.25 mmol, 1.00 eq.) and thiophenol (55 mg, 0.50 mmol, 2.00 eq.) were used and reacted at 60 °C for 1 h 30 min. Flash chromatography was performed using 0–20% MeOH/DCM to afford the title compound (62 mg, 0.19 mmol, 76%) as an off-white solid. mp 193–196 °C.

^1^H NMR (400 MHz, d_6_-DMSO): δ (ppm) 8.79 (s, 1H), 8.47 (d, *J* = 5.5 Hz, 1H), 8.28 (d, *J* = 7.4 Hz, 1H), 8.13 (d, *J* = 5.5 Hz, 1H), 7.70–7.60 (m, 2H), 7.51–7.40 (m, 3H), 4.73 (t, *J* = 5.6 Hz, 1H), 4.12–3.96 (m, 1H), 3.45–3.37 (m, 2H), 1.08 (d, *J* = 6.7 Hz, 3H); ^13^C NMR (101 MHz, d_6_-DMSO): δ (ppm) 168.4, 157.8, 150.1, 144.7, 142.7, 135.0, 129.4, 128.94, 128.85, 117.3, 115.7, 63.3, 48.8, 16.3; HRMS (ESI-Q-TOF): *m*/*z* [M+H]^+^ calcd. for C_16_H_16_N_4_O_1_S_1_: 347.1314; found: 347.1319.

**(2*R*)-2-[(2-(Phenylethylmercapto)-pyrido[3,4-*d*]pyrimidine-4-yl)amino]propanol (26d)**

This compound was prepared following general procedure C. (2*R*)-2-[(6-Chloro-2-pyrido[3,4-d]pyrimidine-4-yl)amino]propanol (60 mg, 0.25 mmol, 1.00 eq.) and 2-phenylethanethiol (69 mg, 0.50 mmol, 2.00 eq.) were used and reacted at 60 °C for 1 h 30 min. Flash chromatography was performed using 0–20% MeOH/DCM, followed by trituration with pentane, to afford the title compound (76 mg, 0.223 mmol, 89%) as a white solid. mp 182–184 °C.

^1^H NMR (400 MHz, d_6_-DMSO): δ (ppm) 8.91 (s, 1H), 8.48 (d, *J* = 5.6 Hz, 1H), 8.26 (d, *J* = 7.8 Hz, 8.15 (d, *J* = 5.5 Hz, 1H), 7.44–7.26 (m, 4H), 7.26–7.17 (m, 1H), 4.83 (br. s, 1H), 4.50–4.35 (m, 1H), 3.62–3.44 (m, 2H), 3.42–3.35 (m, 2H), 3.01 (t, *J* = 7.7 Hz, 2H), 0.122 (d, *J* = 6.7 Hz, 3H); ^13^C NMR (101 MHz, d_6_-DMSO): δ (ppm) 168.5, 157.6, 150.1, 144.6, 142.6, 140.5, 128.6, 128.4, 126.2, 117.1, 115.7, 63.7, 48.6, 35.6, 31.5, 16.6; HRMS (ESI-Q-TOF): *m*/*z* [M+H]^+^ calcd. for C_18_H_20_N_4_O_1_S_1_: 341.1431; found: 341.1427.

**(2*R*)-2-[(2-(Hexylmercapto)-pyrido[3,4-*d*]pyrimidine-4-yl)amino]propanol (26e)**

This compound was prepared following general procedure C. (2*R*)-2-[(6-Chloro-2-pyrido[3,4-*d*]pyrimidine-4-yl)amino]propanol (60 mg, 0.25 mmol, 1.00 eq.) and hexane thiol (59 mg, 0.50 mmol, 2.00 eq.) were used and reacted at 60 °C for 30 min. Flash chromatography was performed using 0–20% MeOH/DCM to afford the title compound (62 mg, 0.19 mmol, 76%) as an off-white solid. mp 147–149 °C.

^1^H NMR (400 MHz, d_6_-DMSO): δ (ppm) 8.85 (s, 1H), 8.46 (d, *J* = 5.5 Hz, 1H), 8.23 (d, *J* = 7.8 Hz, 1H), 8.13 (d, *J* = 5.4 Hz, 1H), 4.81 (t, *J* = 5.7 Hz, 1H), 4.39 (septet, J = 6.6 Hz, 1H), 6.62–3.42 (m, 2H), 3.20–3.00 (m, 2H), 1.67 (quintet, *J* = 7.4 Hz, 2H), 1.48–1.35 (m, 2H), 1.34–1.24 (m, 4H), 1.23–1.17 (m, 3H), 0.85 (t, *J* = 7.0 Hz, 3H); ^13^C NMR (101 MHz, d_6_-DMSO): δ (ppm) 168.7, 157.5, 150.0, 144.6, 142.4, 117.0, 115.7, 63.6, 48.6, 30.8, 30.0, 29.4, 28.0, 22.0, 16.5, 13.8; HRMS (ESI-Q-TOF): *m*/*z* [M+H]^+^ calcd. for C_16_H_24_N_4_O_1_S_1_: 321.1744; found: 321.1747.

#### 3.2.2. CXCR2 Calcium Mobilization Assay

Cells stably expressing CXCR2 (U87.CXCR2) were cultivated in Dulbecco’s modified eagle medium (DMEM; Thermo Fisher Scientific, Waltham, MA, USA) supplemented with 10% Fetal Calf Serum (FCS, Thermo Fisher Scientific) and 10 µg/mL blasticidin (Invivogen, San Diego, CA, USA). Intracellular calcium fluxes induced in U87.CXCR2 cells upon human CXCL8 ligand stimulation were measured at 37 °C using the FLIPR Tetra system (Molecular Devices, Sunnyvale, CA, USA). Therefore, U87.CXCR2 cells were seeded at 2 × 10^4^ cells per well in gelatin-coated black-walled 96-well plates. This coating was done via a two hour incubation of 0.1% gelatin in Dulbecco’s phosphate-buffered saline (DPBS) on each well after which they were washed with DPBS. After cell-seeding, the cells were incubated overnight at 37 °C and 5% CO_2_. Then, growth medium was discarded, cells were loaded with 80 µL/well Calcium 6-dye mix (Molecular Devices, Sunnyvale, CA, USA) and incubated for two hours at 37 °C and 5% CO_2_. In the meantime, compounds were serially diluted in assay buffer (Hank’s Balanced Salt Solution (HBSS; Thermo Fisher Scientific), 20 mM HEPES buffer (Thermo Fischer Scientific), 0.5% FCS) in a 96-well plate. Moreover, a 96-well plate containing CXCL8 was prepared. After two hours of incubation, the cells were placed into the FLIPR Tetra system along with the compound plate and the plate containing CXCL8. Compounds were automatically added to the cell plate, followed by a ten minute incubation. Subsequently, CXCL8 was added at a final concentration of 15 nM. Changes in cytosolic calcium concentrations were measured in all 96 wells simultaneously in real time. For each compound, IC_50_ values for inhibition of the CXCL8-induced calcium response were determined based on negative and positive controls (i.e., untreated cells without or with CXCL8 stimulation, respectively).

## 4. Conclusions

**Figure 3:**
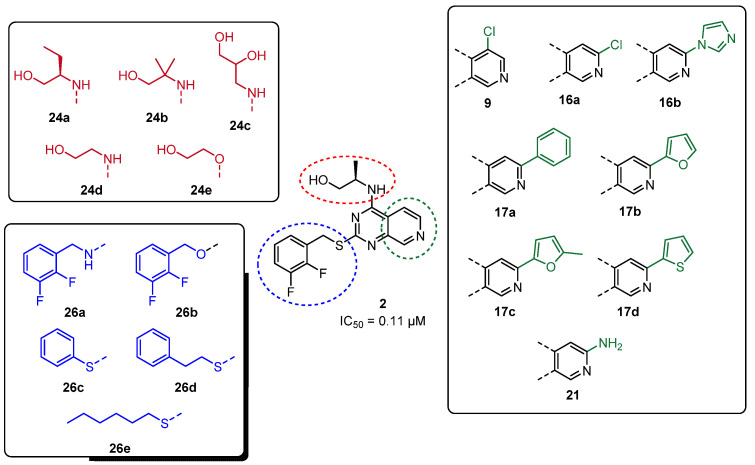
Summary of the SAR study. The compounds shown in red (**24a**–**e**) represent the 4-position variations, shown in blue (**26a**–**e**) the 2-positions variations, and those shown in in green (**9**, **16a**, **16b**, **17a**, **17b**, **17c** and **21**) the 5- and 6-positions variations.

We recently reported the discovery of a CXCR2 antagonist, based on a pyrido[3,4-*d*]pyrimidine skeleton. Since this scaffold has not been associated with CXCR2 antagonism before, we embarked on a hit-to-lead optimization campaign in order to optimize the potency of compound **2**. Using different synthetic approaches, various substituents were introduced at positions 2, 4, 5 and 6 of the pyrido[3,4-*d*]pyrimidine scaffold (Figure 3). Unfortunately, nearly all newly prepared analogues completely lacked CXCR2 antagonism, as evidenced by IC_50_ values exceeding 30 µM, the exception being a 6-furanyl-pyrido[3,4-*d*]pyrimidine analogue (compound **17b**) that is endowed with a five-fold reduced potency when compared to the original hit **2**. Therefore, we must conclude that pyrido[3,4-*d*]pyrimidine **2** is a ‘singleton’ hit compound.

## Figures and Tables

**Table 1:** CXCR2 antagonism of 5- and 6-substituted pyrido[3,4-*d*]pyrimidines.

Cmpd#		IC_50_ (µM) ^a^
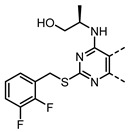		
Navarixin	see Figure 1	0.0049 ± 0.0033
thiazolo[4,5-*d*]pyrimidine	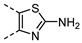	0.079 ± 0.058
**2**	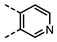	0.11 ± 0.019
**9**	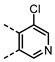	11.3
**16a**	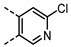	>30
**16b**	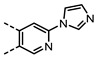	>30
**17a**	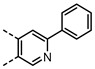	>30
**17b**	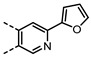	0.54 ± 0.26
**17c**	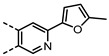	>30
**17d**	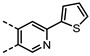	>30
**21**	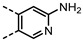	>30

^a^ IC_50_: the compound concentration that inhibits CXCL8 induced intracellular calcium flux by 50%. Data are expressed as average ± SD (in µM) of at least three experiments.

**Table 2:** CXCR2 antagonism of 4-substituted pyrido[3,4-*d*]pyrimidines.

Cmpd#	R^4^	IC_50_ (µM) ^a^
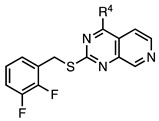		
Navarixin	Figure 1	0.0049 ± 0.0033
**2**	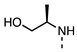	0.11 ± 0.019
**24a**	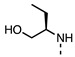	>30
**24b**	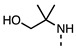	>30
**24c**	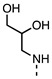	>30
**24d**	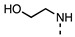	>30
**24e**	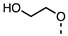	>30

^a^ IC_50_: the compound concentration that inhibits CXCL8 induced intracellular calcium flux by 50%. Data are expressed as average ± SD (in µM) of at least three experiments.

**Table 3:** CXCR2 antagonism of 2-substituted pyrido[3,4-*d*]pyrimidines.

Cmpd#	R^2^	IC_50_ (µM) ^a^
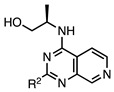		
Navarixin	See Figure 1	0.0049 ± 0.0033
**2**	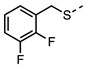	0.11 ± 0.019
**26a**	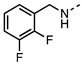	>30
**26b**	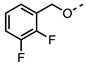	>30
**26c**	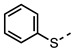	>30
**26d**	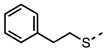	>30
**26e**	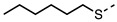	>30

^a^ IC_50_: the compound concentration that inhibits CXCL8 induced intracellular calcium flux by 50%. Data are expressed as average ± SD (in µM) of at least three experiments.

## Data Availability

The data presented in this study are available on request from the corresponding author.

## Supplementary Materials

The following supporting information can be downloaded at: https://www.mdpi.com/article/10.3390/molecules28052099/s1, NMR spectra.
