# Supplementary material for: Exploration of Pyrido[3,4-d]pyrimidines as Antagonists of the Human Chemokine Receptor CXCR2
Source: Molecules. 2023 Feb 23;28(5):2099. doi: 10.3390/molecules28052099 (PMC10004157; doi:10.3390/molecules28052099)
Supplement: Supplementary file 1 [file molecules-28-02099-s001.zip › molecules-2226887-supplementary.pdf]

Supplementary Material

Exploration of Pyrido[3,4-*d*]pyrimidines as Antagonists of the Human Chemokine Receptor CXCR2

Max Van Hoof [1], Sandra Claes [2], Katrijn Boon [2], Tom Van Loy [2], Dominique Schols [2], Wim Dehaen [1] and Steven De Jonghe [2,*]

[1] Molecular Design and Synthesis, Department of Chemistry, KU Leuven, Celestijnenlaan 200F, B-3001 Leuven, Belgium

[2] Department of Microbiology, Immunology and Transplantation, Rega Institute for Medical Research, Laboratory of Virology and Chemotherapy, KU Leuven, Herestraat 49, B-3000 Leuven, Belgium

NMR Spectra

3-Amino-5-chloropyridine-4-carbonitrile (5)

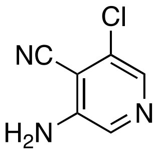

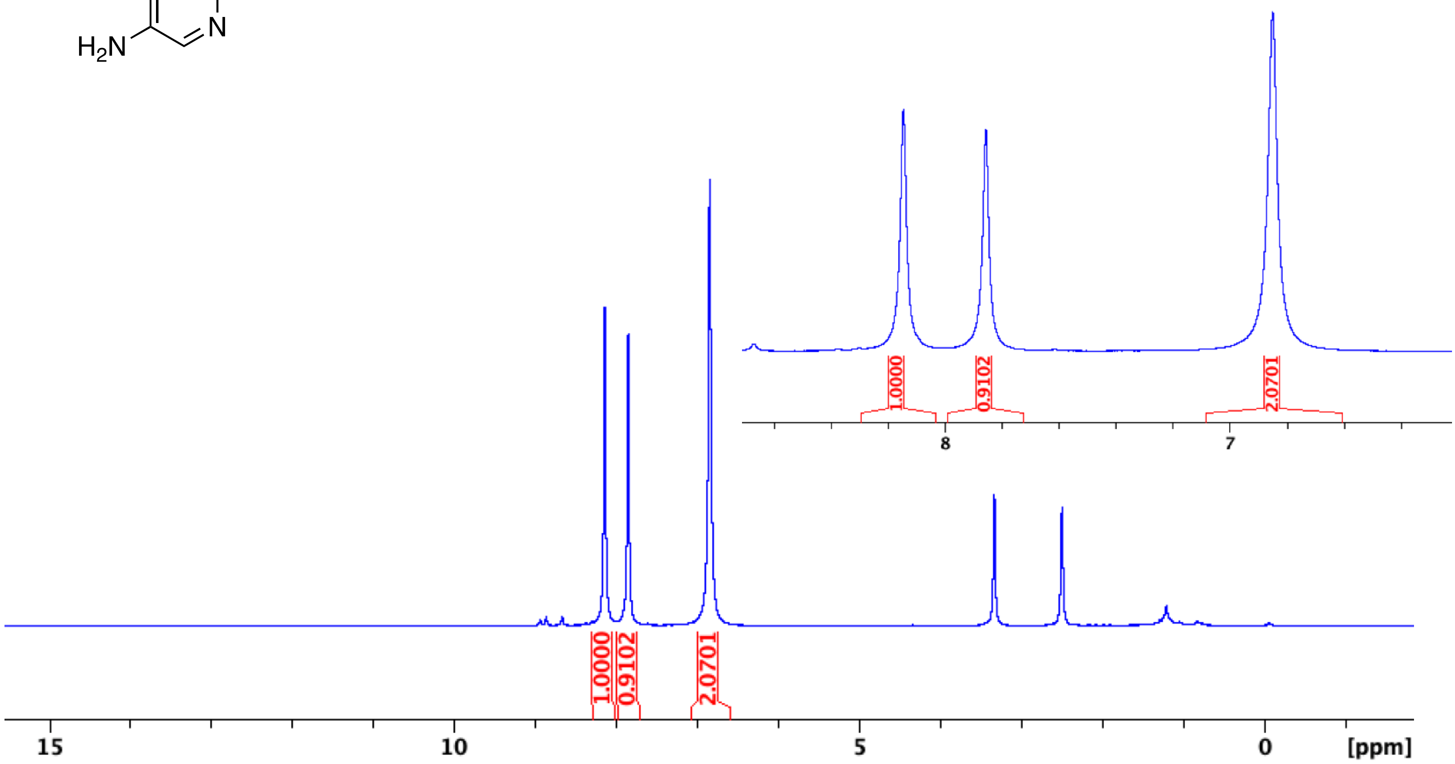

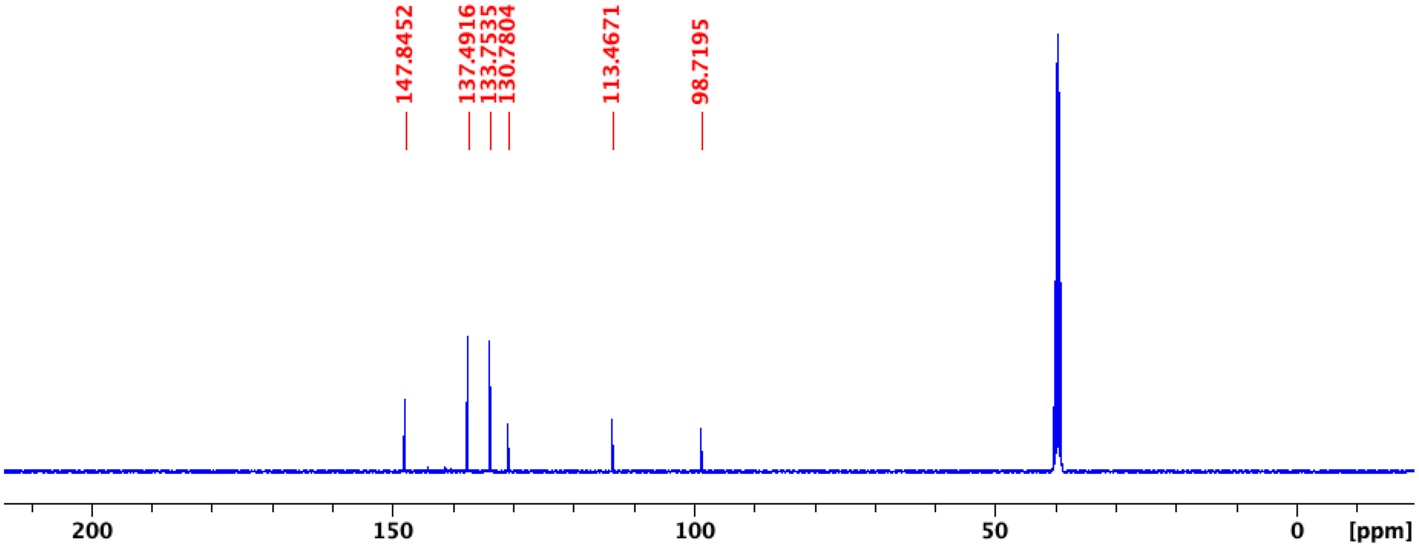

3-Amino-5-chloropyridine-4-carboxamide (6)

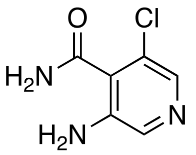

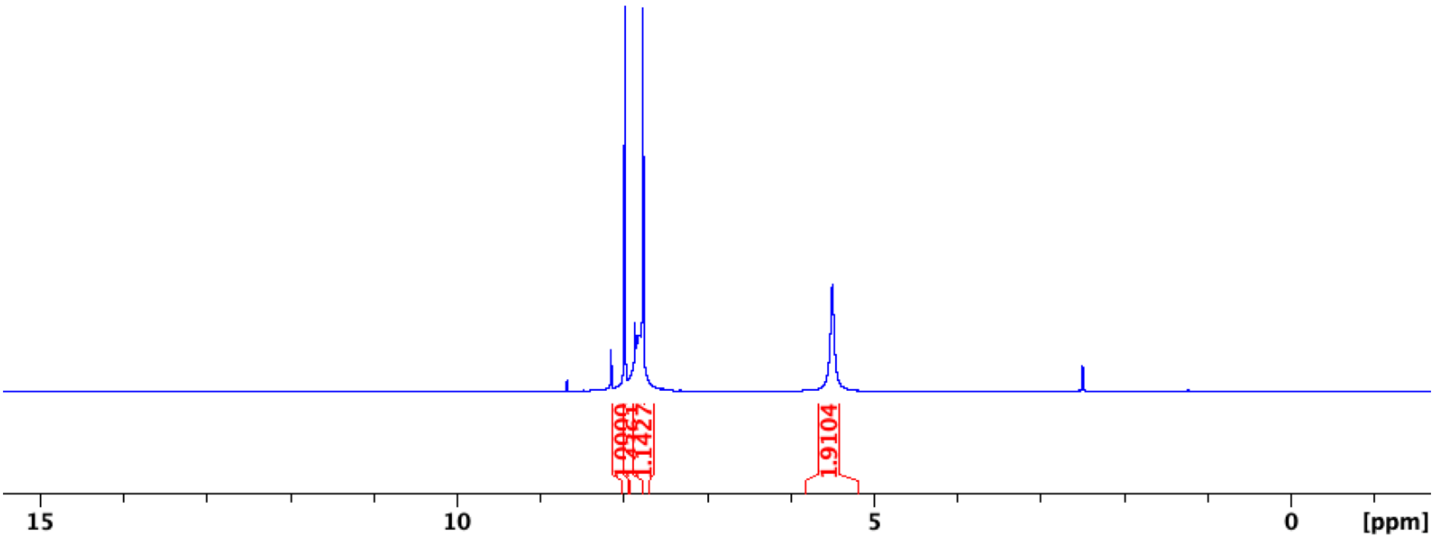

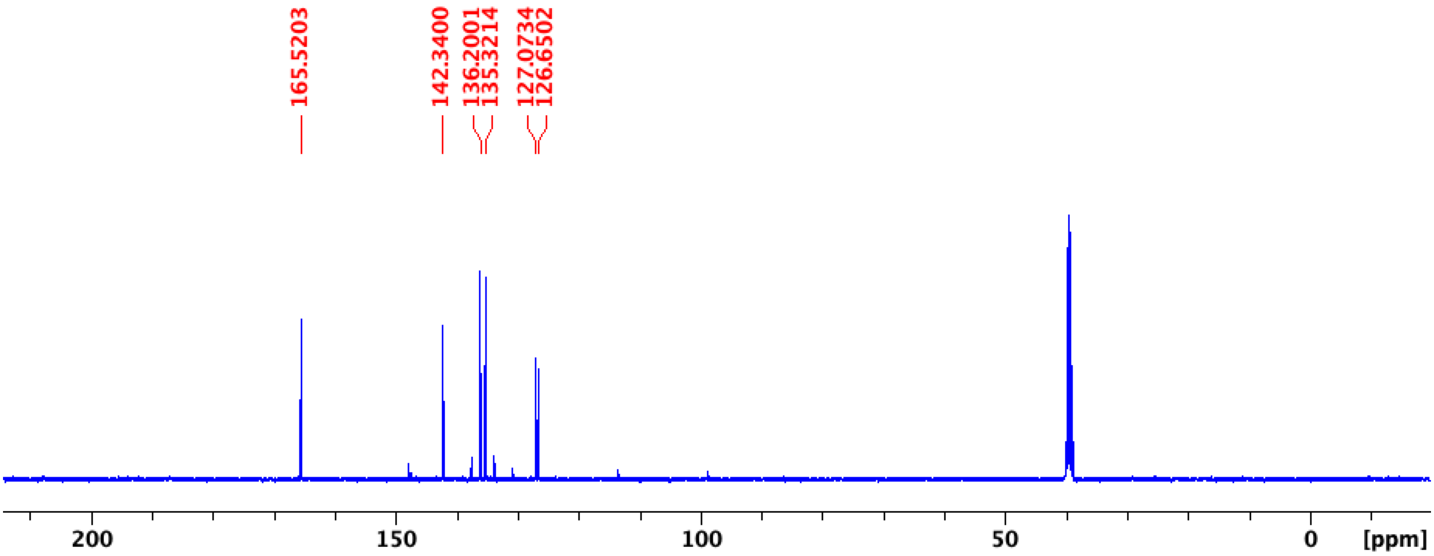

2-(2,3-Difluorobenzylmercapto)-5-chloropyrido[3,4-*d*]pyrimidine-4-one (8)

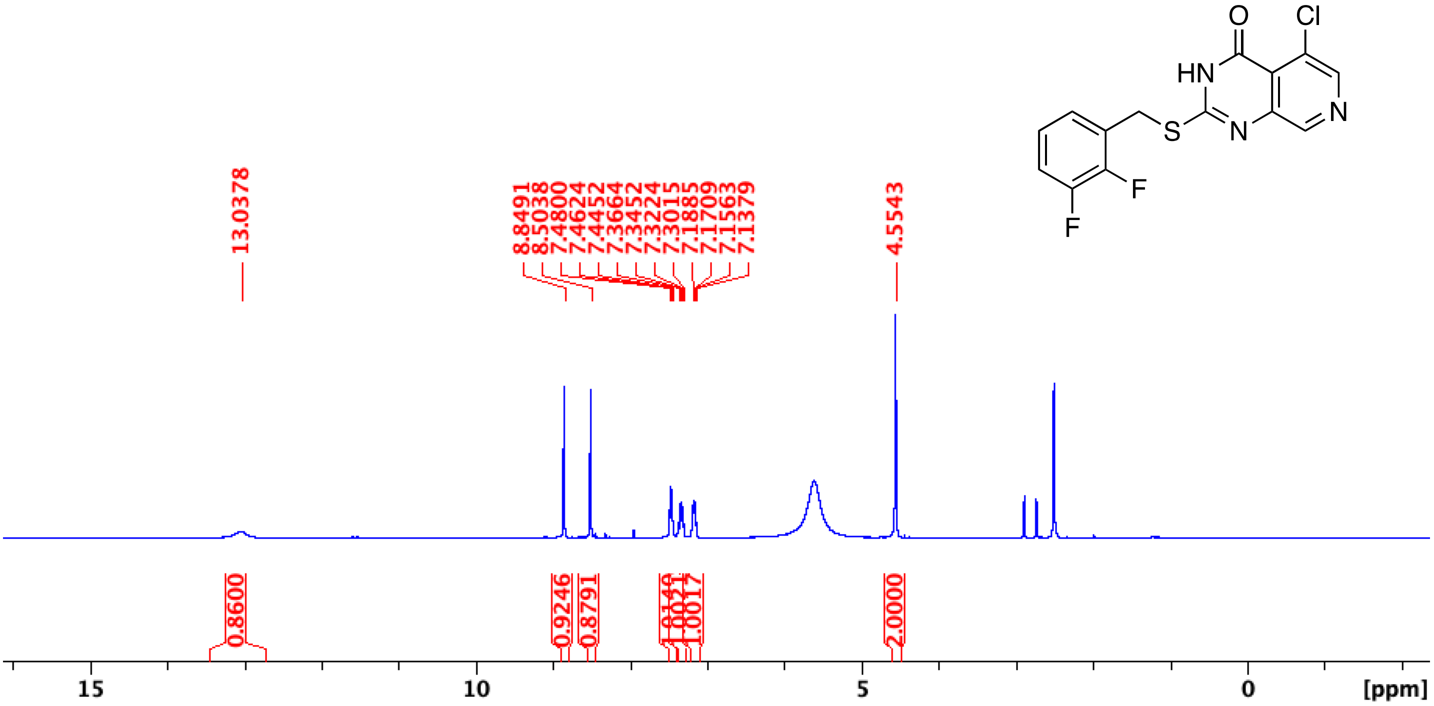

(2*R*)-2-[(5-Chloro-2-(2,3-difluorobenzylmercapto)-pyrido[3,4-*d*]pyrimidine-4-yl)amino]propanol (9)

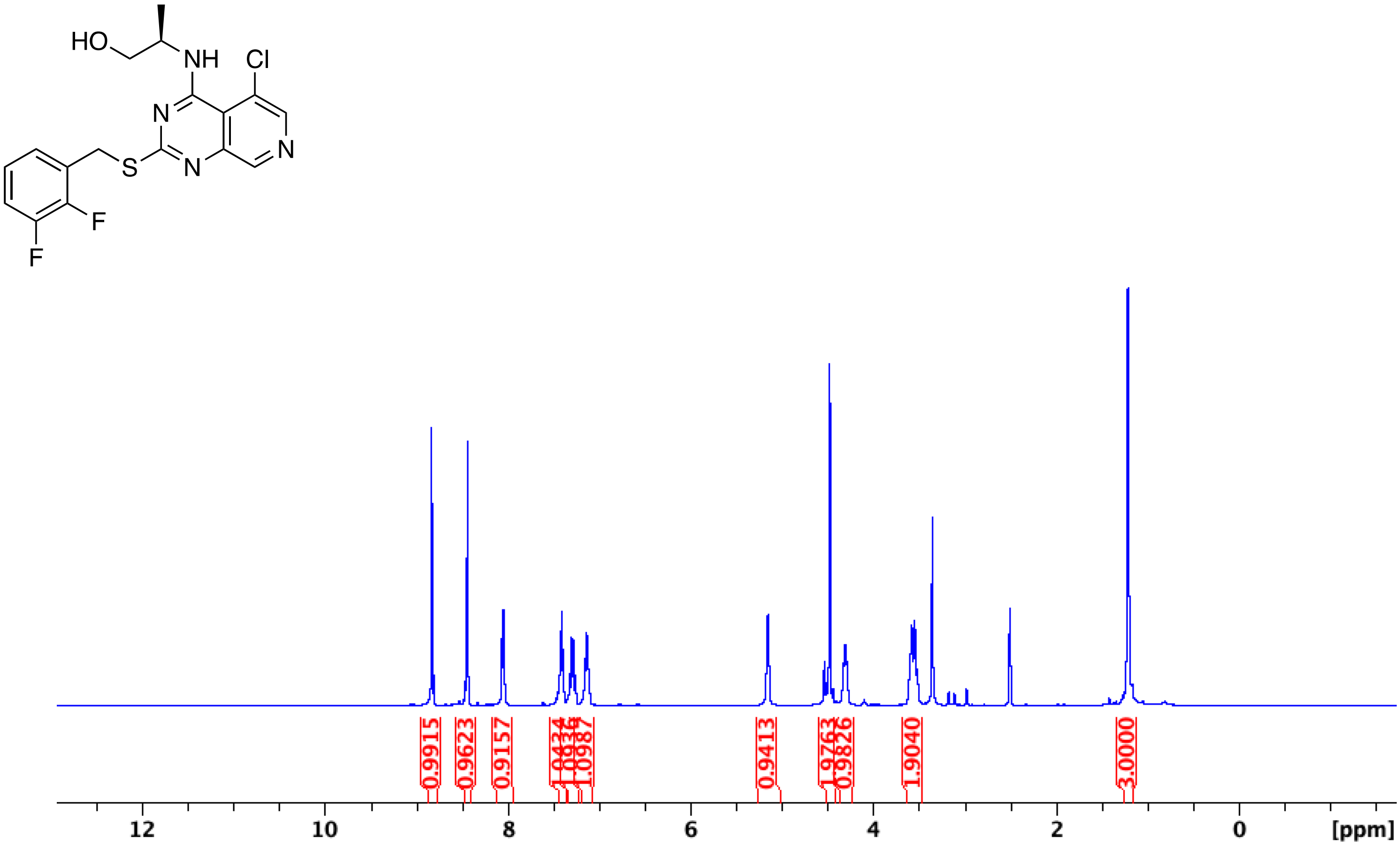

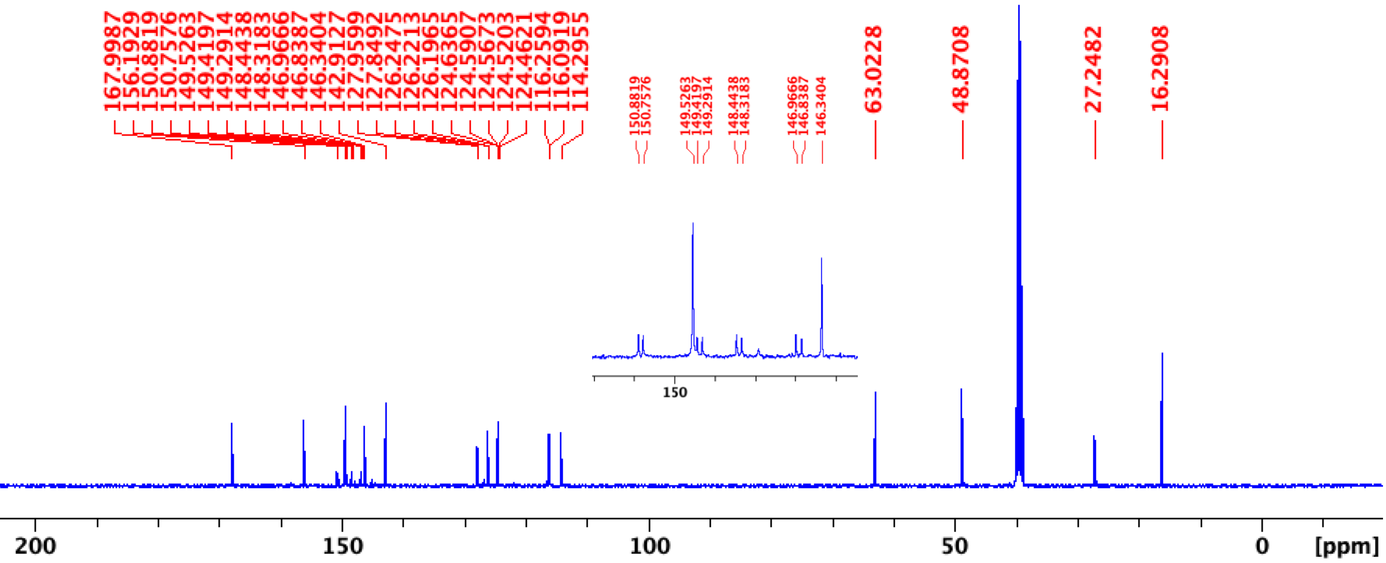
167.9987
156.1929
150.8819
150.7576
149.5263
149.4197
149.2914
148.4438
148.3183
146.9666
146.8387
146.3404
142.9127
127.9599
127.8492
126.2475
126.2213
126.1965
124.6365
124.5907
124.5673
124.5203
124.4621
116.2594
116.0919
114.2955
150.8819
150.7576
149.5263
149.4197
149.2914
148.4438
148.3183
146.9666
146.8387
146.3404
150
63.0228
48.8708
27.2482
16.2908
200
150
100
50
0
[ppm]

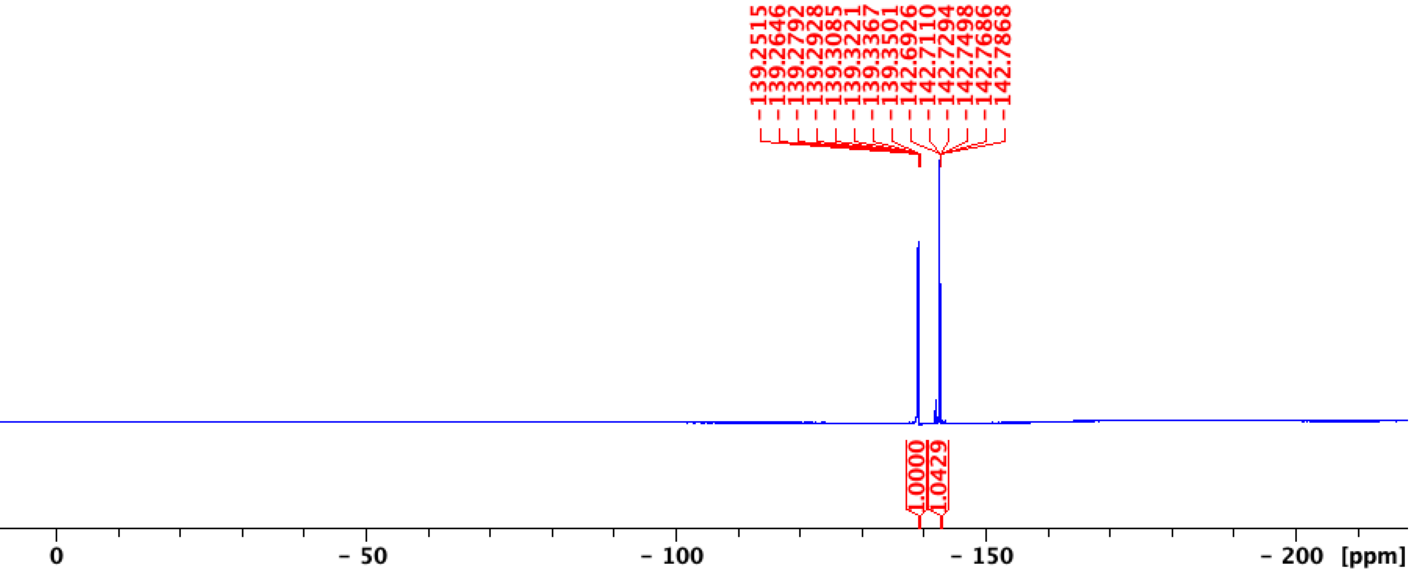
-139.2515
-139.2646
-139.2792
-139.2928
-139.3085
-139.3221
-139.3367
-139.3501
-142.6926
-142.7110
-142.7294
-142.7498
-142.7686
-142.7868
1.0000
1.0429
0
-50
-100
-150
-200
[ppm]

2-Bromo-5-nitropyridine-4-carboxylic acid (11)

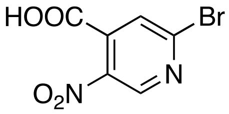

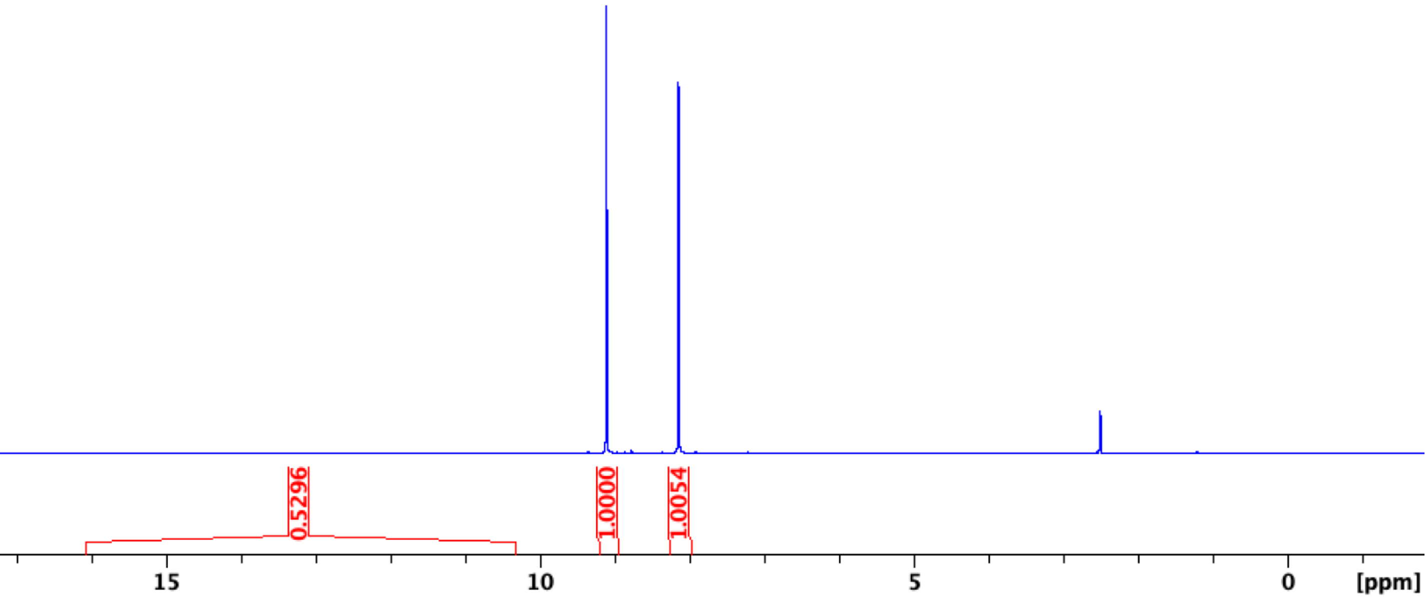

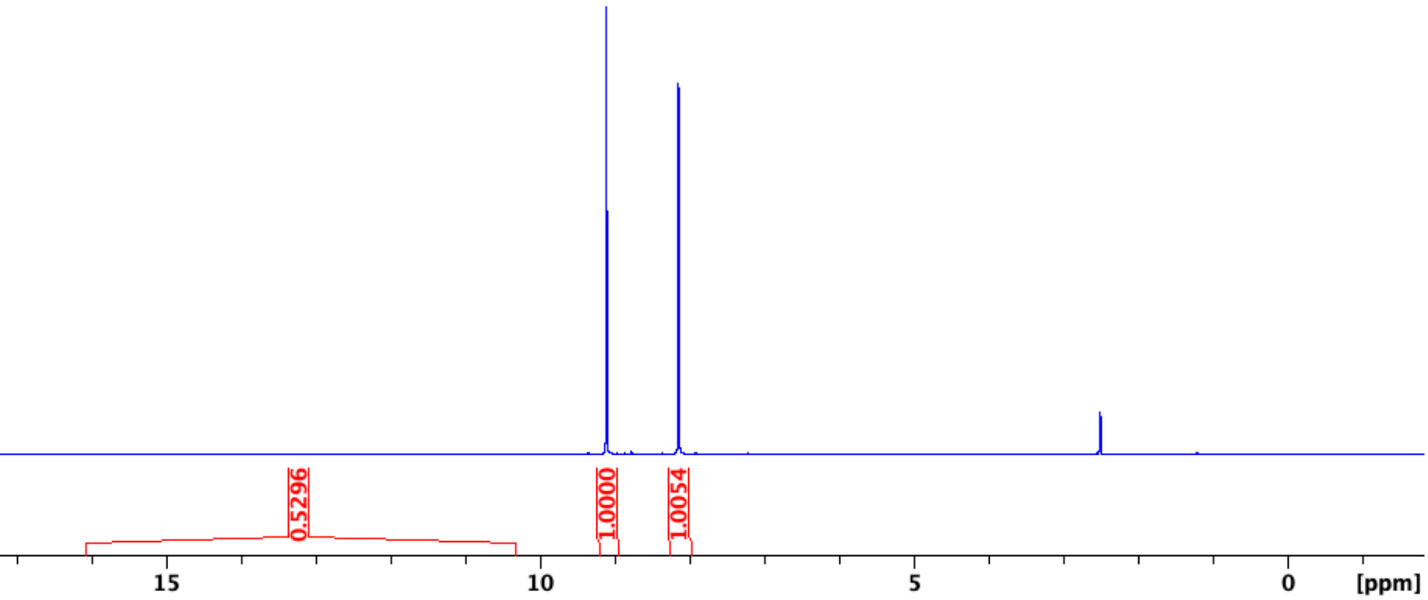

2-Imidazolyl-5-nitropyridine-4-carboxamide (12b)

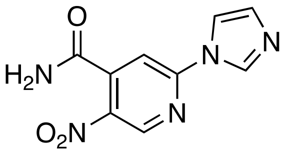

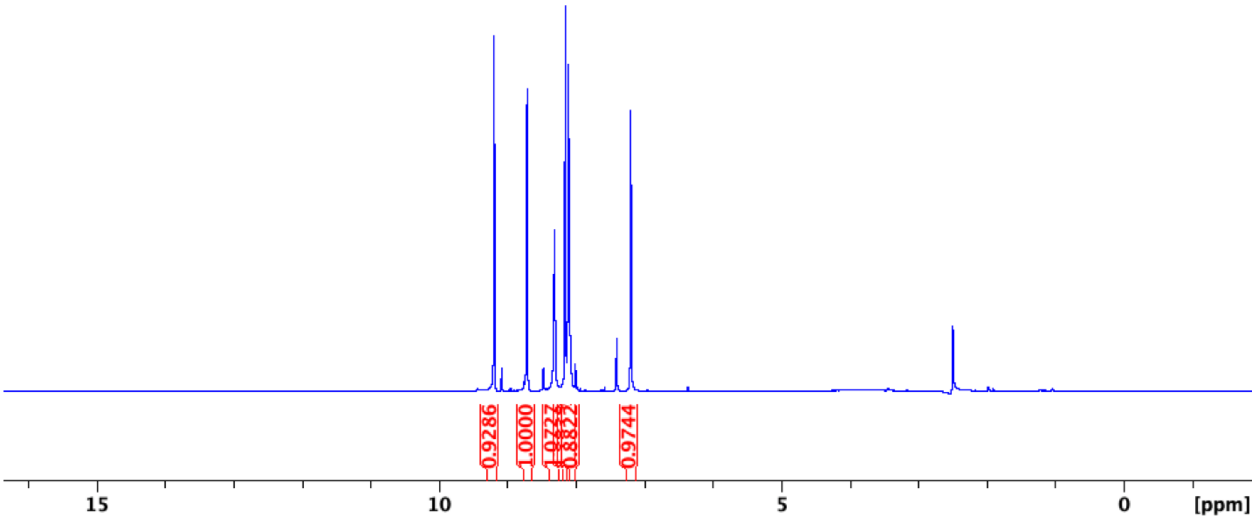

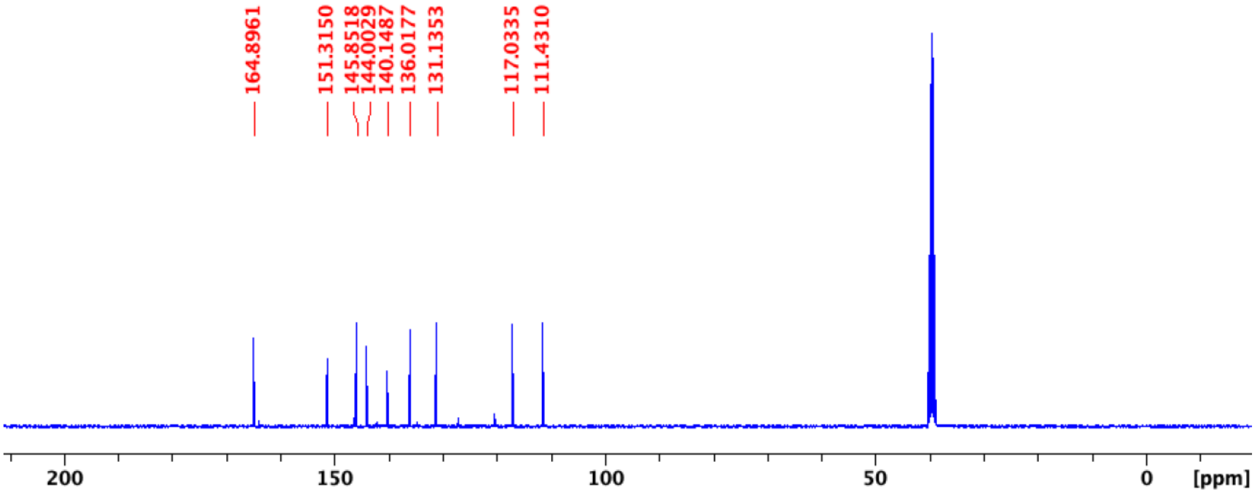

2-Chloro-5-aminopyridine-4-carboxamide (13a)

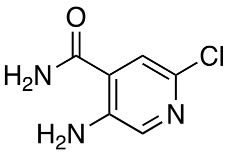

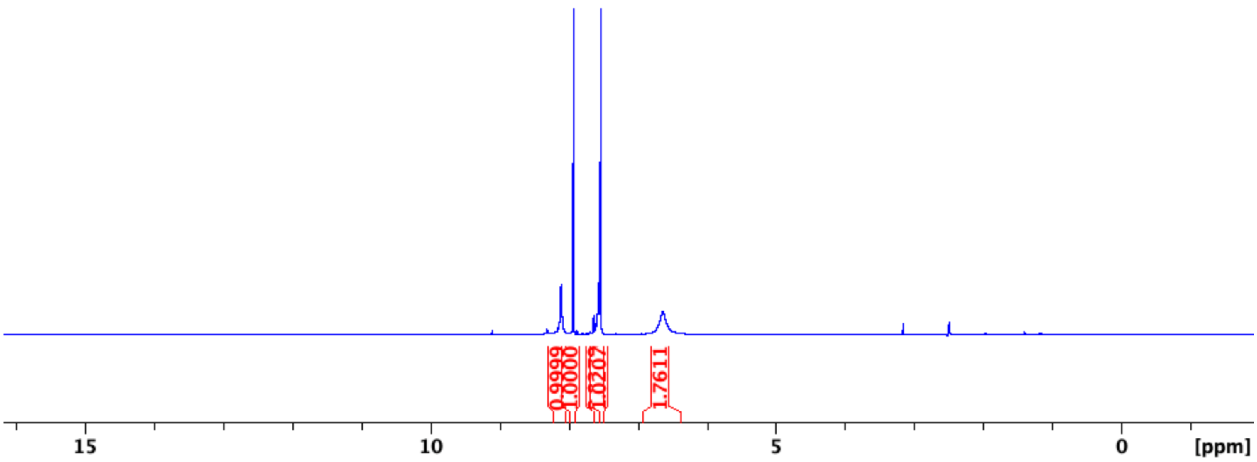

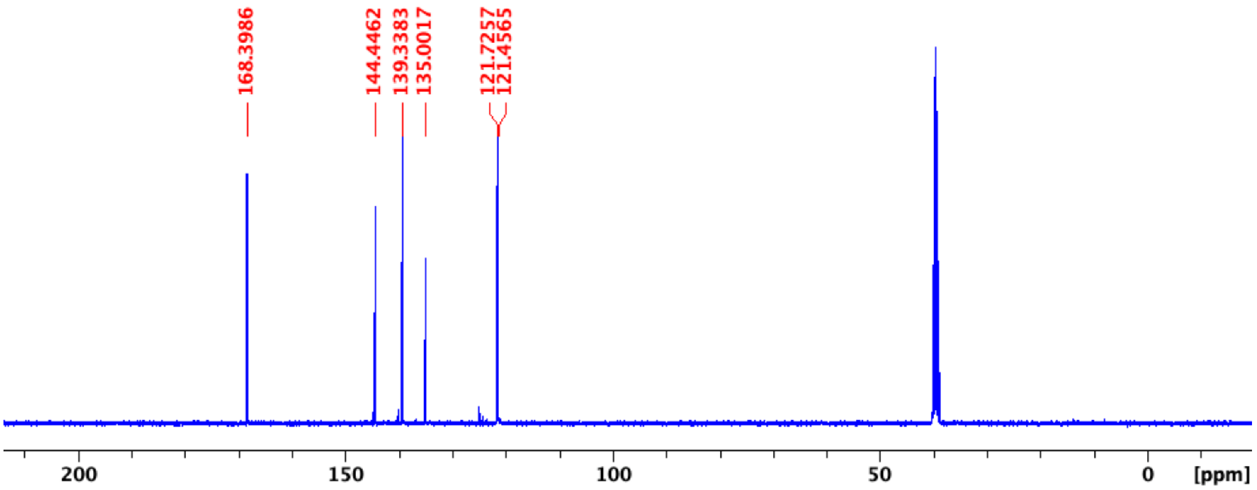

5-Amino-2-imidazolyl-pyridine-4-carboxamide (13b)

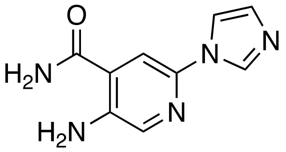

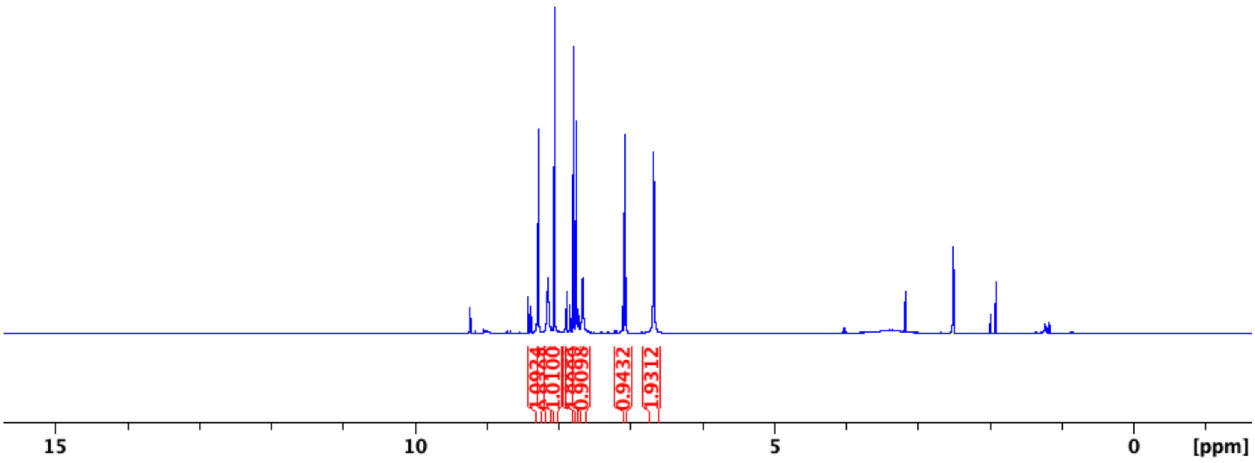

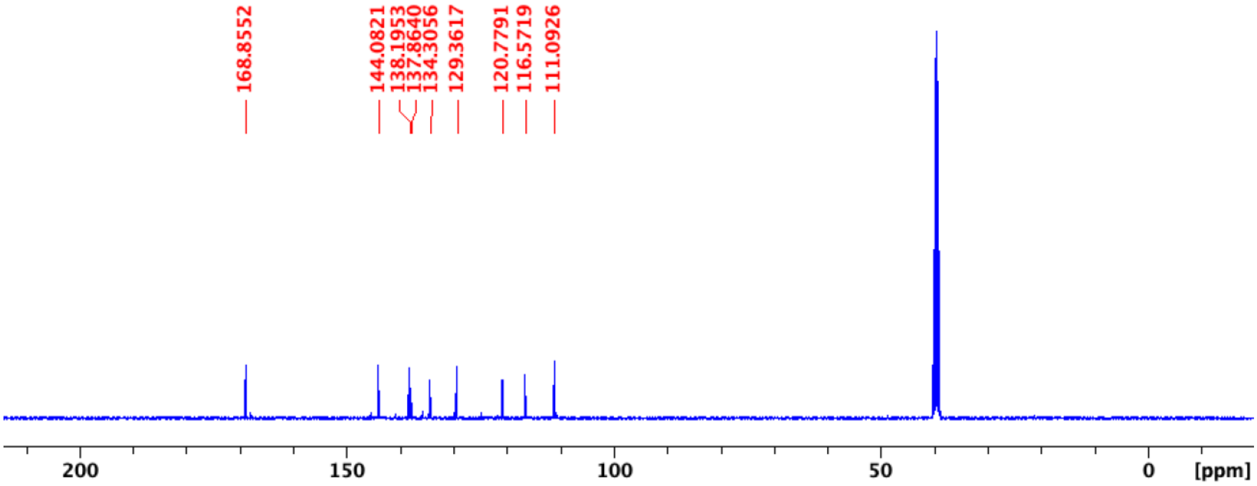

2-(2,3-Difluorobenzylmercapto)-6-chloropyrido[3,4-*d*]pyrimidine-4-one (15a)

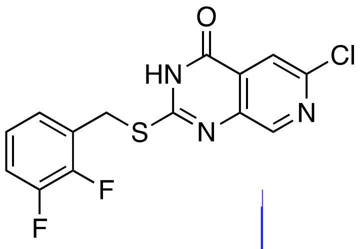

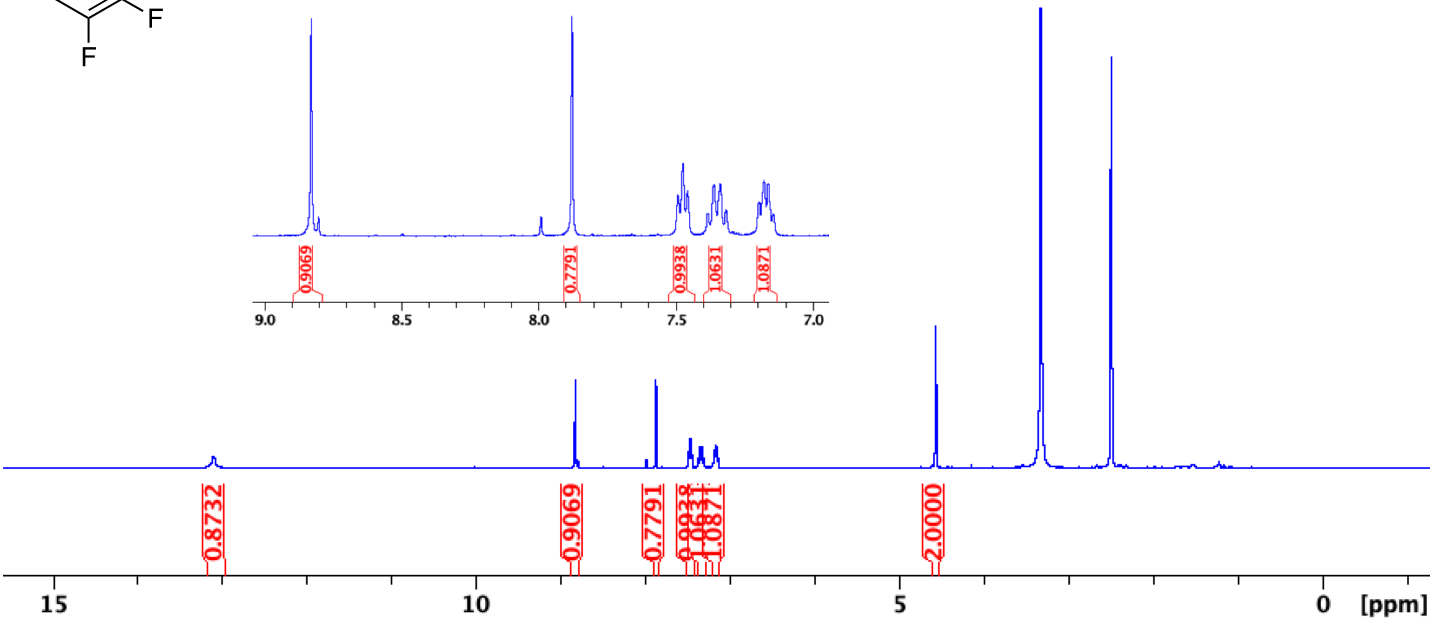

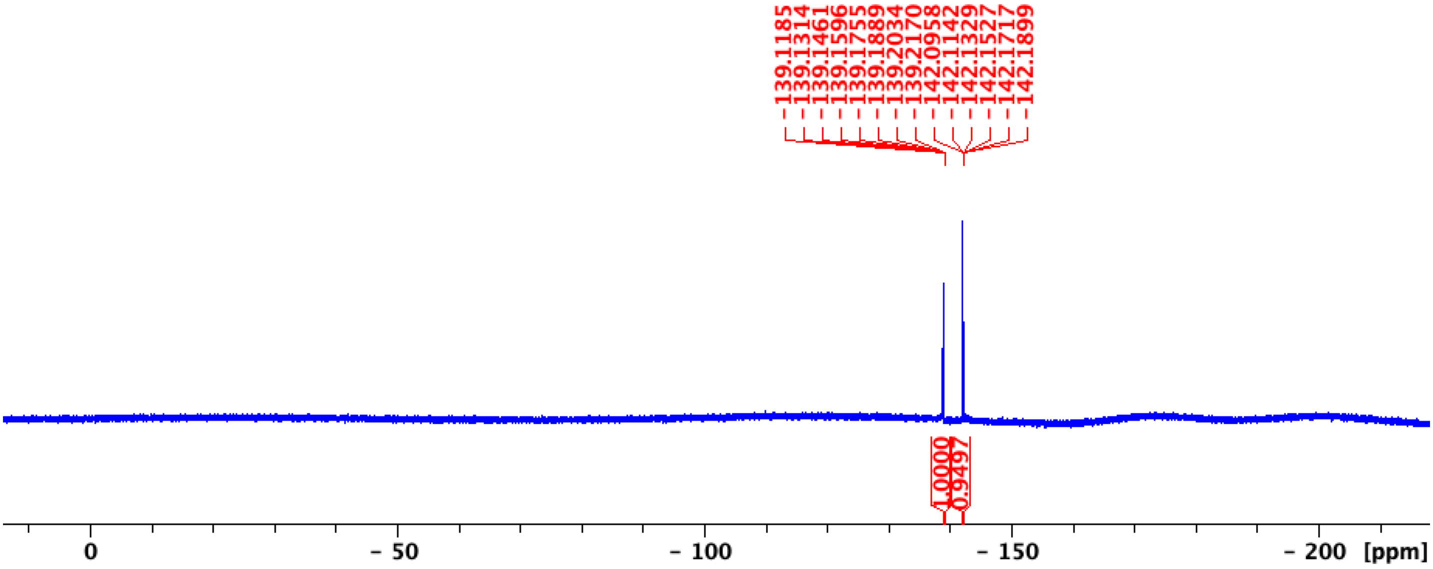

2-(2,3-Difluorobenzylthio)-6-imidazolyl-pyrido[3,4-*d*]pyrimidine-4-one (15b)

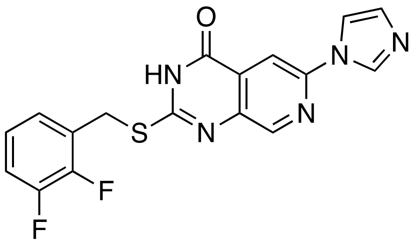

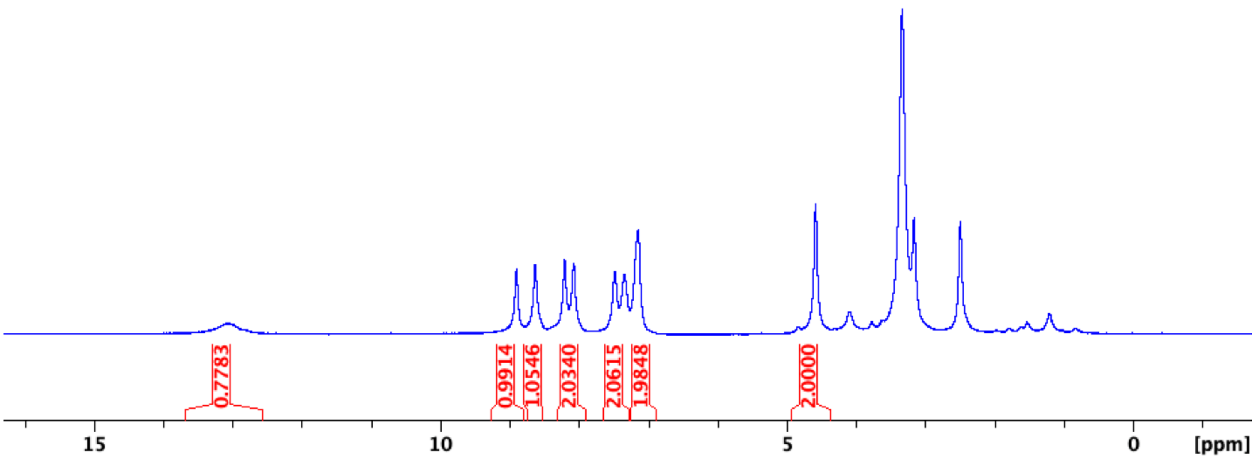

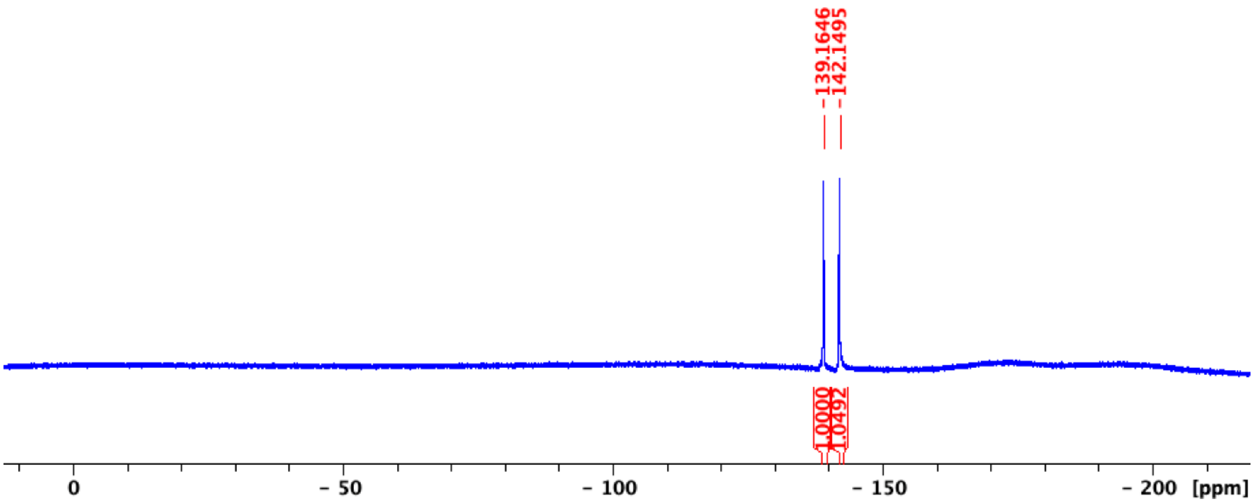

(2*R*)-2-[(6-Chloro-2-(2,3-difluorobenzylmercapto)-pyrido[3,4-*d*]pyrimidine-4-yl)amino]propanol (16a)

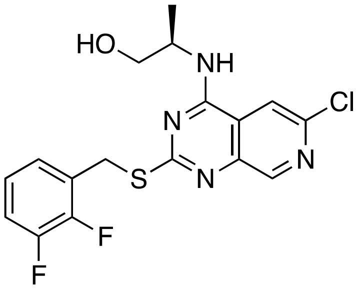

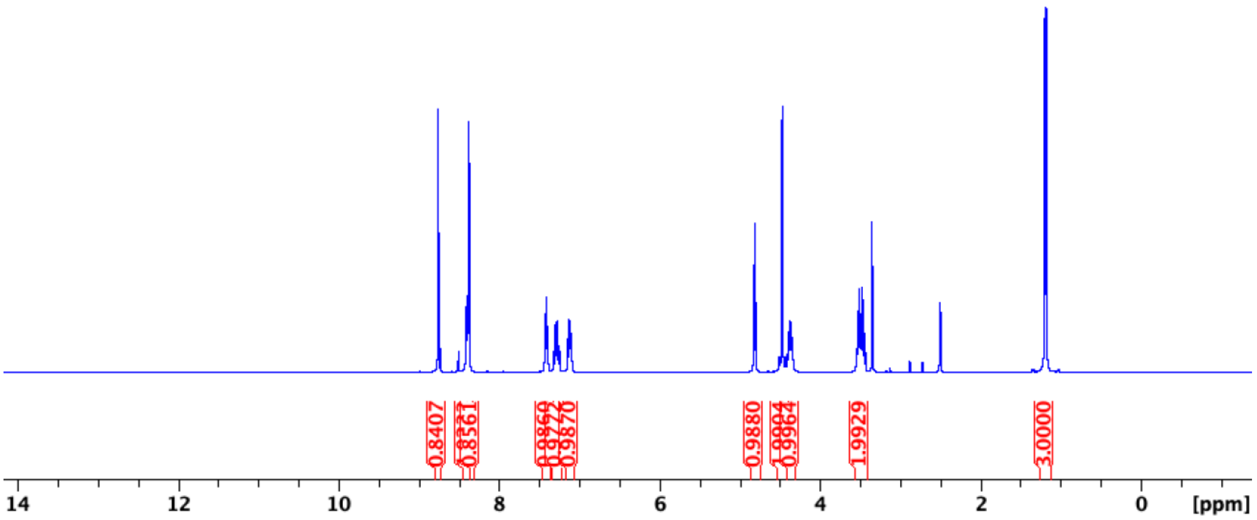

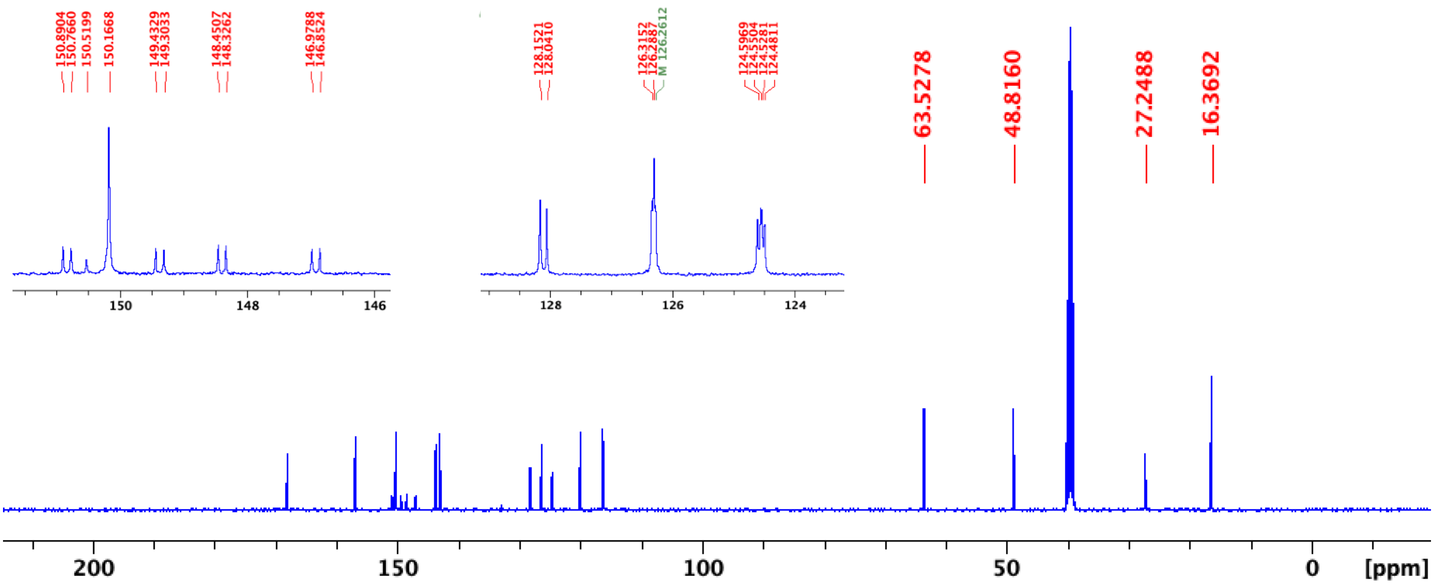

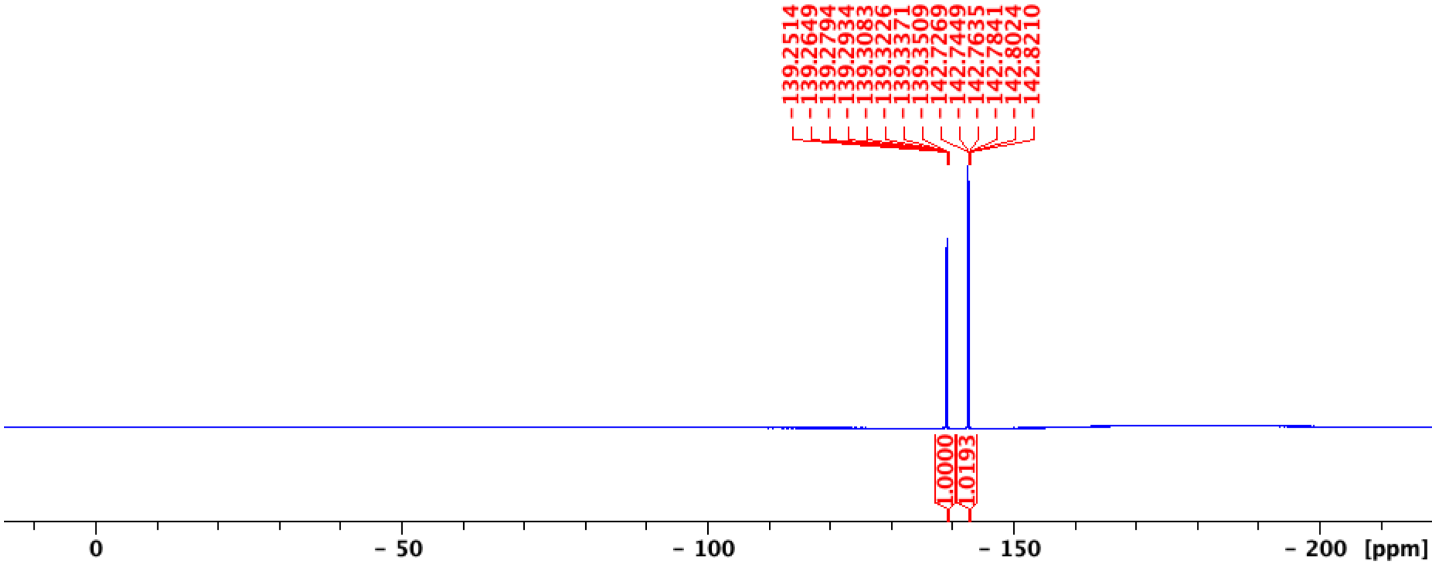

(2*R*)-2-[(6-Imidazolyl-2-(2,3-difluorobenzylmercapto)-pyrido[3,4-*d*]pyrimidine-4-yl)amino]propanol (16b)

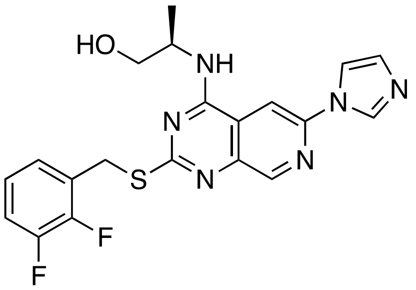

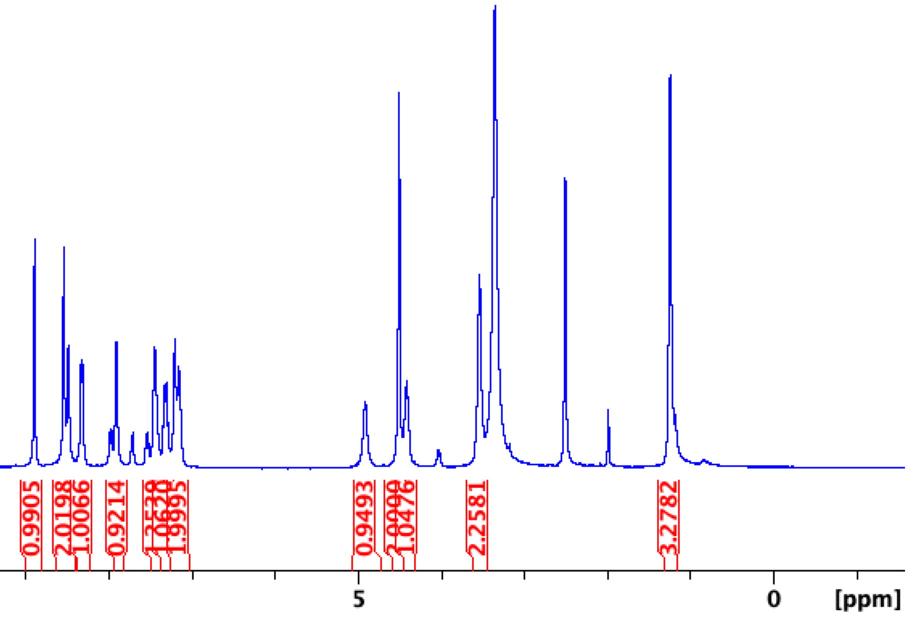

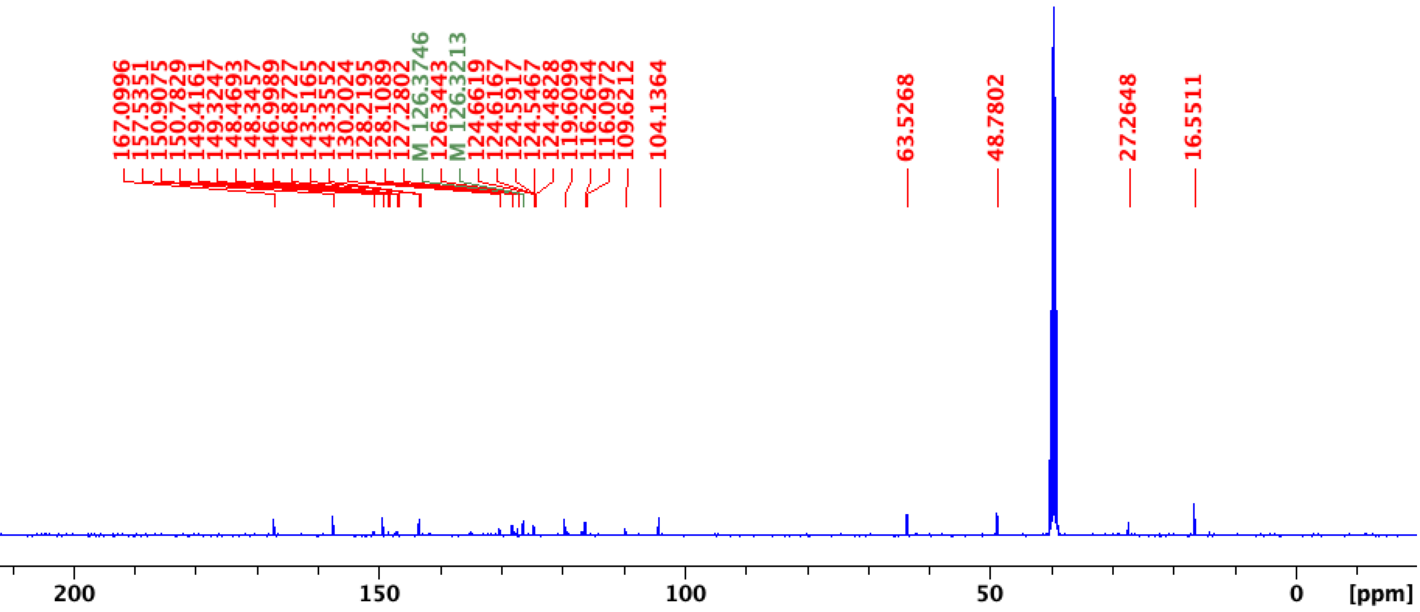
167.0996
157.5351
150.9075
150.7829
149.4161
149.3247
148.4693
148.3457
146.9989
146.8727
143.5165
143.3552
130.2024
128.2195
128.1089
127.2802
M 126.3746
126.3443
M 126.3213
124.6619
124.6167
124.5917
124.5467
124.4828
119.6099
116.2644
116.0972
109.6212
104.1364
63.5268
48.7802
27.2648
16.5511
200
150
100
50
0
[ppm]

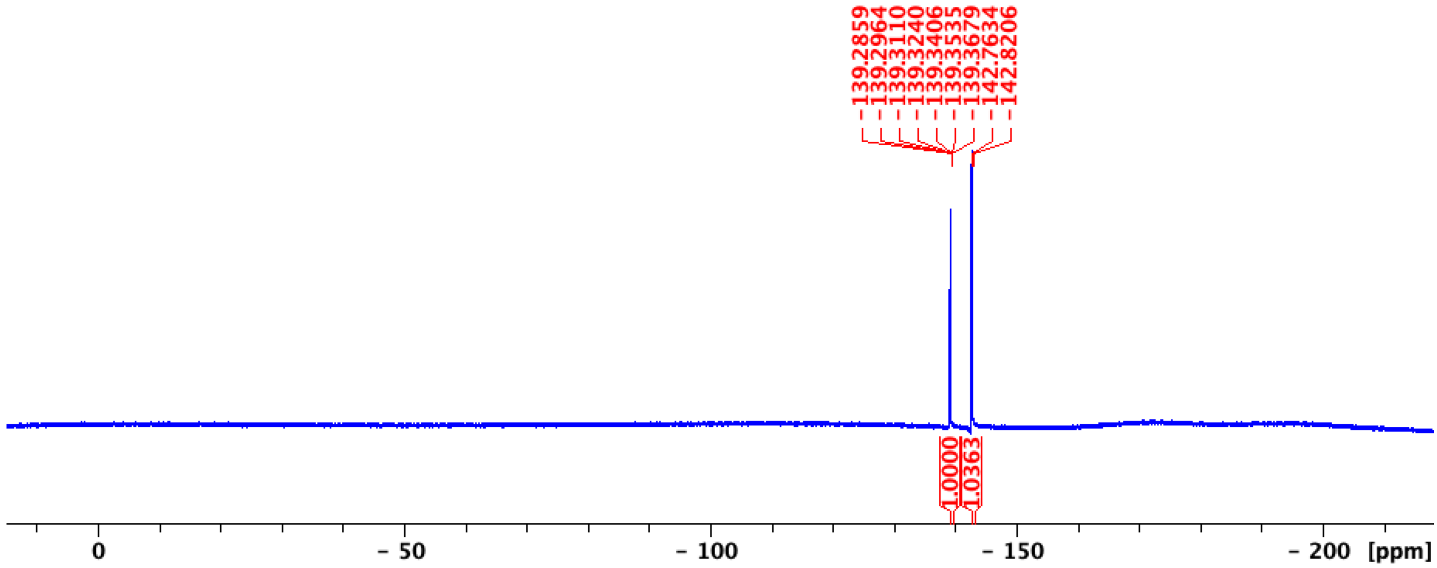
-139.2859
-139.2964
-139.3110
-139.3240
-139.3406
-139.3535
-139.3679
-142.7634
-142.8206
1.0000
1.0363
0
- 50
- 100
- 150
- 200
[ppm]

(2*R*)-2-[(2-(2,3-Difluorobenzylmercapto)-6-phenyl-pyrido[3,4-*d*]pyrimidine-4-yl)amino]propanol (17a)

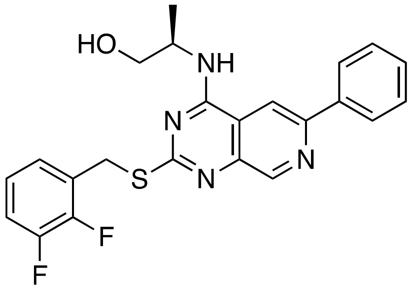

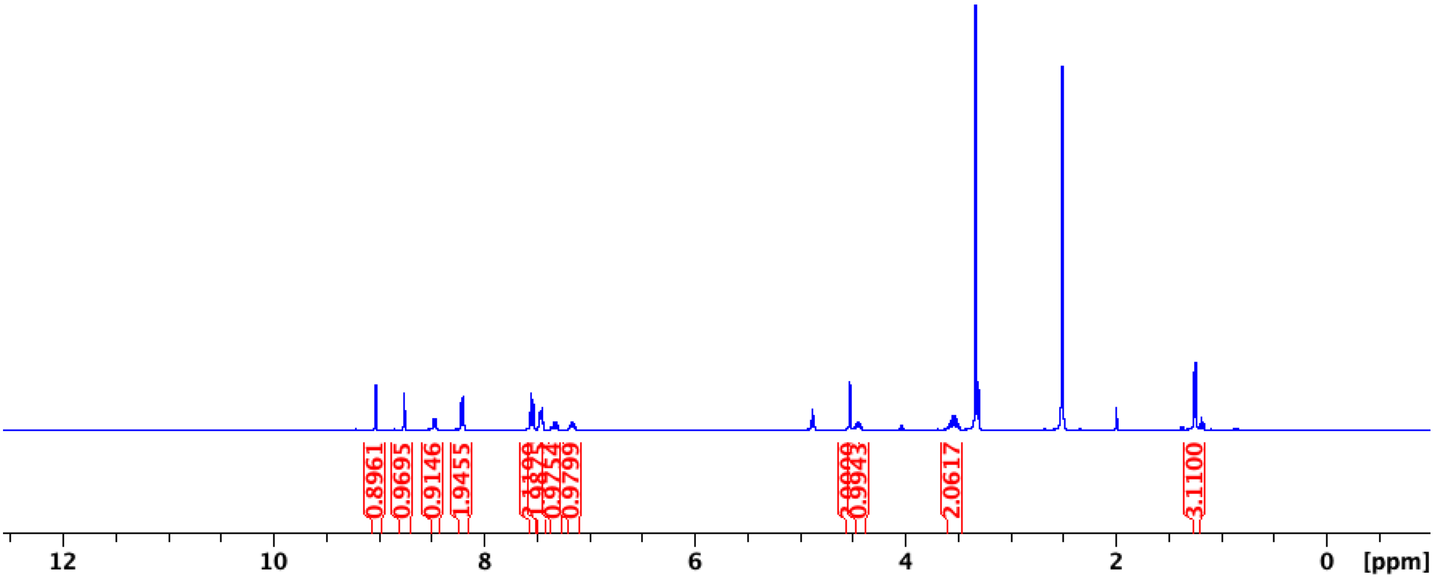

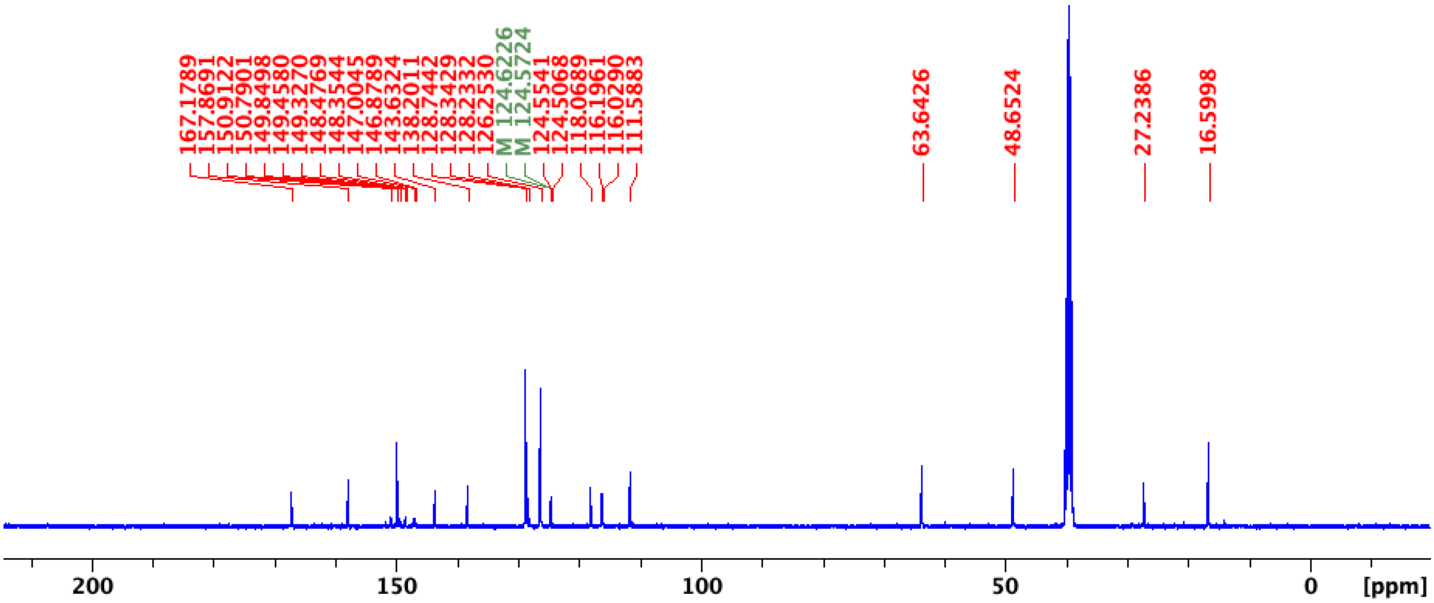

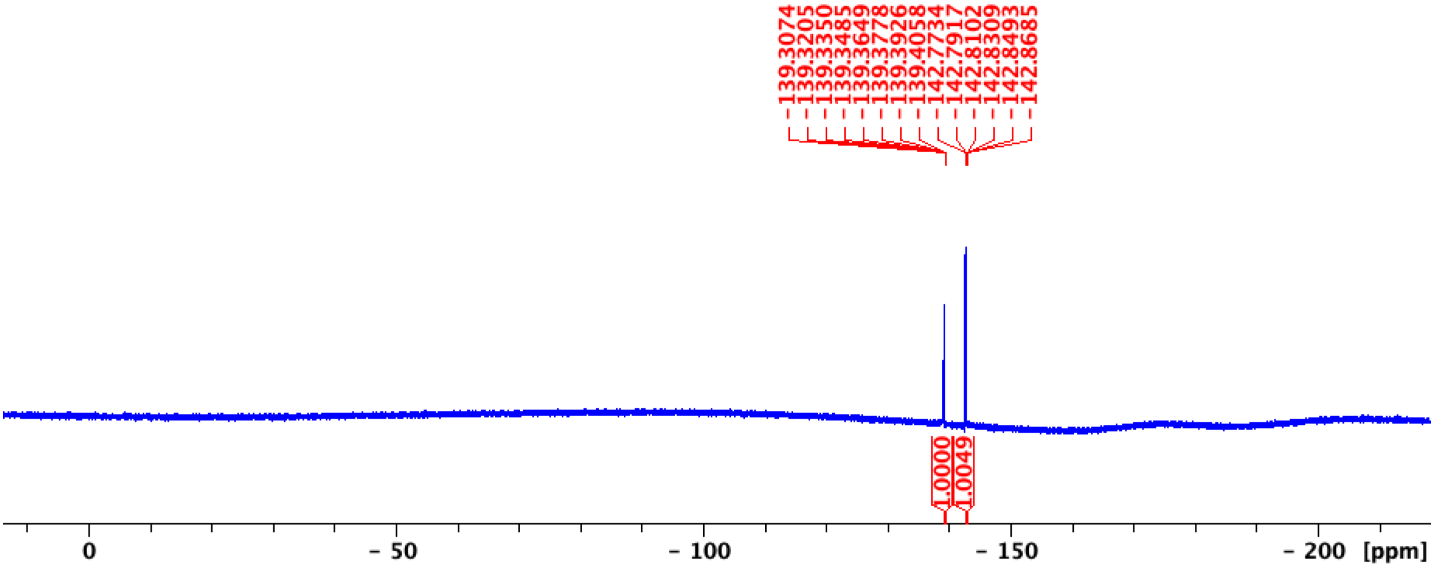

-139.3074
-139.3205
-139.3350
-139.3485
-139.3649
-139.3778
-139.3926
-139.4058
-142.7734
-142.7917
-142.8102
-142.8309
-142.8493
-142.8685
1.0000
1.0049
0
- 50
- 100
- 150
- 200
[ppm]

(2*R*)-2-[(2-(2,3-Difluorobenzylmercapto)-6-(2-furanyl)-pyrido[3,4-*d*]pyrimidine-4-yl)amino]propanol (17b)

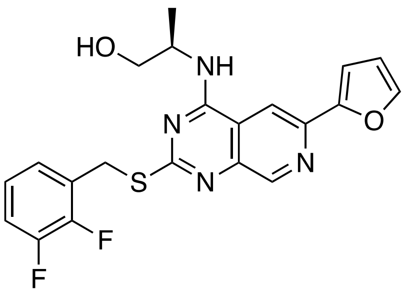

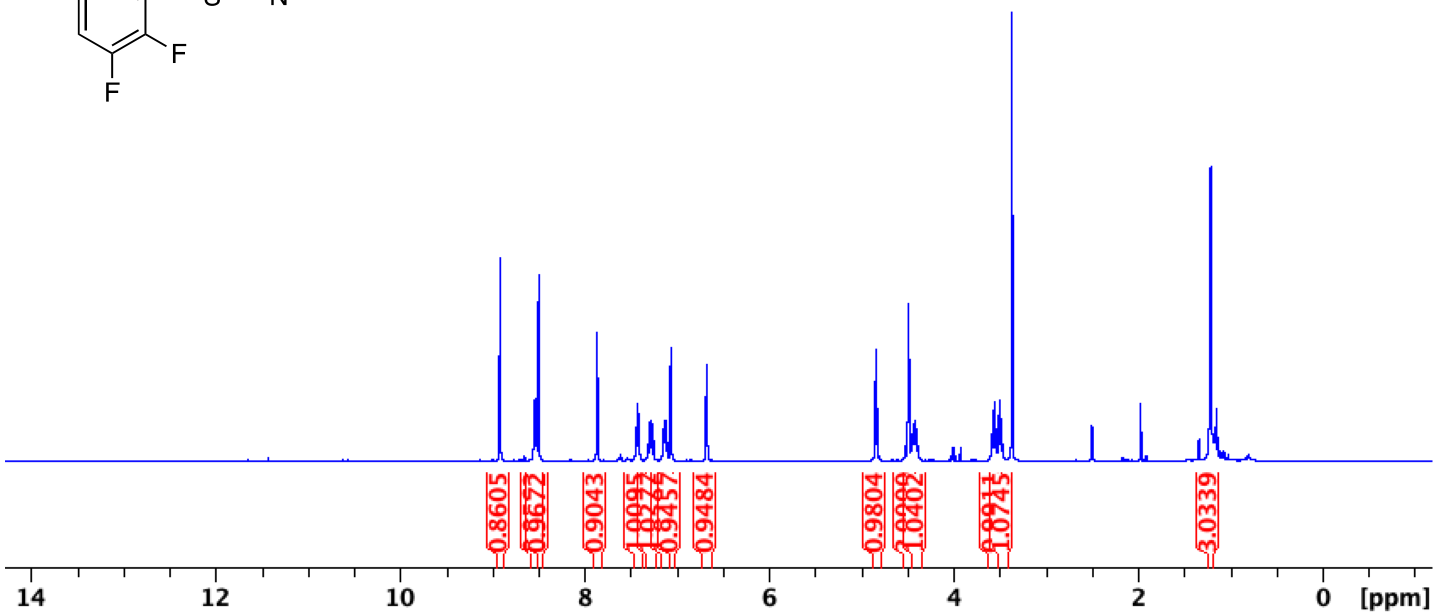

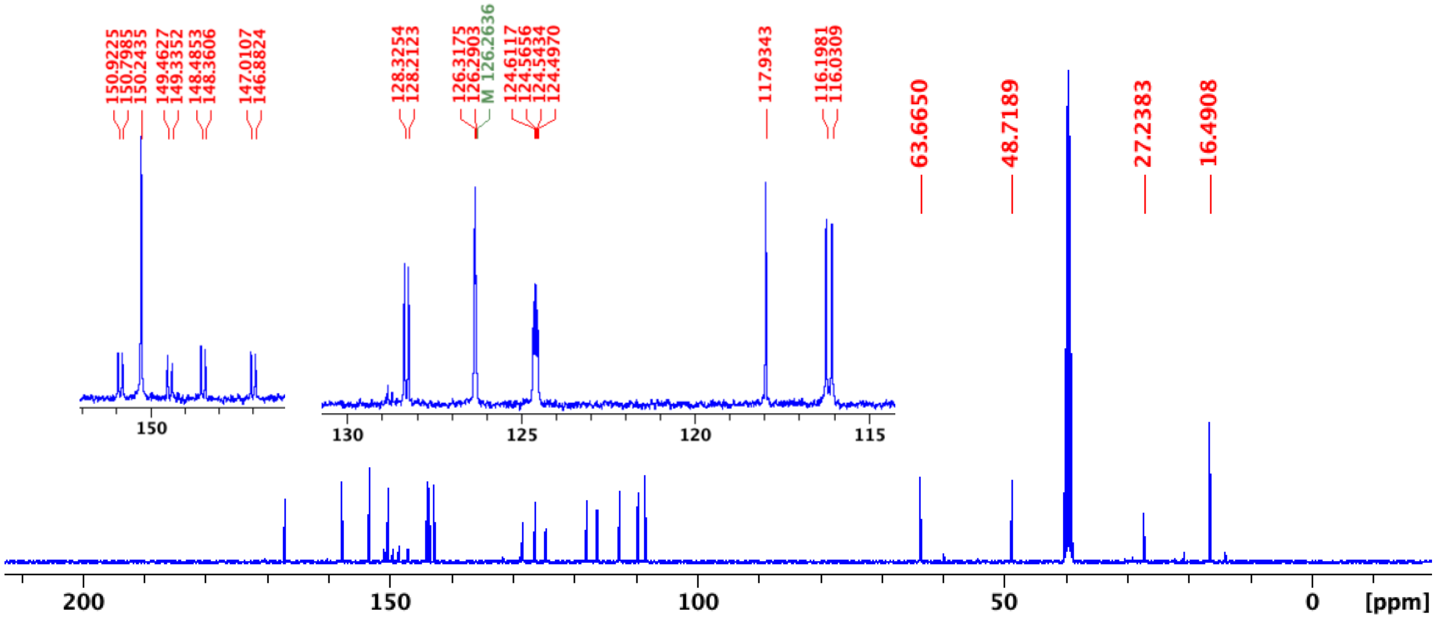

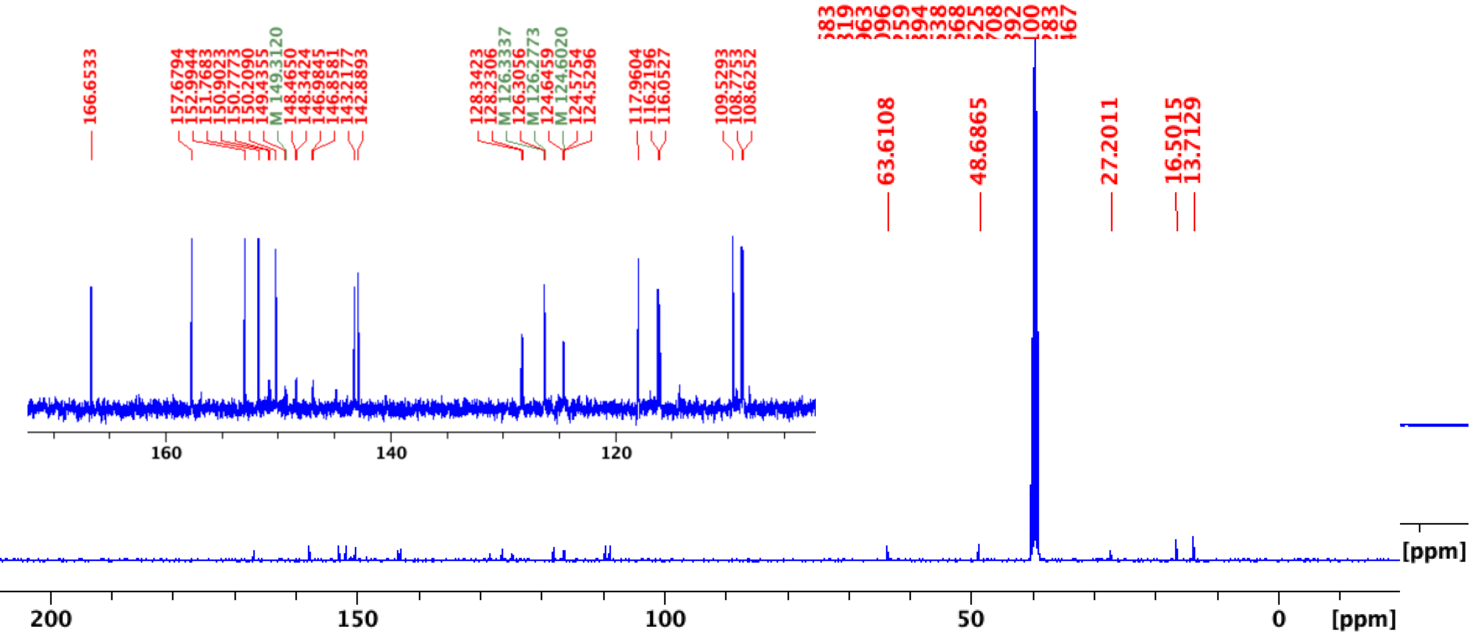

(2*R*)-2-[(2-(2,3-Difluorobenzylmercapto)-6-(5-methylfuran-2-yl)-pyrido[3,4-*d*]pyrimidine-4-yl)amino]propanol (17c)

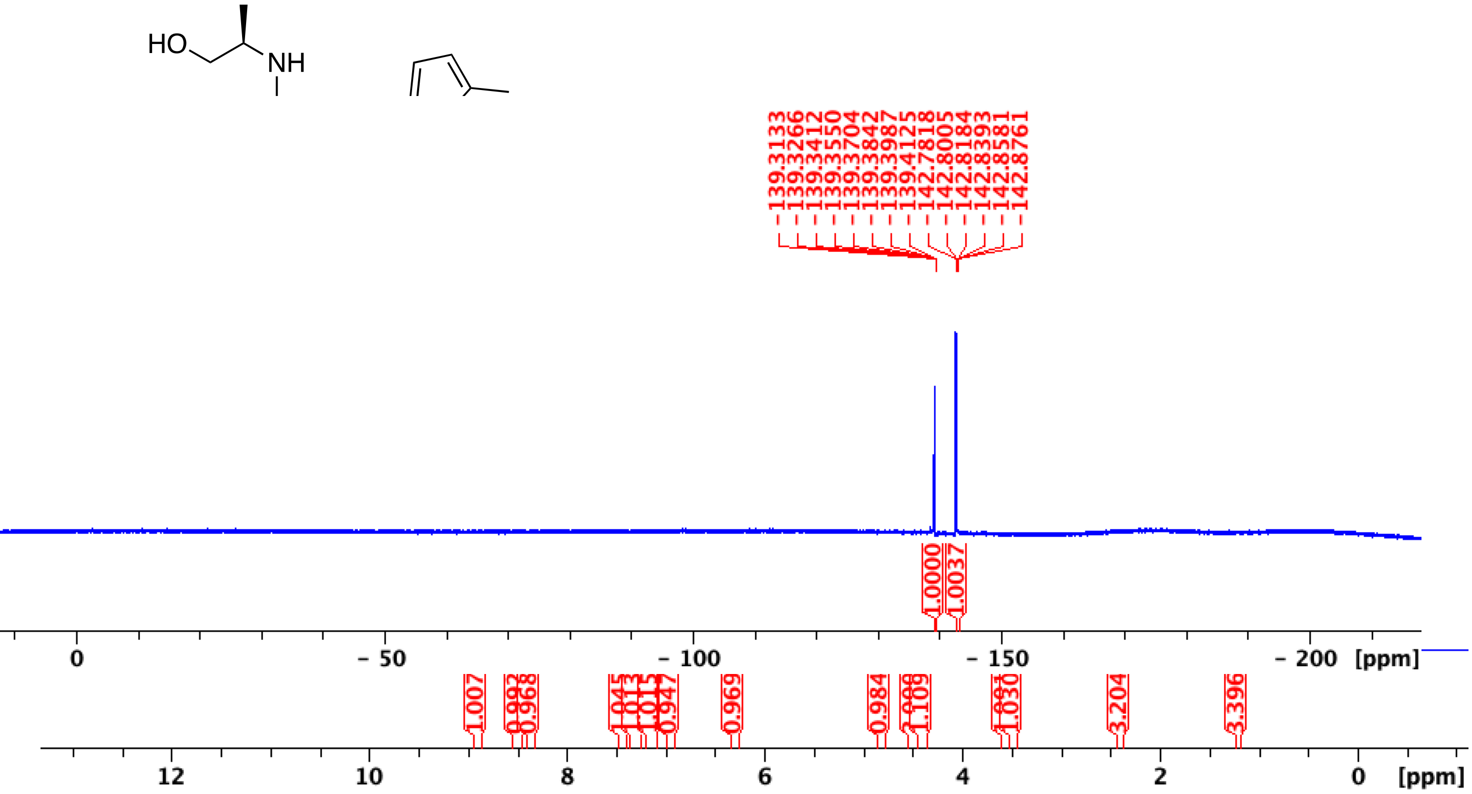

(2*R*)-2-[(2-(2,3-Difluorobenzylmercapto)-6-(2-thienyl)-pyrido[3,4-*d*]pyrimidine-4-yl)amino]propanol (17d)

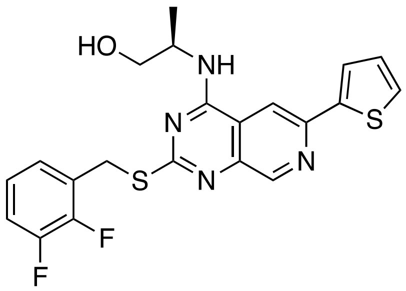

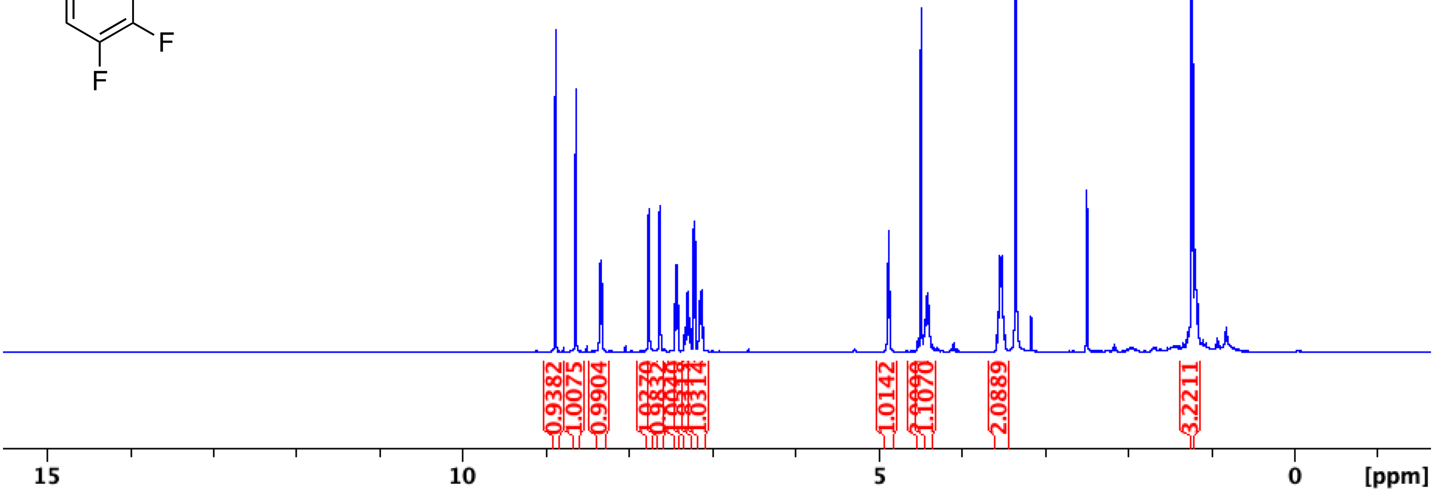

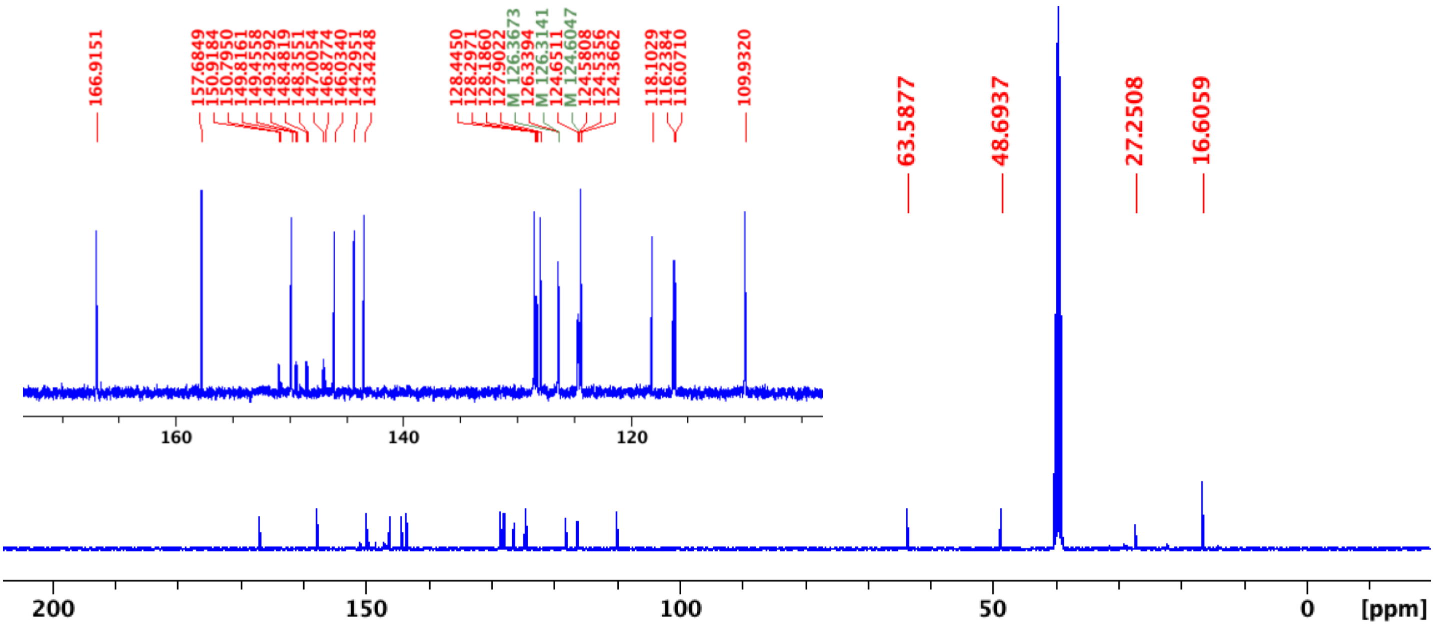

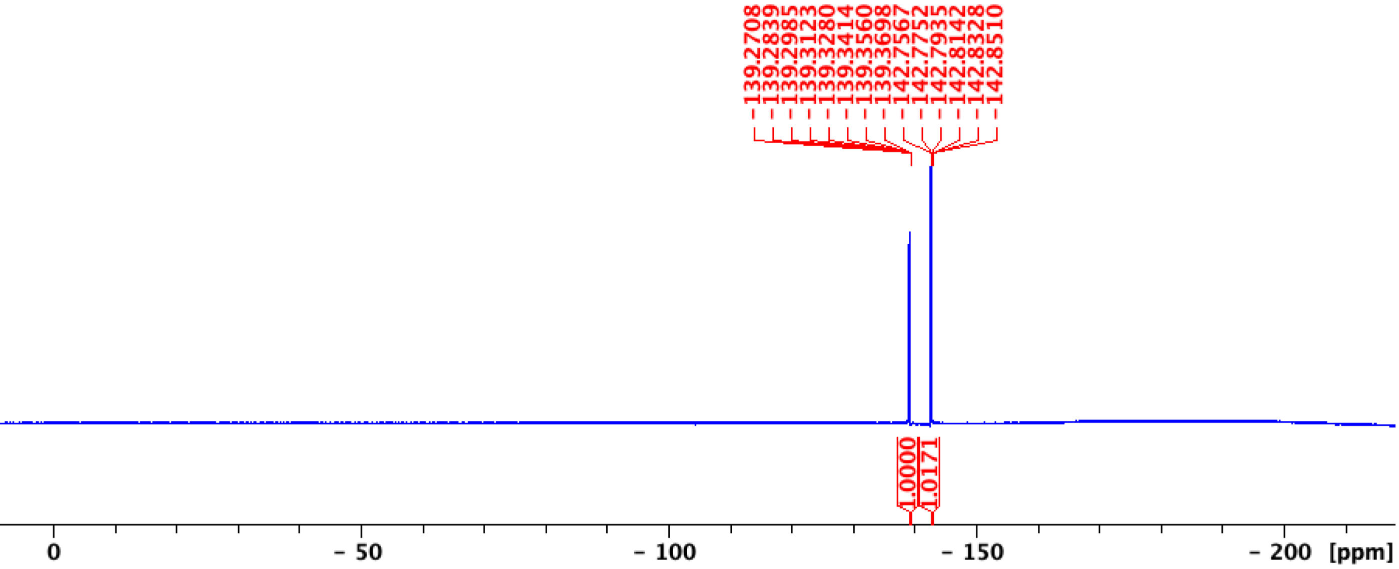
-139.2708
-139.2839
-139.2985
-139.3123
-139.3280
-139.3414
-139.3560
-139.3698
-142.7567
-142.7752
-142.7935
-142.8142
-142.8328
-142.8510
1.0000
1.0171
0
- 50
- 100
- 150
- 200
[ppm]

6-Chloro-2-(2,3-difluorobenzylmercapto)-4-(4-methoxybenzyl)-pyrido[3,4-*d*]pyrimidine (18)

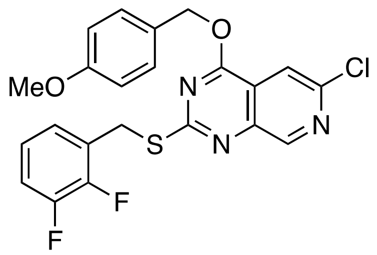

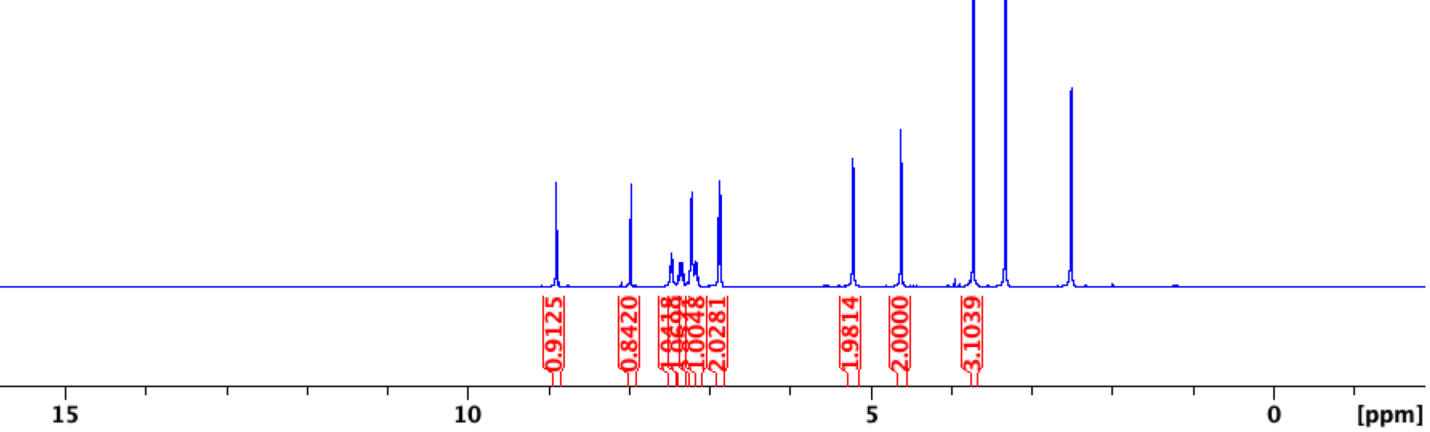

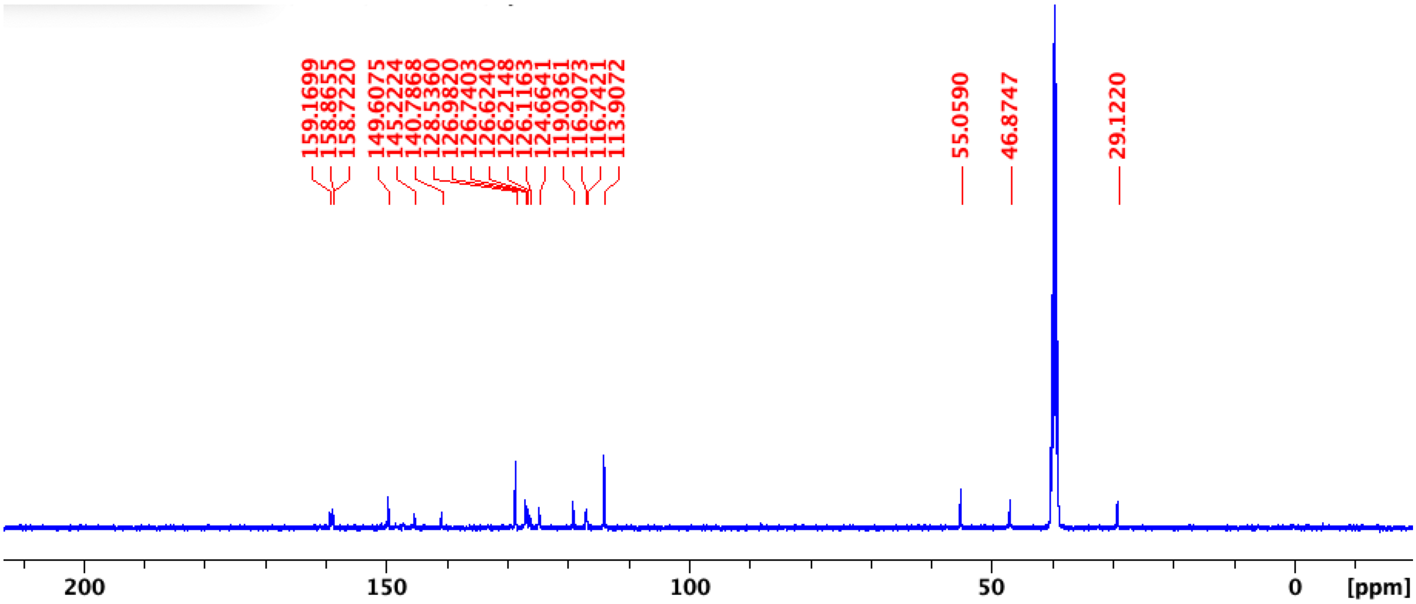

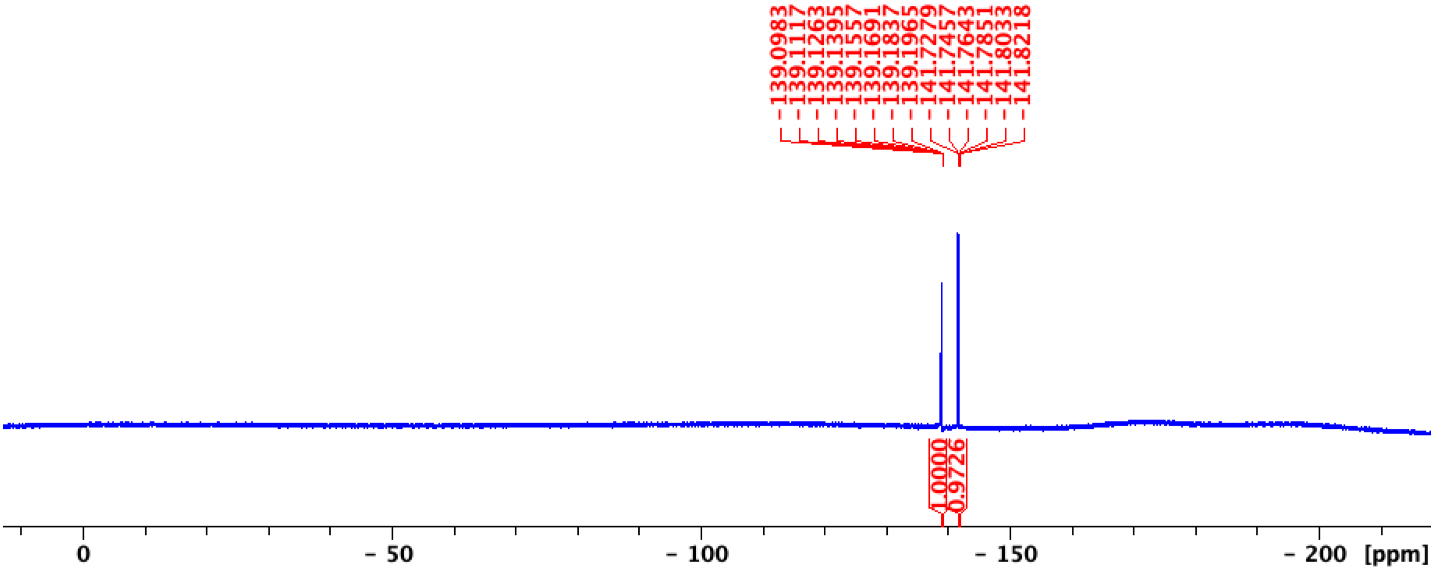

2-(2,3-Difluorobenzylmercapto)-4-(4-methoxybenzyl)-(*N*-diphenylmethylene)-pyrido[3,4-*d*]pyrimidine-6-amine (19)

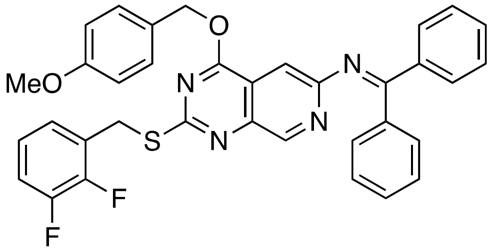

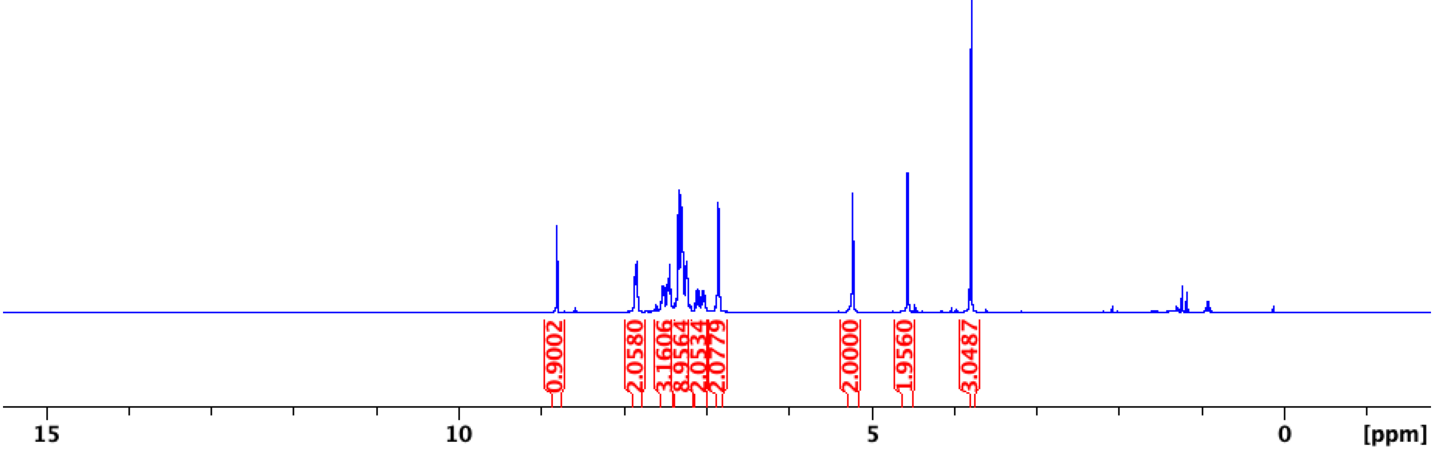

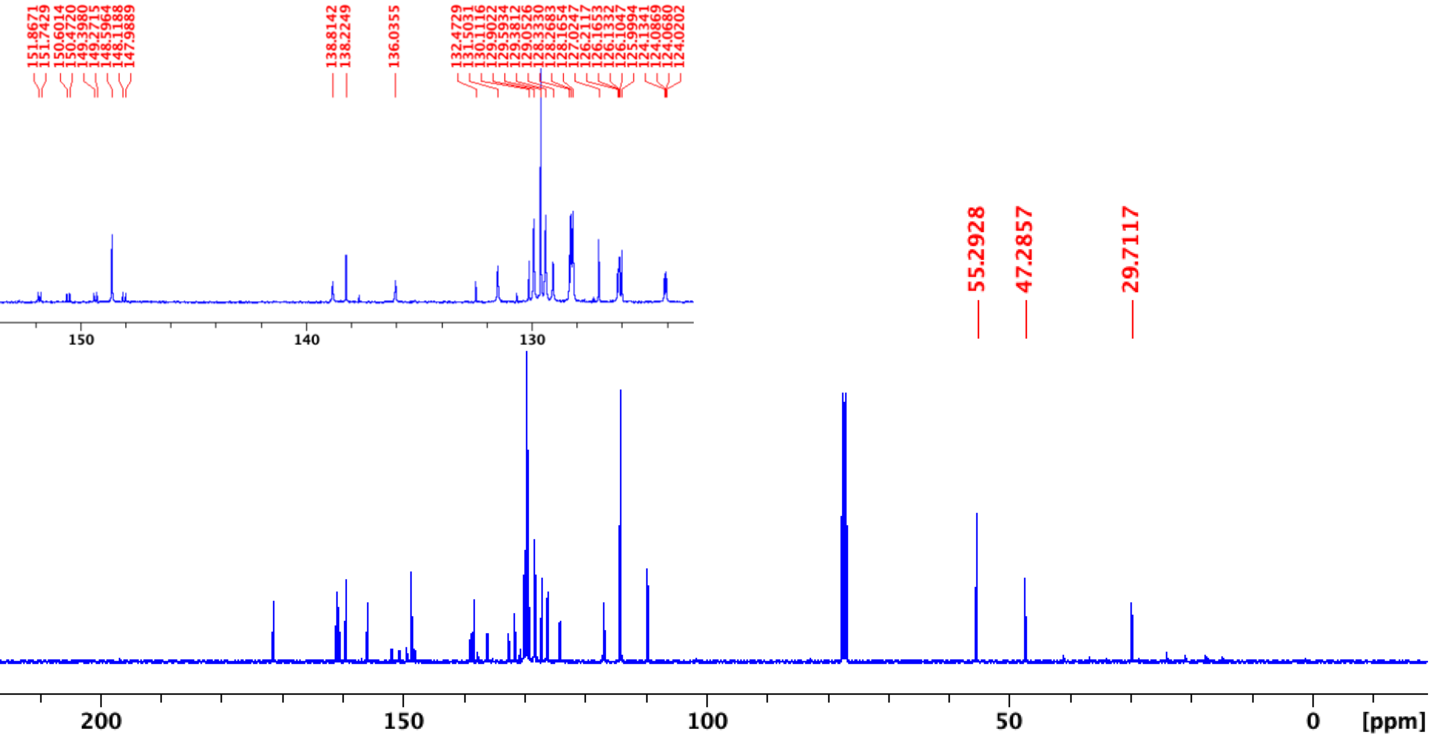
151.8671
151.7429
150.6014
150.4720
149.3980
149.2715
148.5964
148.1188
147.9889
138.8142
138.2249
136.0355
132.4729
131.5031
130.1116
129.9022
129.5934
129.3812
129.0526
128.3330
128.2683
128.1654
127.0247
126.2117
126.1653
126.1332
126.1047
125.9994
124.1341
124.0869
124.0680
124.0202
150
140
130
55.2928
47.2857
29.7117
200
150
100
50
0
[ppm]

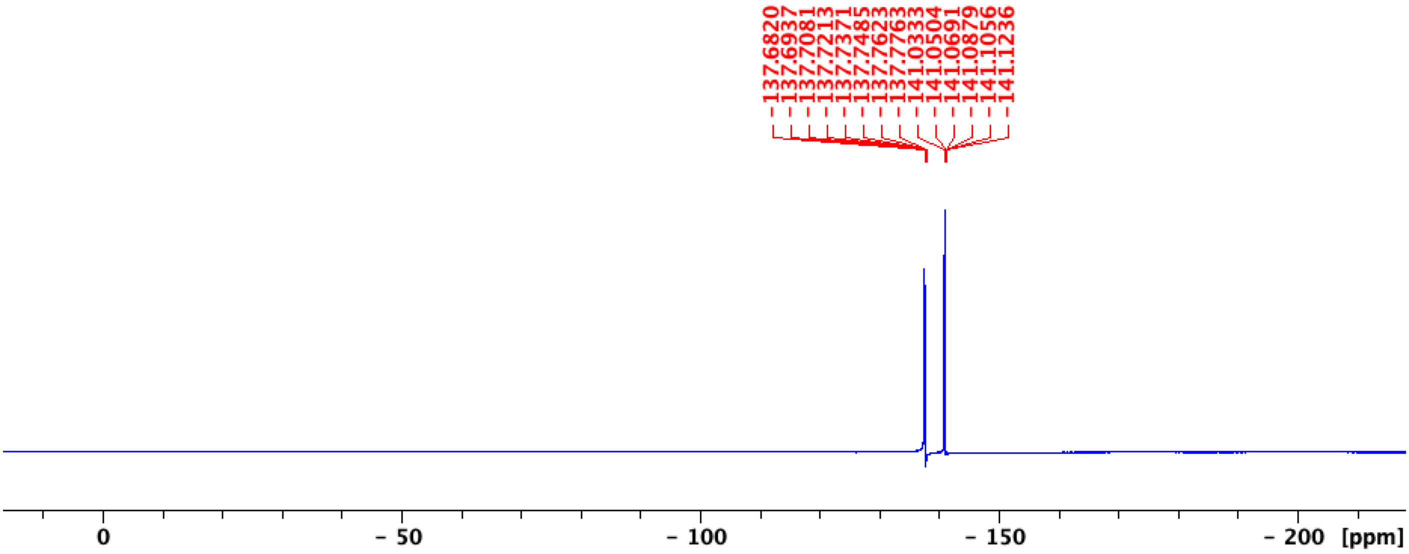
-137.6820
-137.6937
-137.7081
-137.7213
-137.7371
-137.7485
-137.7623
-137.7763
-141.0333
-141.0504
-141.0691
-141.0879
-141.1056
-141.1236
0
- 50
- 100
- 150
- 200
[ppm]

(2*R*)-2-[(6-Amino-2-(2,3-difluorobenzylmercapto)-pyrido[3,4-*d*]pyrimidine-4-yl)amino]propanol (21)

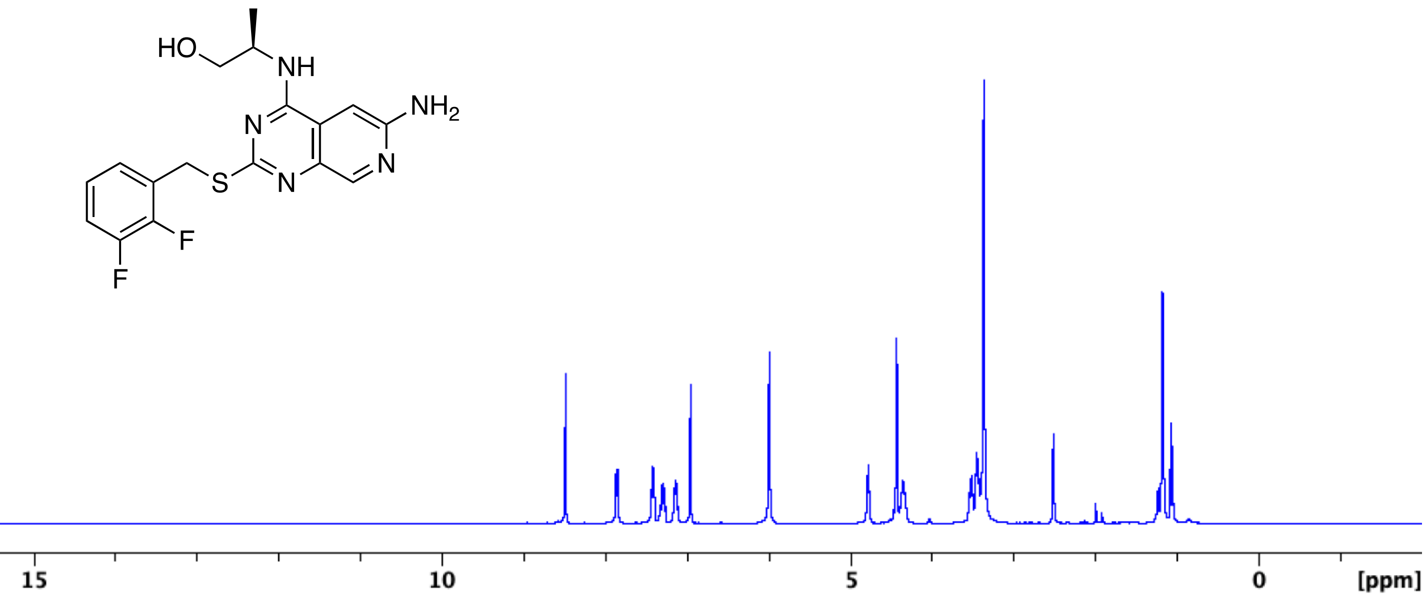

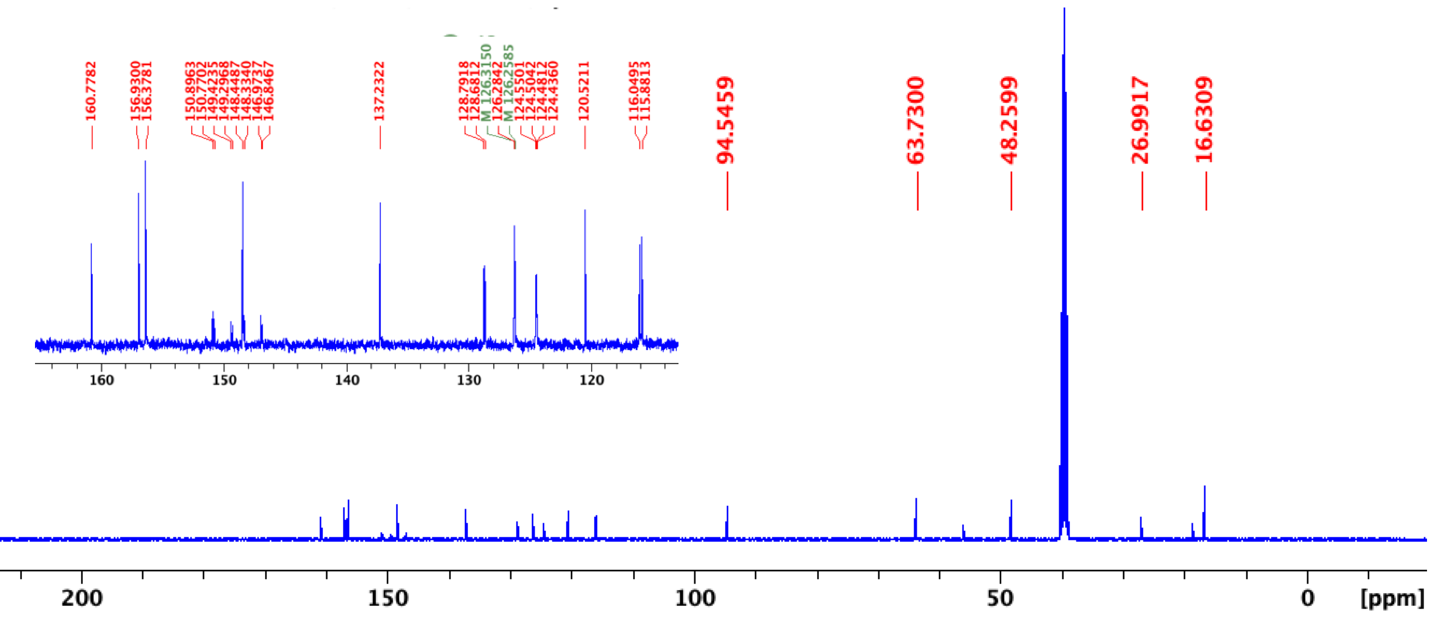

(2*R*)-2-[(6-Chloro-2-pyrido[3,4-*d*]pyrimidine-4-yl)amino]butanol (23a)

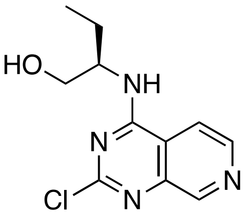
HO
NH
N
N
N
Cl

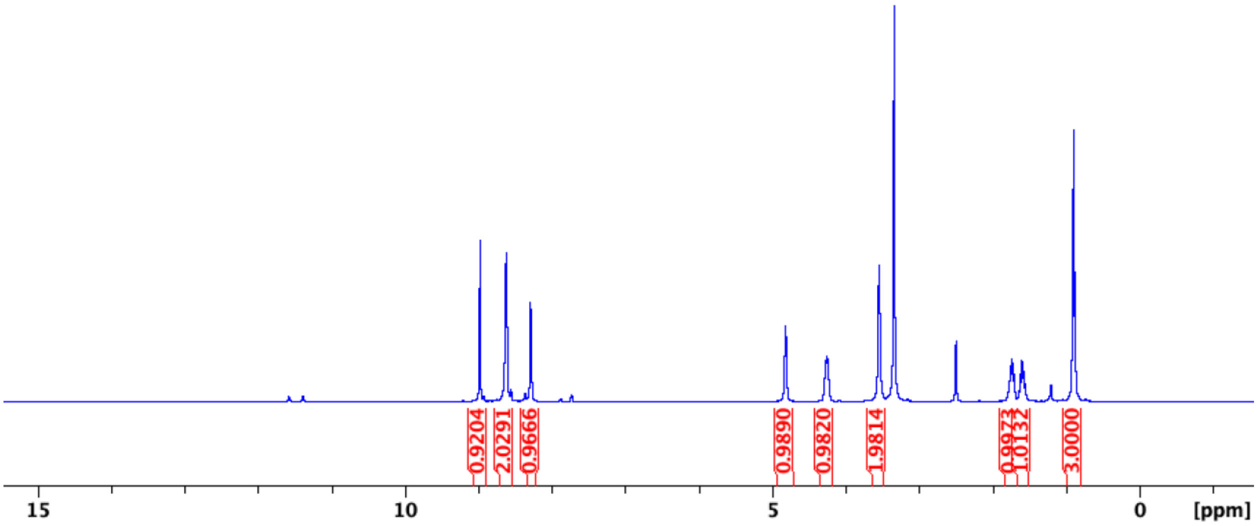
0.9204
2.0291
0.9666
0.9890
0.9820
1.9814
0.9973
1.0132
3.0000
15
10
5
0
[ppm]

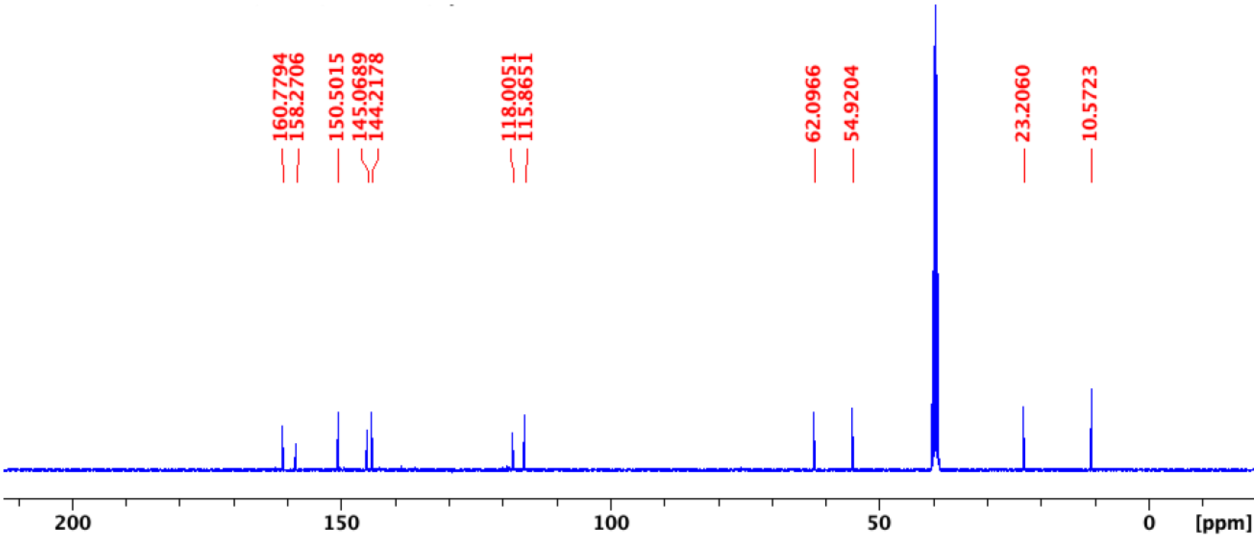
160.7794
158.2706
150.5015
145.0689
144.2178
118.0051
115.8651
62.0966
54.9204
23.2060
10.5723
200
150
100
50
0
[ppm]

2-[(2-Chloro-pyrido[3,4-*d*]pyrimidine-4-yl)amino]-2-methyl-propan-1-ol (23b)

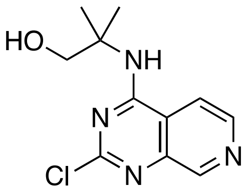

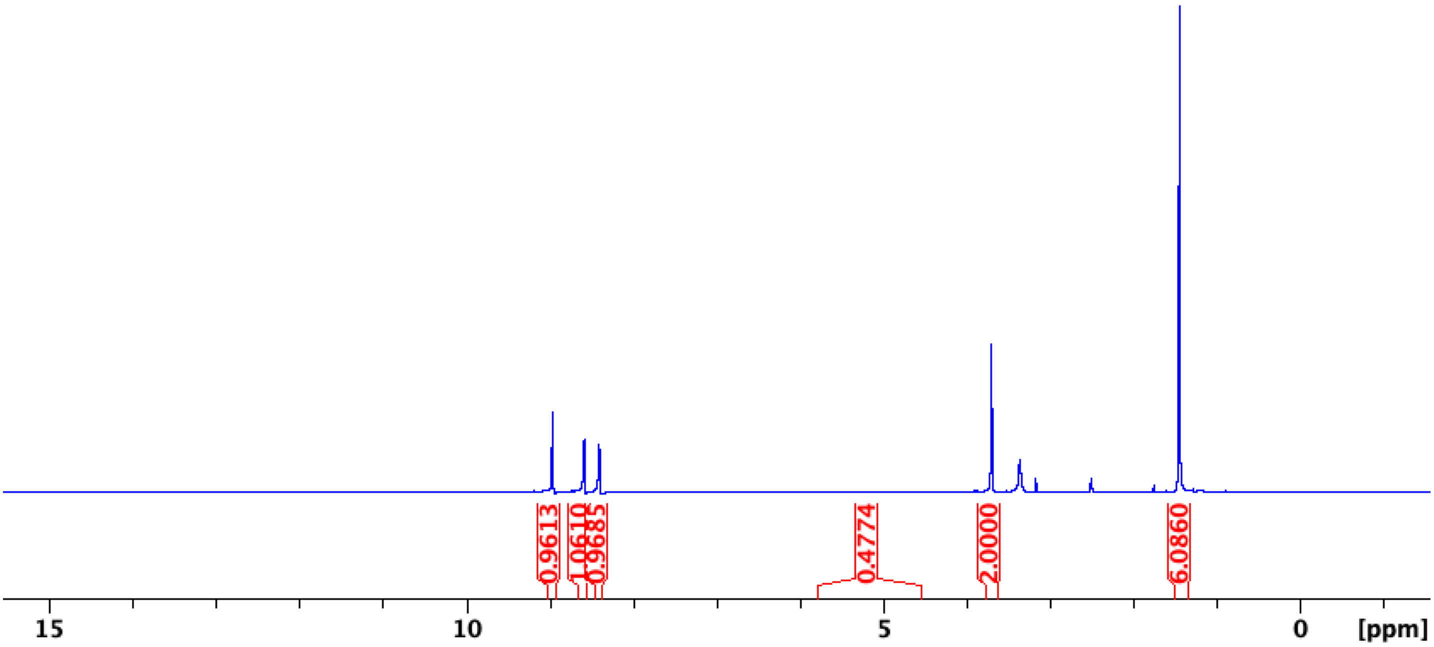

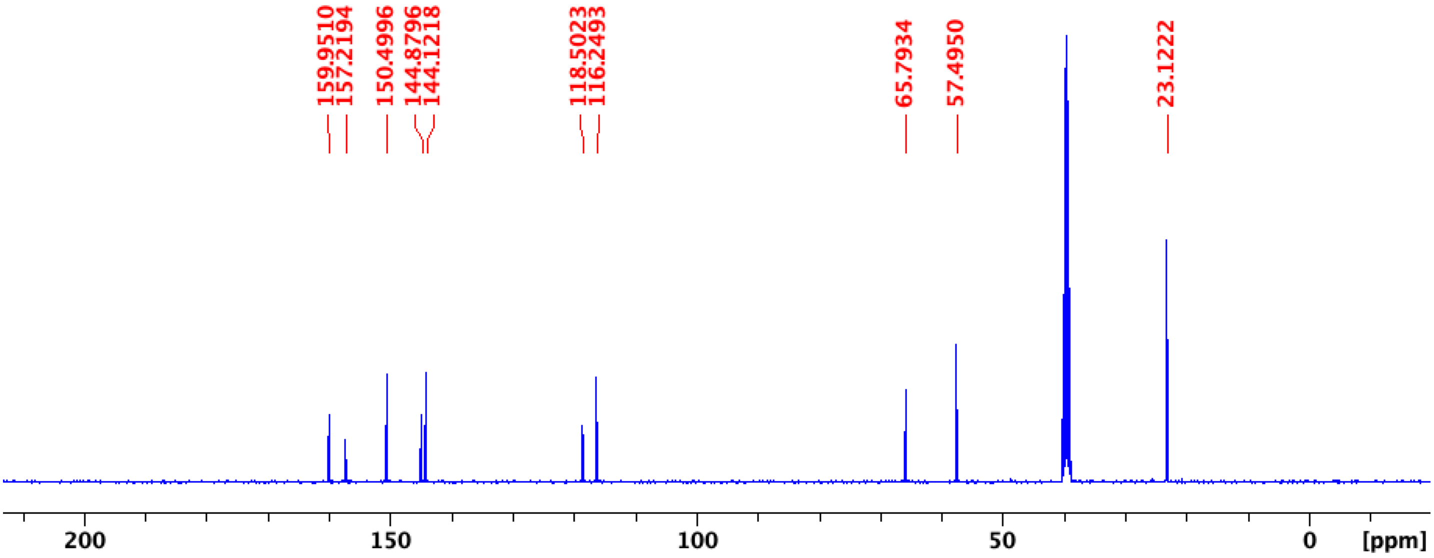

3-[(2-Chloro-pyrido[3,4-*d*]pyrimidine-4-yl)amino]propan-1,2-diol (23c)

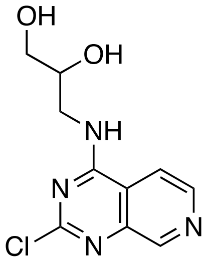

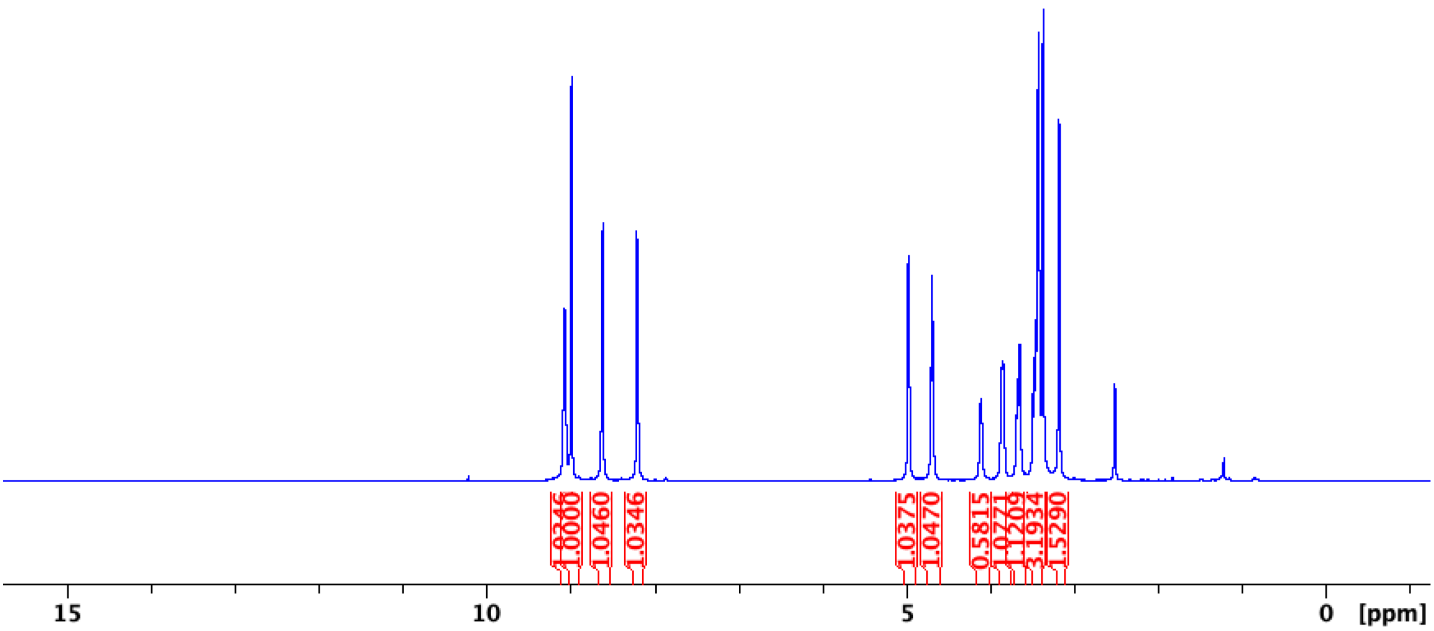

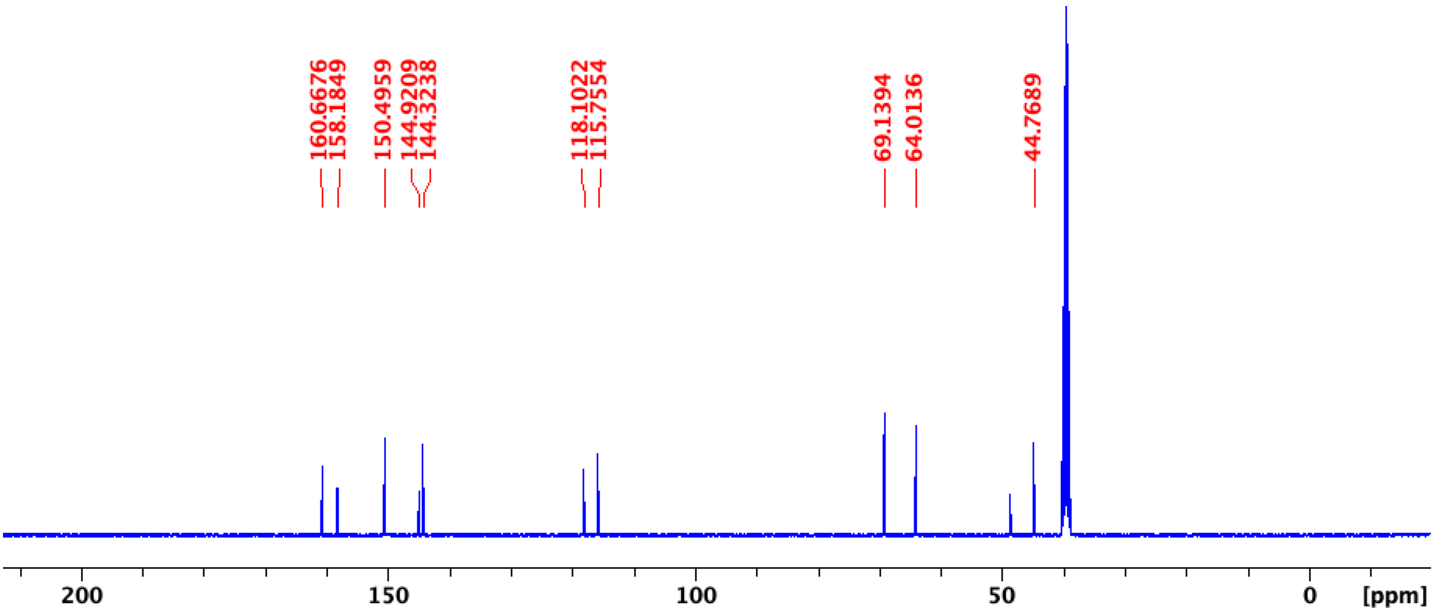

2-[(2-Chloro-pyrido[3,4-*d*]pyrimidine-4-yl)amino]ethanol (23d)

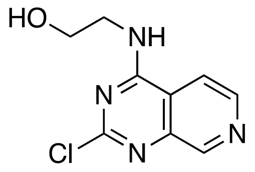
HO
NH
N
N
Cl
N

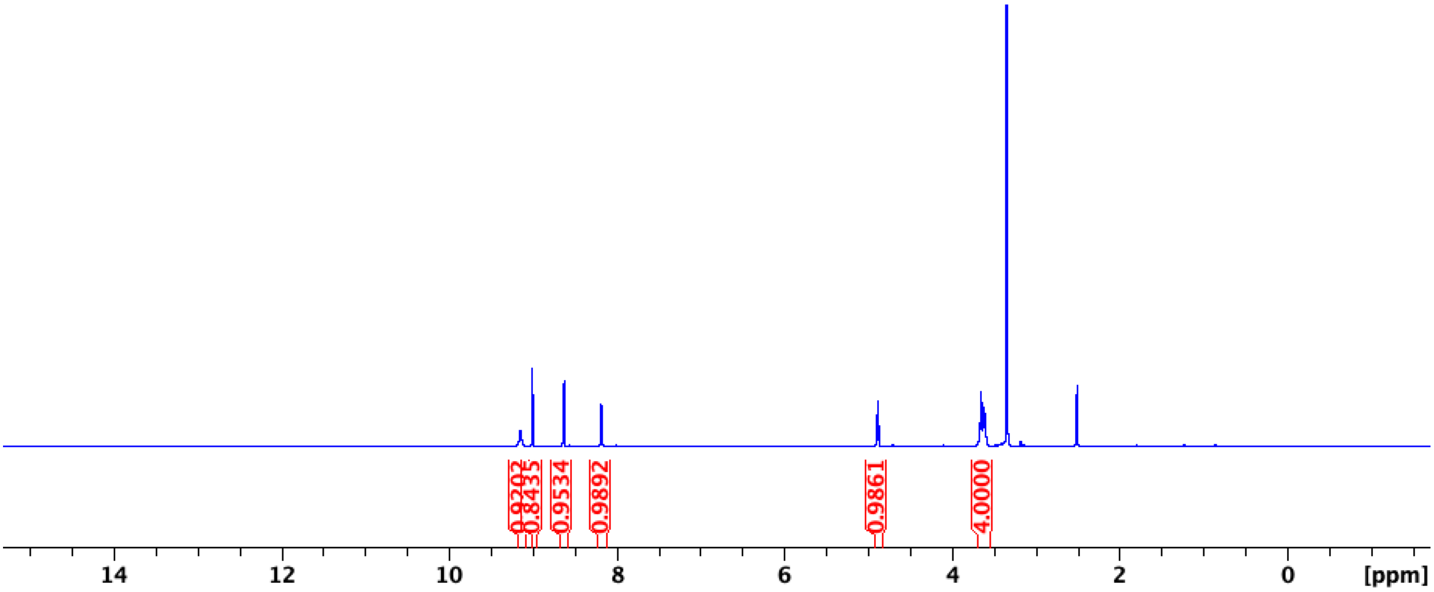
0.9202
0.8435
0.9534
0.9892
0.9861
4.0000
14
12
10
8
6
4
2
0
[ppm]

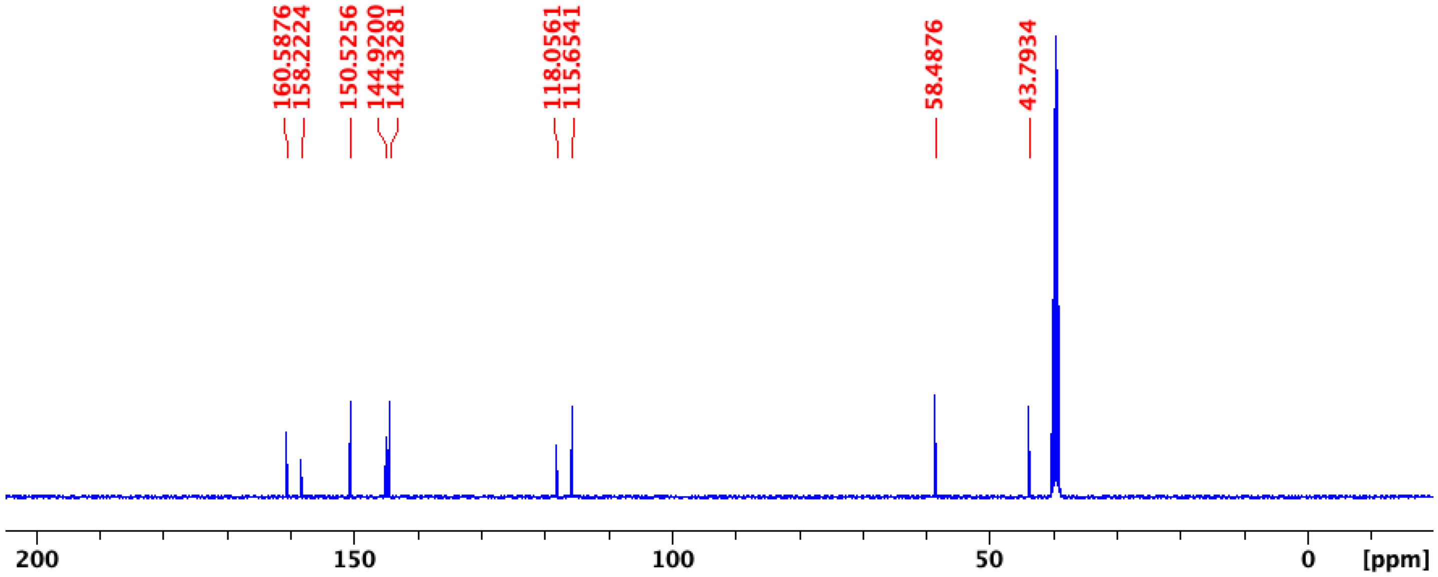
160.5876
158.2224
150.5256
144.9200
144.3281
118.0561
115.6541
58.4876
43.7934
200
150
100
50
0
[ppm]

3-[(2-(2,3-Difluorobenzylmercapto)-pyrido[3,4-*d*]pyrimidine-4-yl)oxy]ethanol (23e)

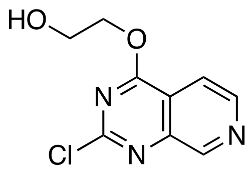

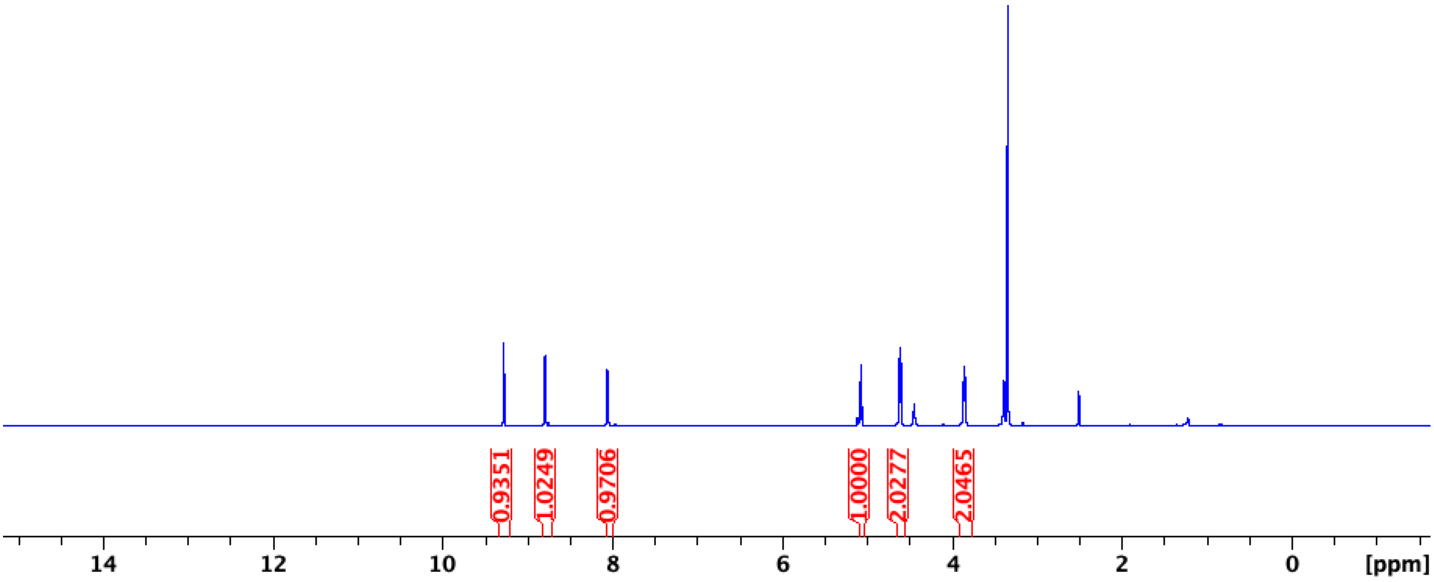

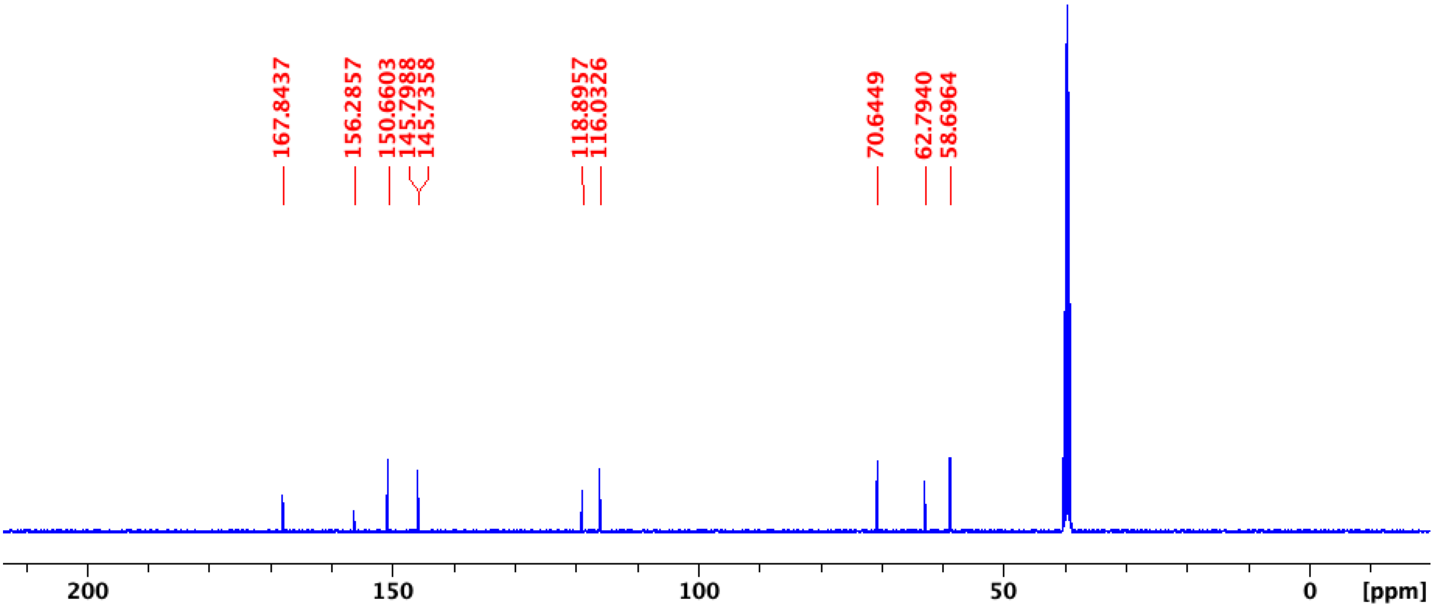

(2*R*)-2-[(2-(2,3-Difluorobenzylmercapto)-pyrido[3,4-*d*]pyrimidine-4-yl)amino]butanol (24a)

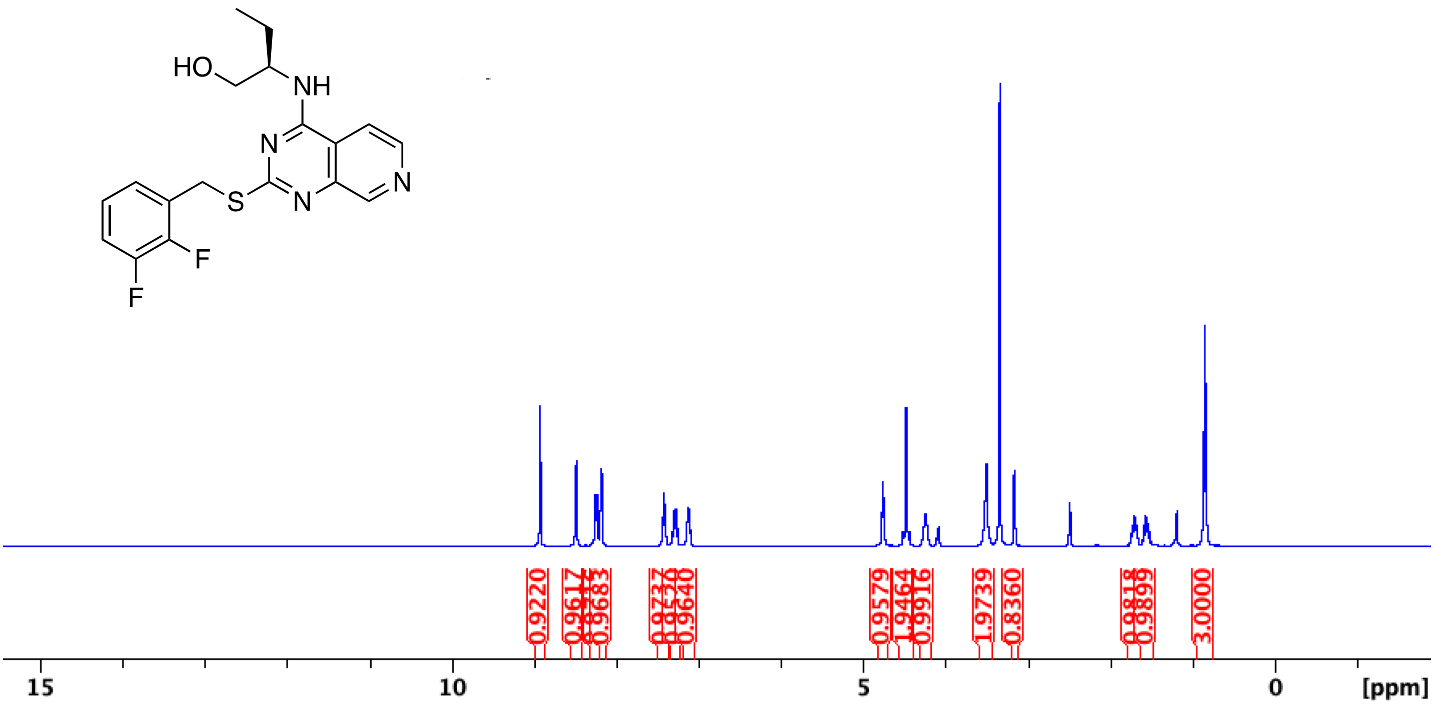

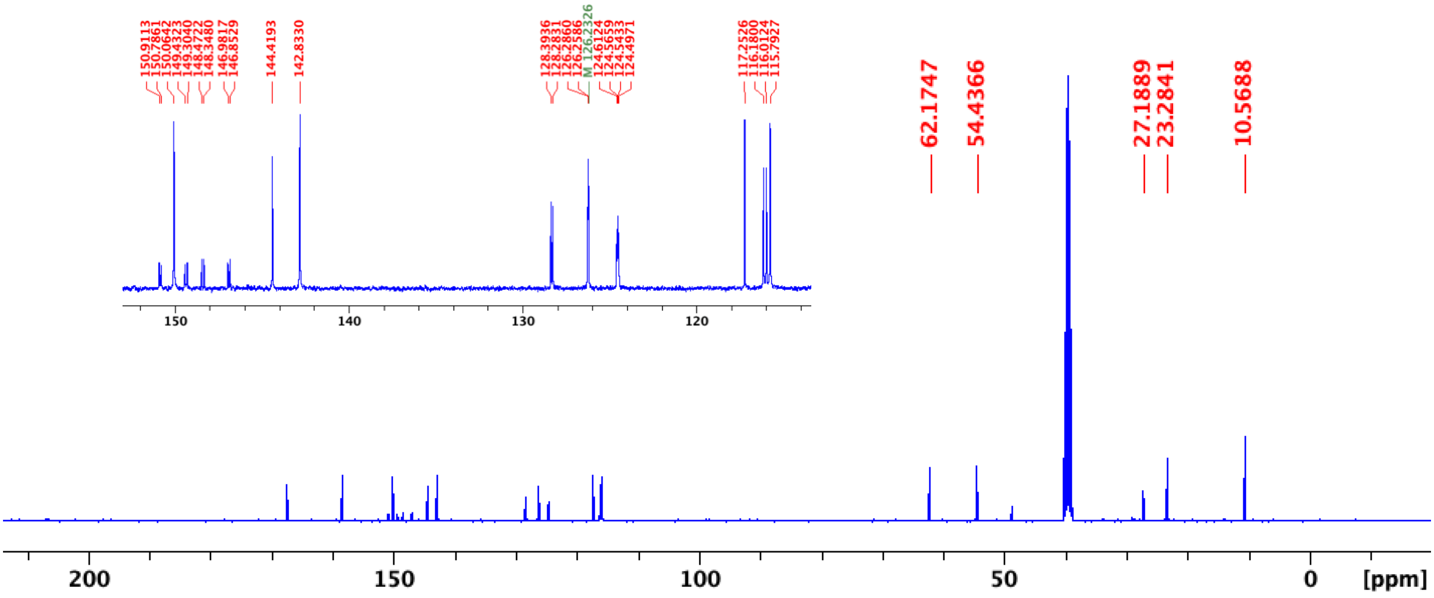

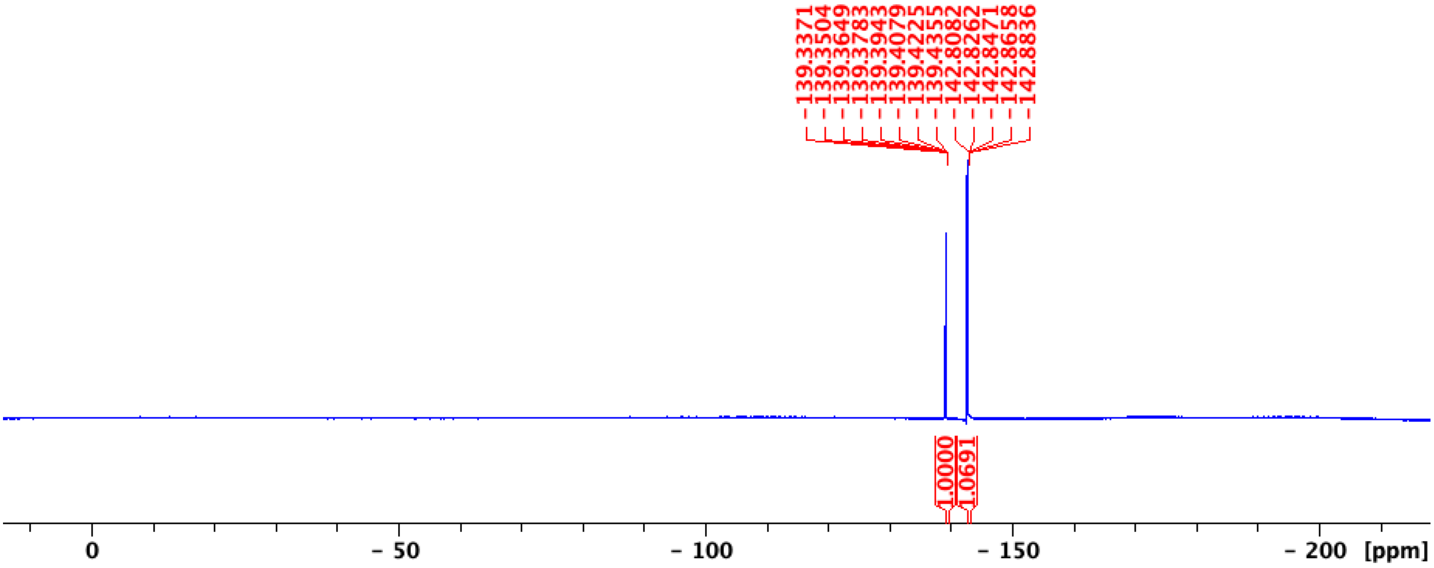

2-[(2-(2,3-Difluorobenzylmercapto)-pyrido[3,4-*d*]pyrimidine-4-yl)amino]-2-methyl-propan-1-ol (24b)

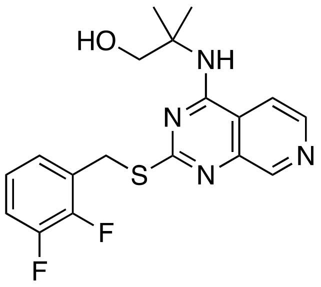

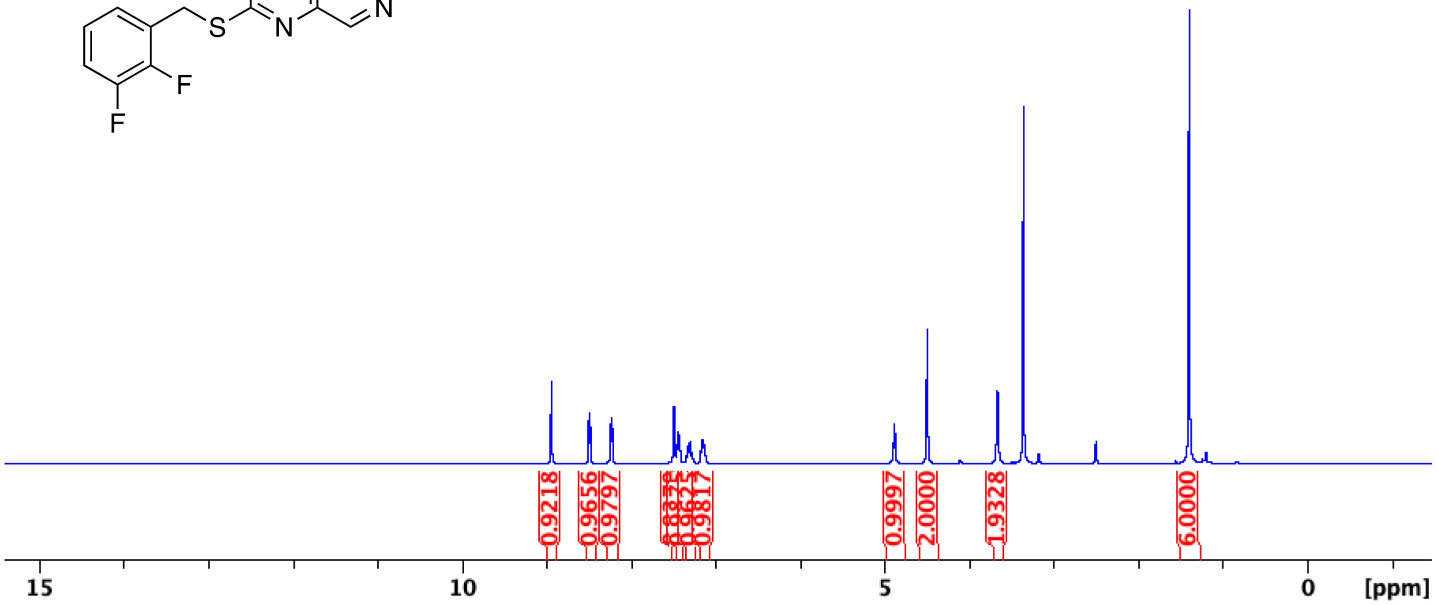

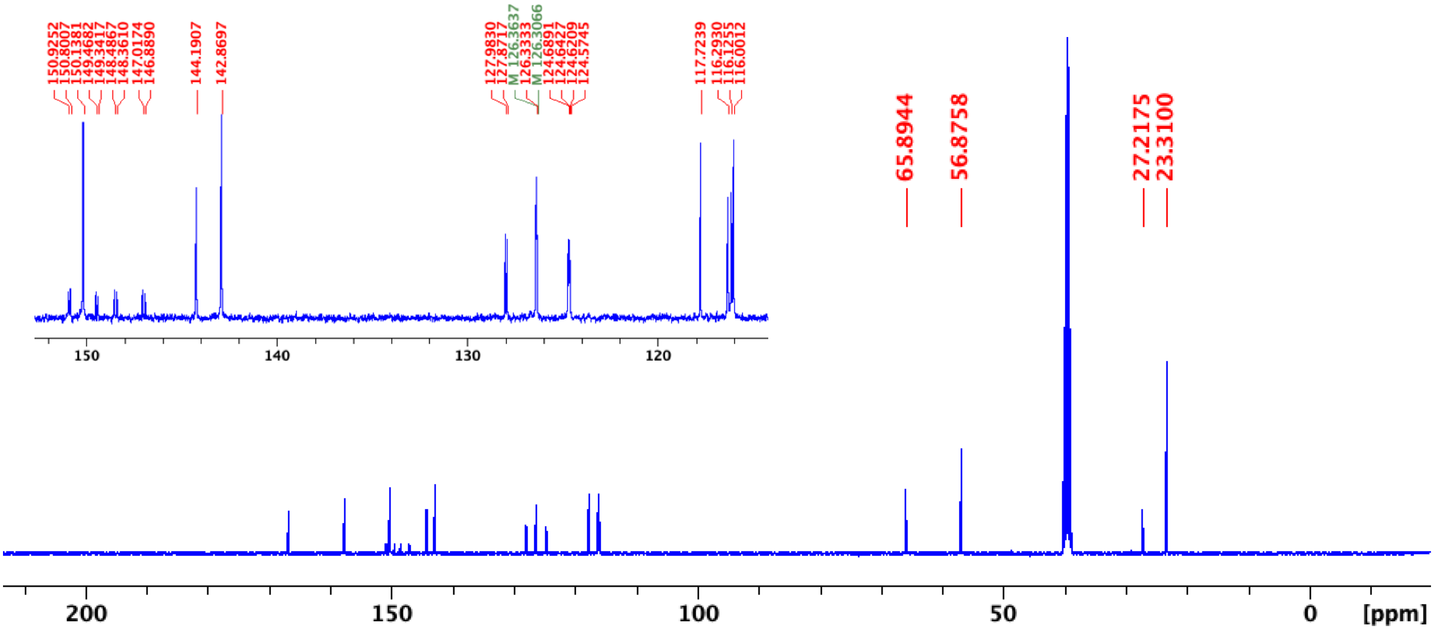
150.9252
150.8007
150.1381
149.4682
149.3417
148.4867
148.3610
147.0174
146.8890
144.1907
142.8697
127.9830
127.8717
M 126.3637
126.3333
M 126.3066
124.6891
124.6427
124.6209
124.5745
117.7239
116.2930
116.1255
116.0012
65.8944
56.8758
27.2175
23.3100
150
140
130
120
200
150
100
50
0
[ppm]

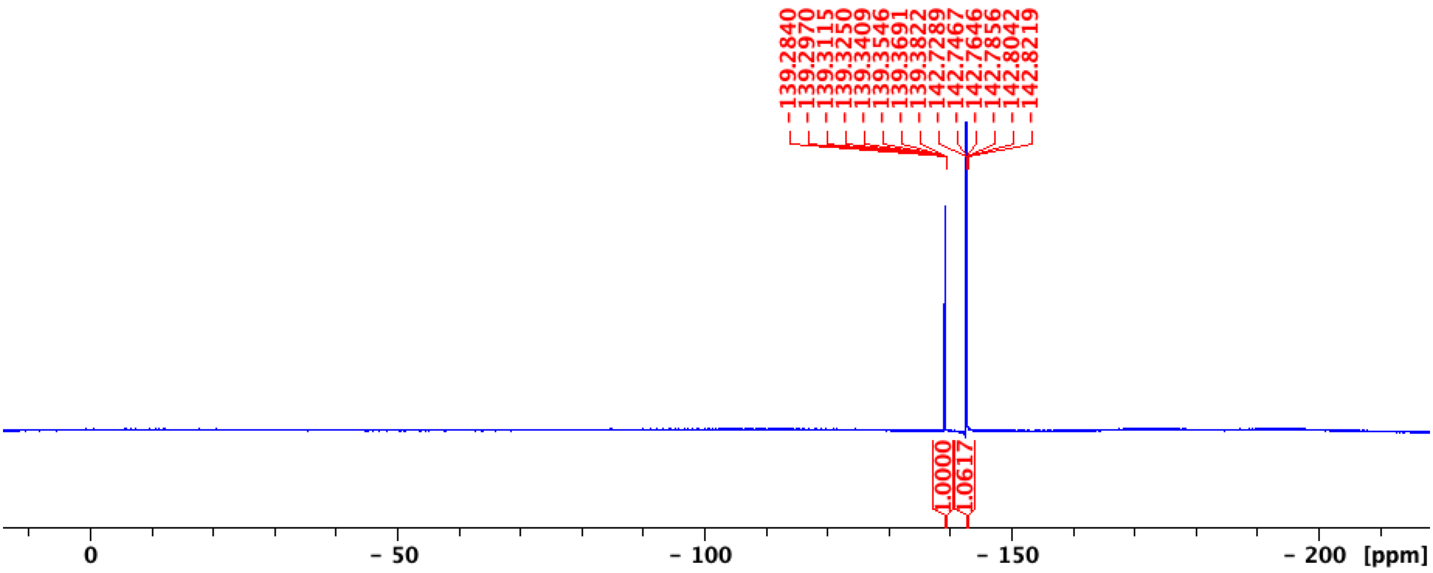
139.2840
139.2970
139.3115
139.3250
139.3409
139.3546
139.3691
139.3822
142.7289
142.7467
142.7646
142.7856
142.8042
142.8219
1.0000
1.0617
0
- 50
- 100
- 150
- 200
[ppm]

3-[(2-(2,3-Difluorobenzylmercapto)-pyrido[3,4-*d*]pyrimidine-4-yl)amino]propan-1,2-diol (24c)

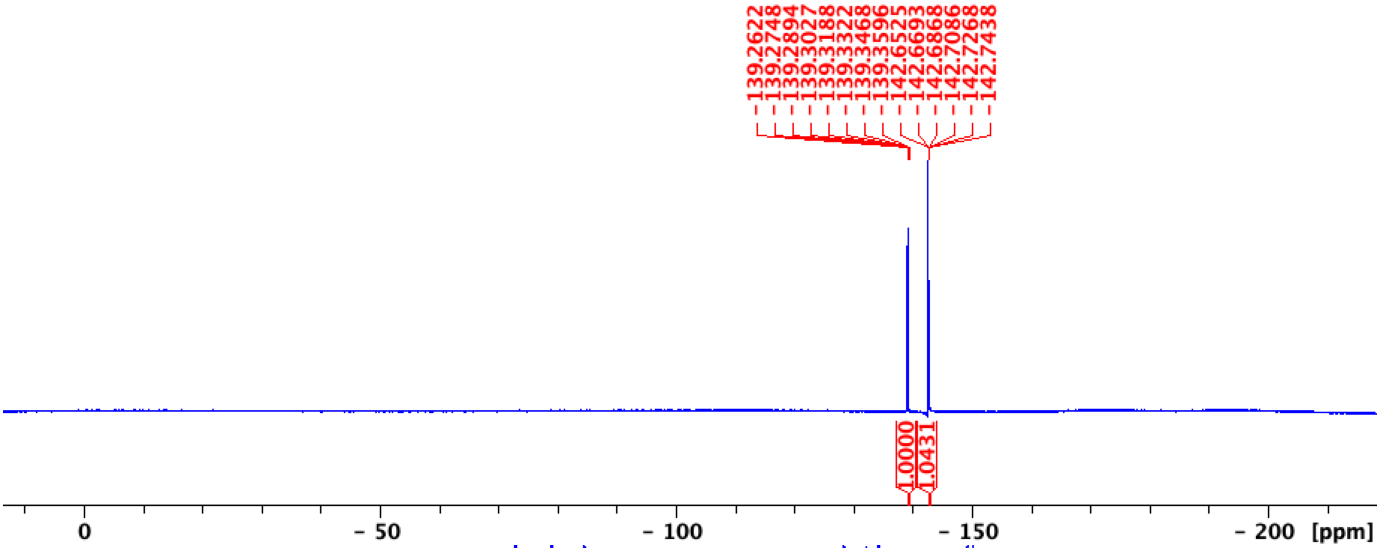

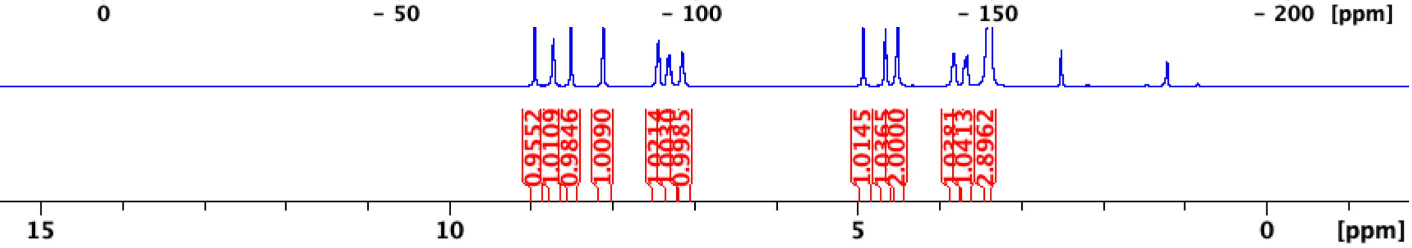

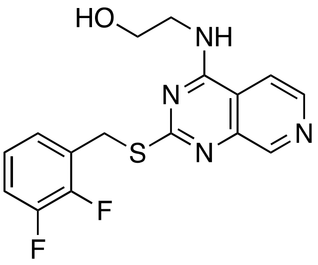

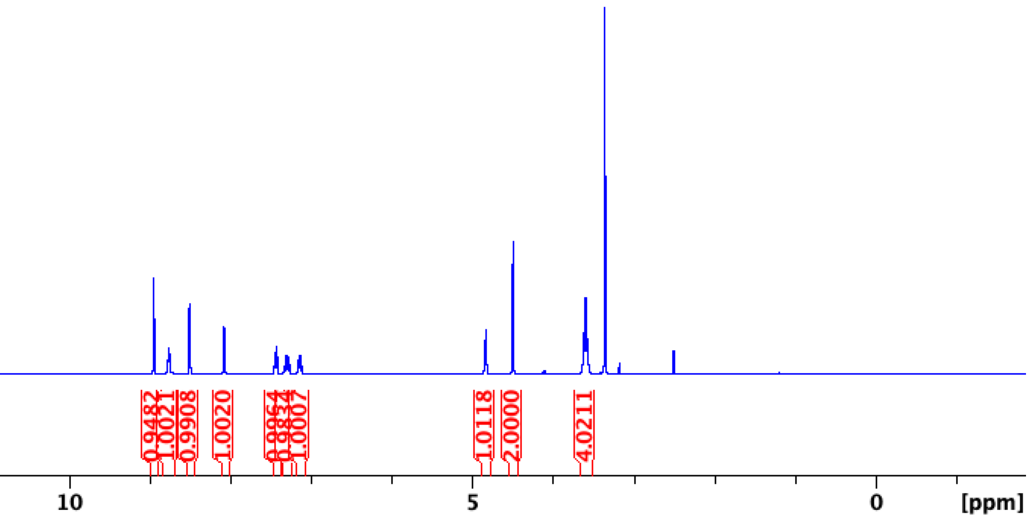

3-

[(2-

(2,3-

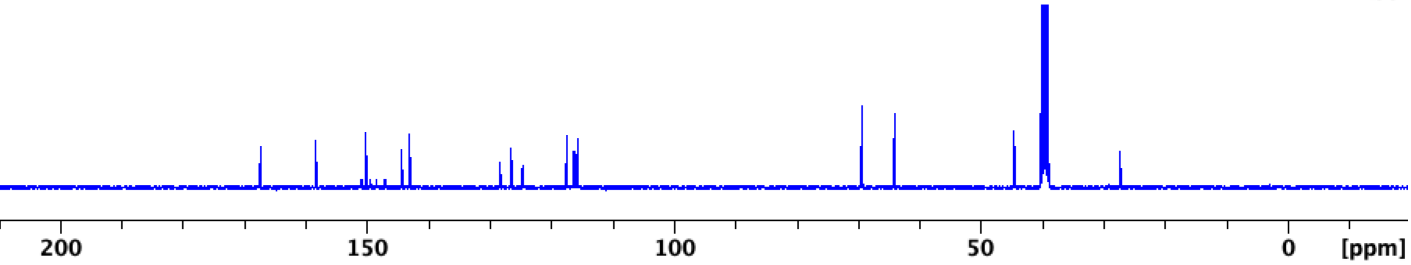

Difluorobenzylmercapto)-pyrido[3,4-*d*]pyrimidine-4-yl)amino]ethanol (24d)

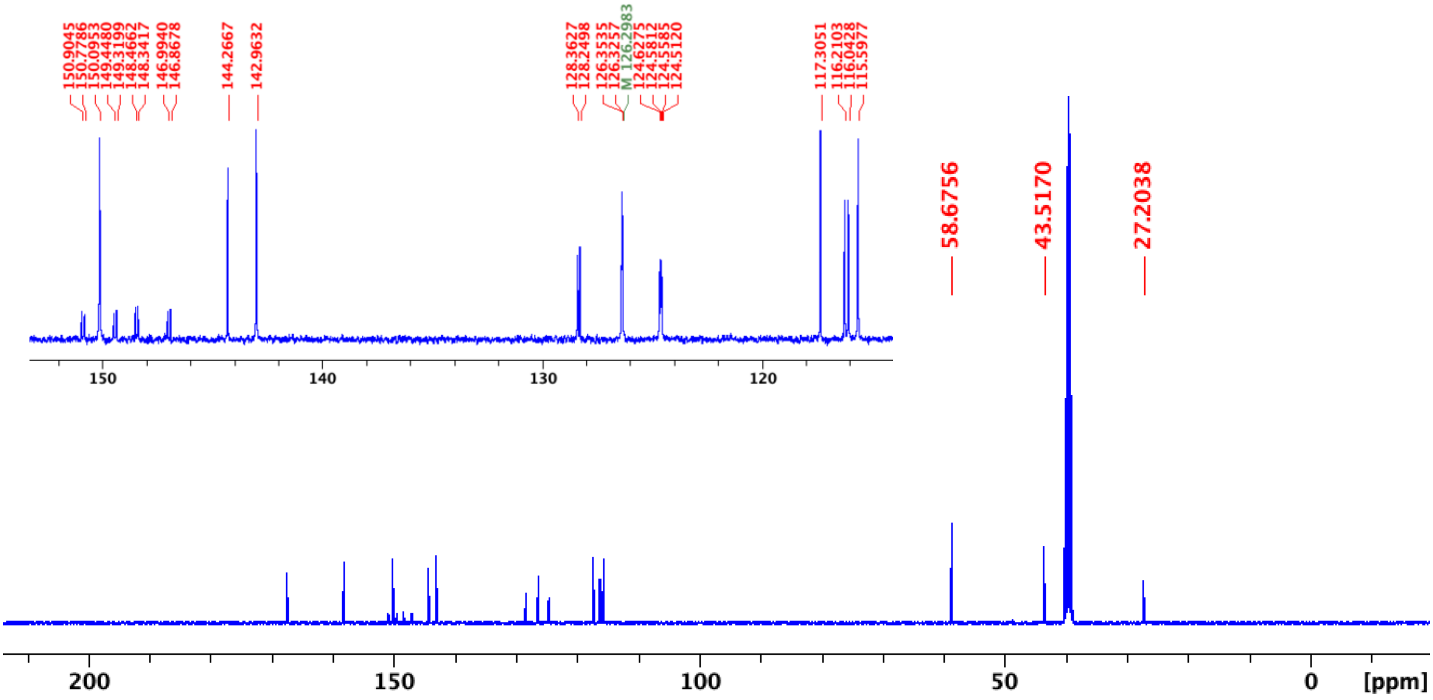
150.9045
150.7786
150.0953
149.4480
149.3199
148.4662
148.3417
146.9940
146.8678
144.2667
142.9632
128.3627
128.2498
126.3535
126.3257
M 126.2983
124.6275
124.5812
124.5585
124.5120
117.3051
116.2103
116.0428
115.5977
58.6756
43.5170
27.2038
150
140
130
120
200
150
100
50
0
[ppm]

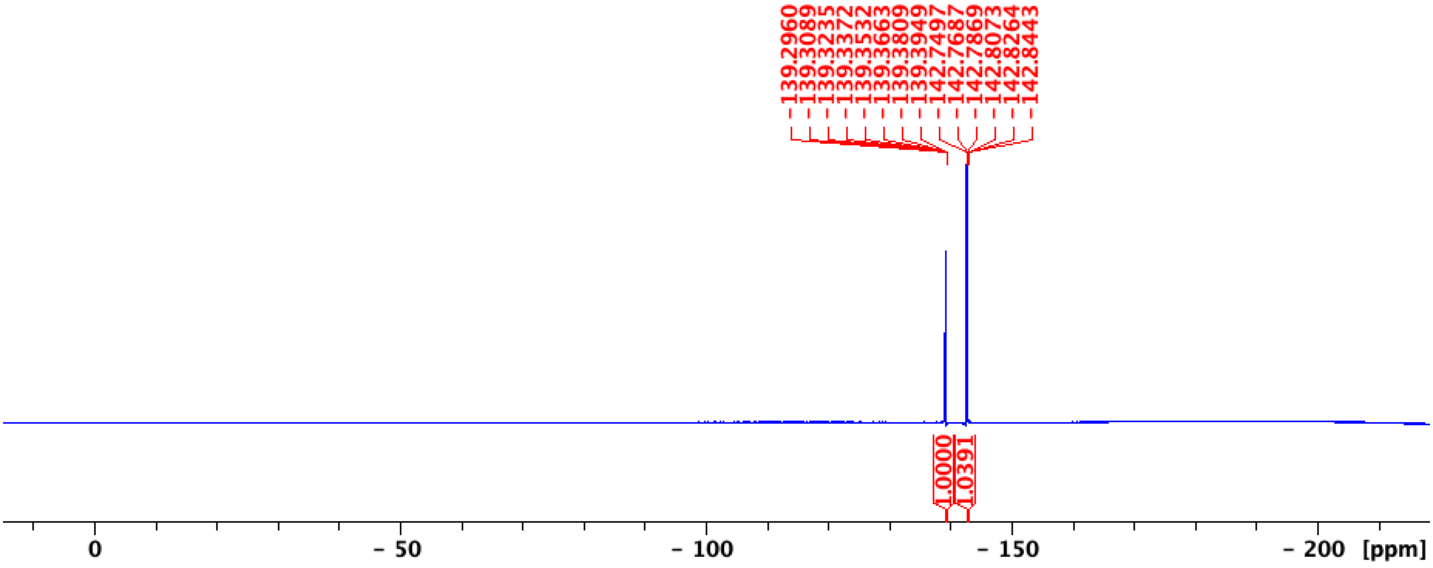
-139.2960
-139.3089
-139.3235
-139.3372
-139.3532
-139.3663
-139.3809
-139.3949
-142.7497
-142.7687
-142.7869
-142.8073
-142.8264
-142.8443
1.0000
1.0391
0
- 50
- 100
- 150
- 200
[ppm]

3-[(2-(2,3-Difluorobenzylmercapto)-pyrido[3,4-*d*]pyrimidine-4-yl)oxy]ethanol (24e)

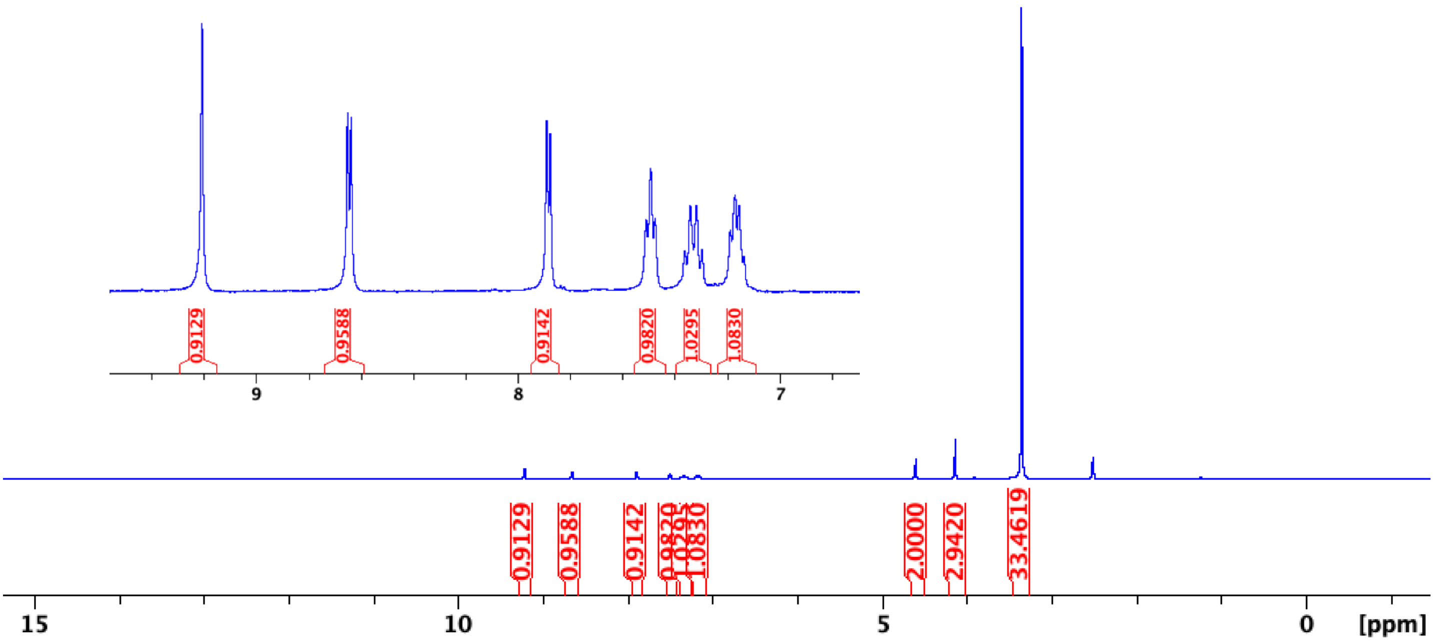

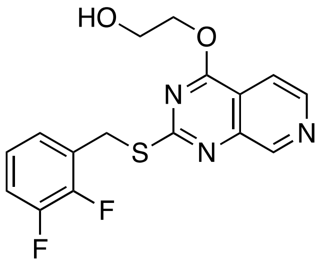

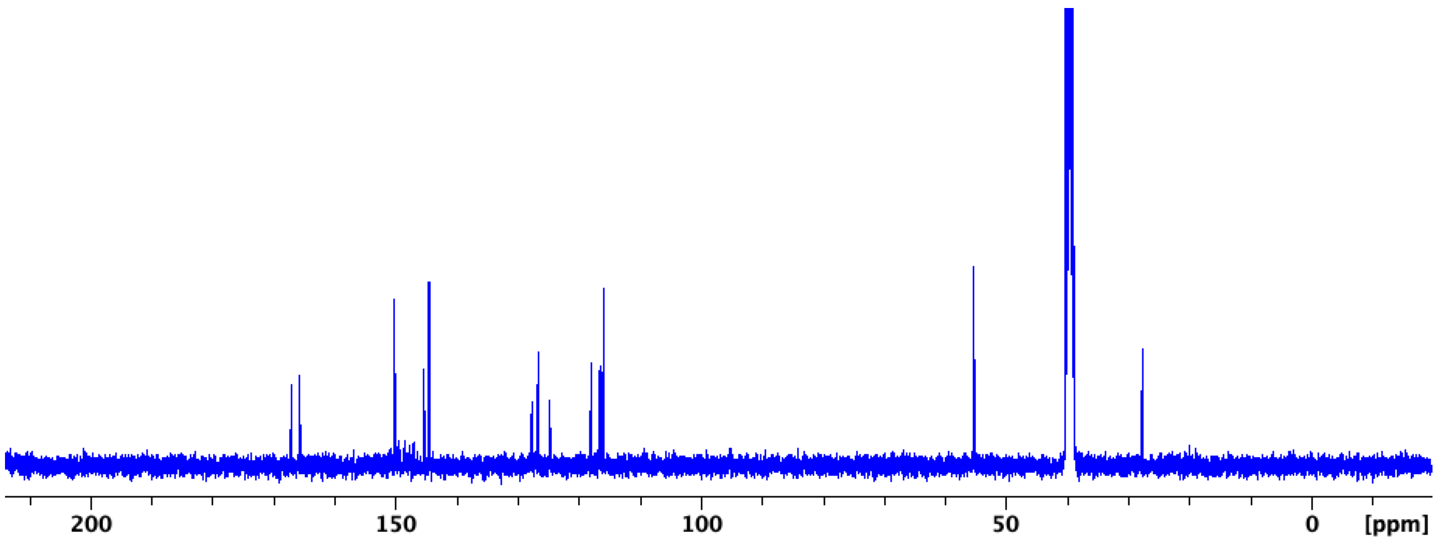
200
150
100
50
0
[ppm]

(2*R*)-2-[(6-Chloro-2-pyrido[3,4-*d*]pyrimidine-4-yl)amino]propanol (25)

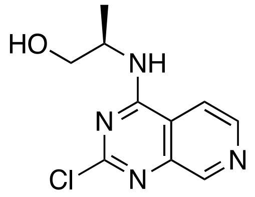

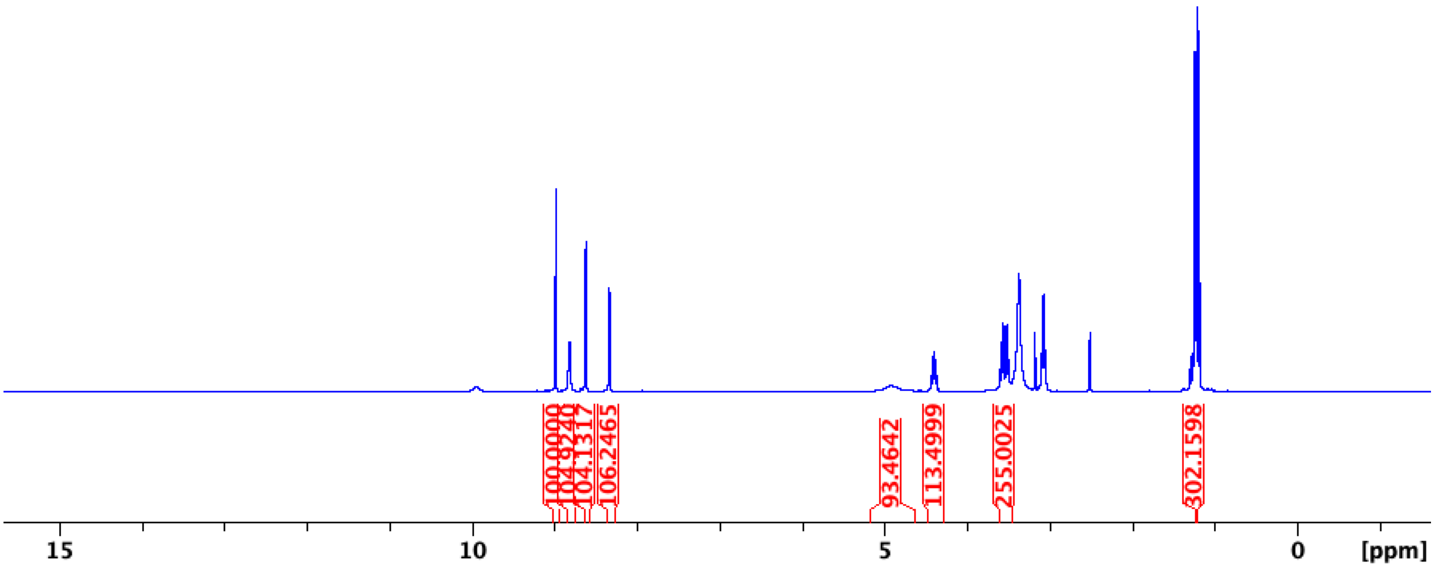

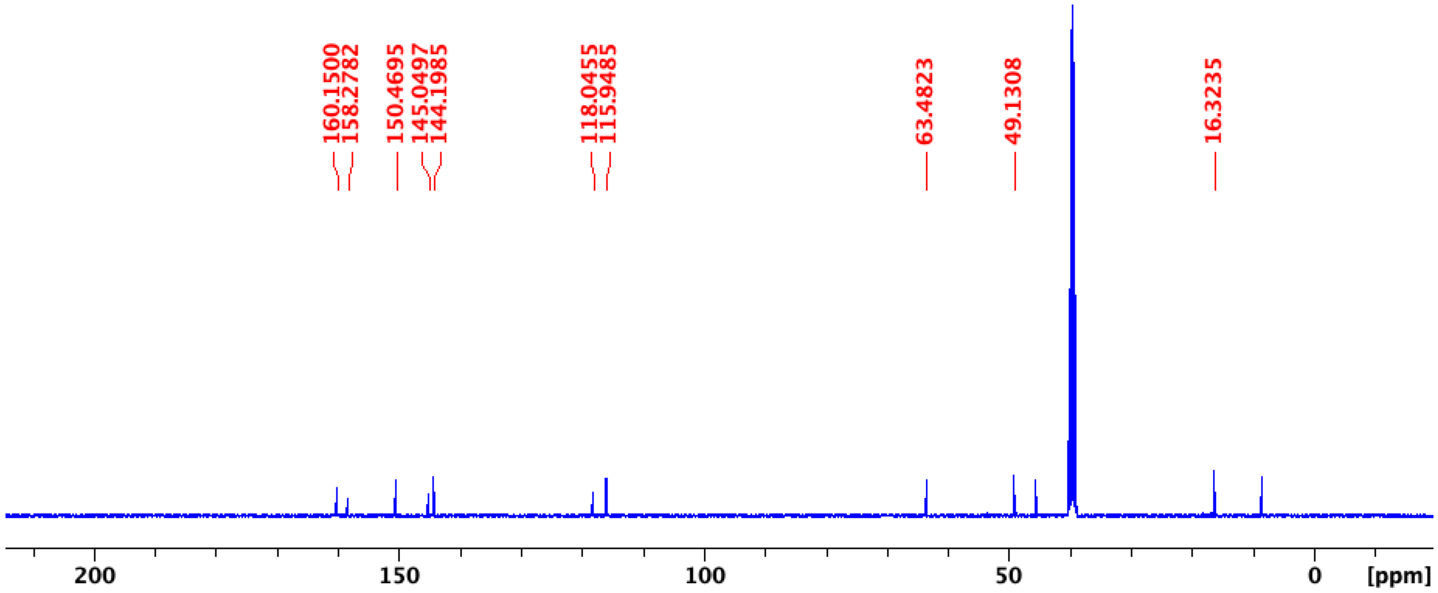

(2*R*)-2-[(2-(2,3-Difluorobenzylamino)-pyrido[3,4-*d*]pyrimidine-4-yl)amino]propanol (26a)

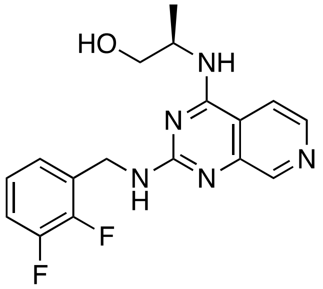

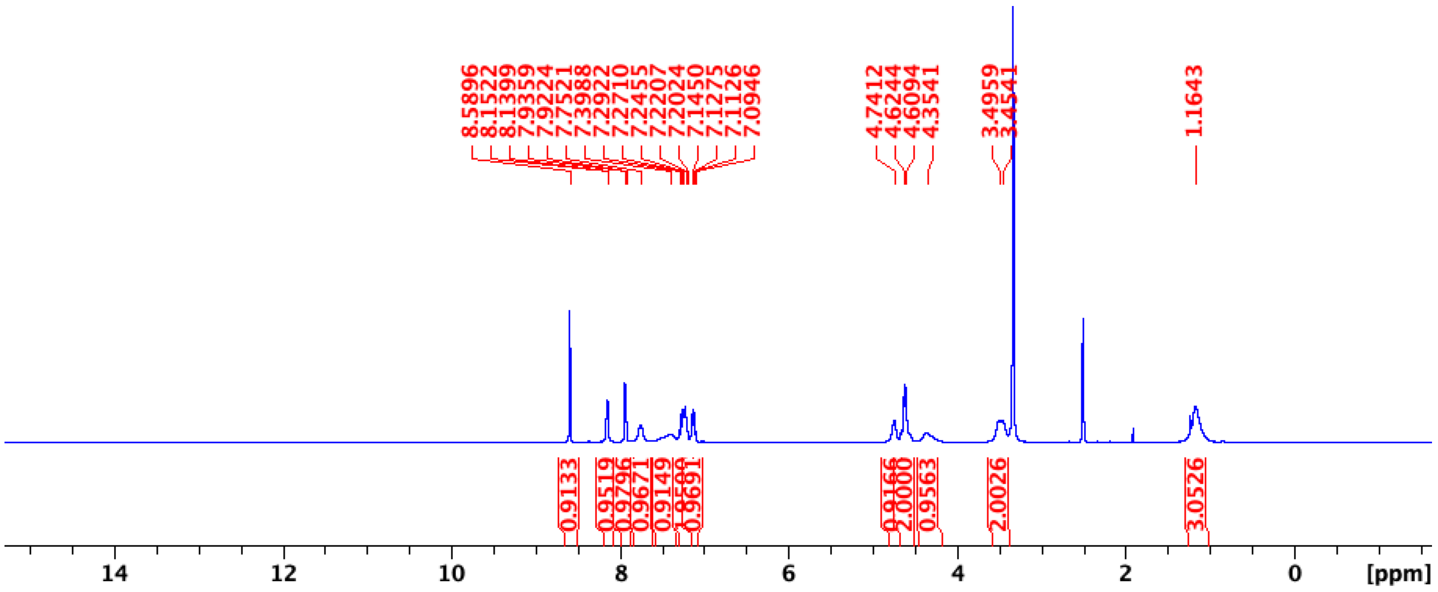

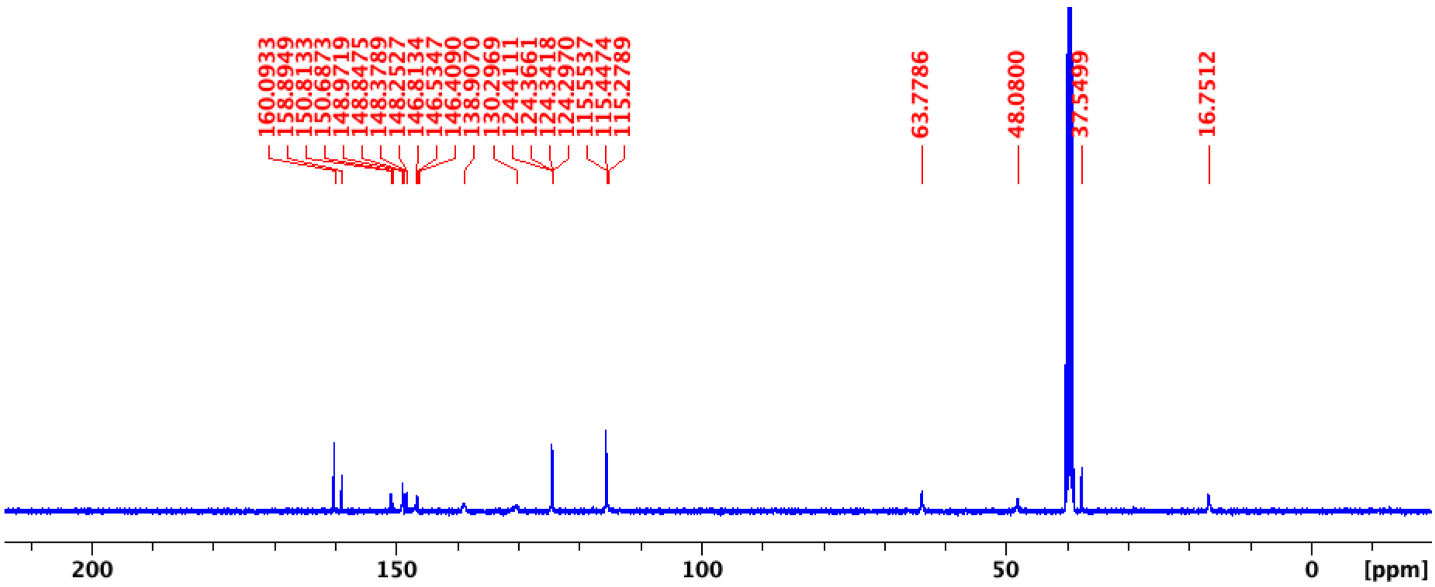

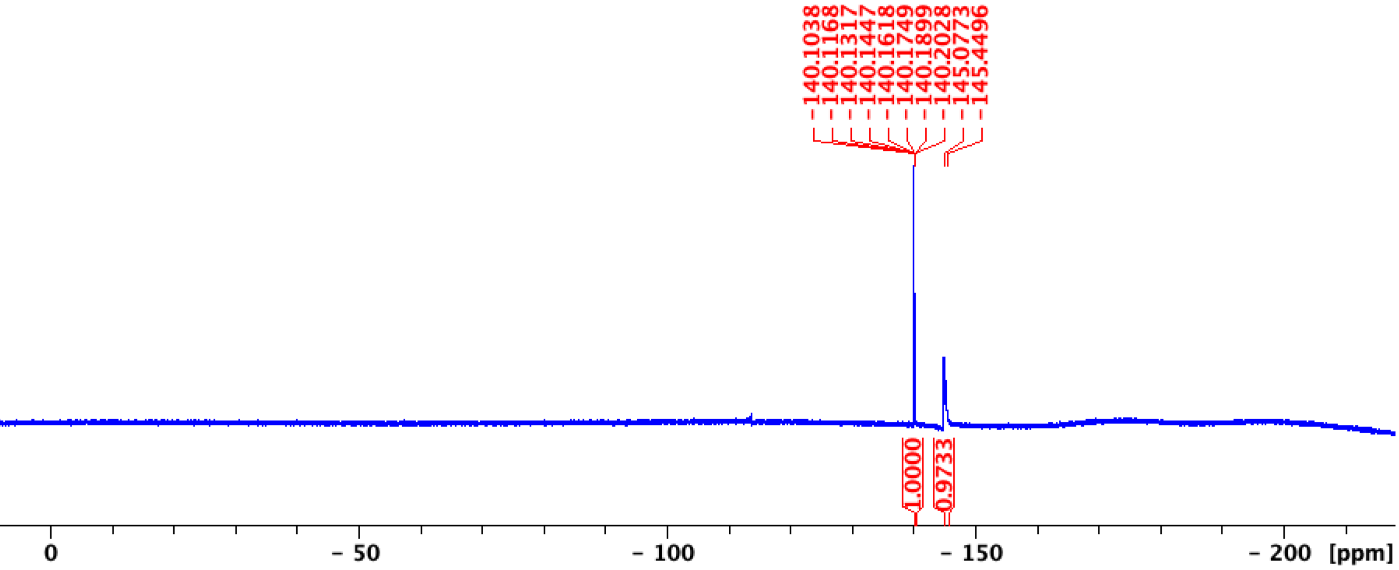

-140.1038
-140.1168
-140.1317
-140.1447
-140.1618
-140.1749
-140.1899
-140.2028
-145.0773
-145.4496
1.0000
0.9733
0
- 50
- 100
- 150
- 200
[ppm]

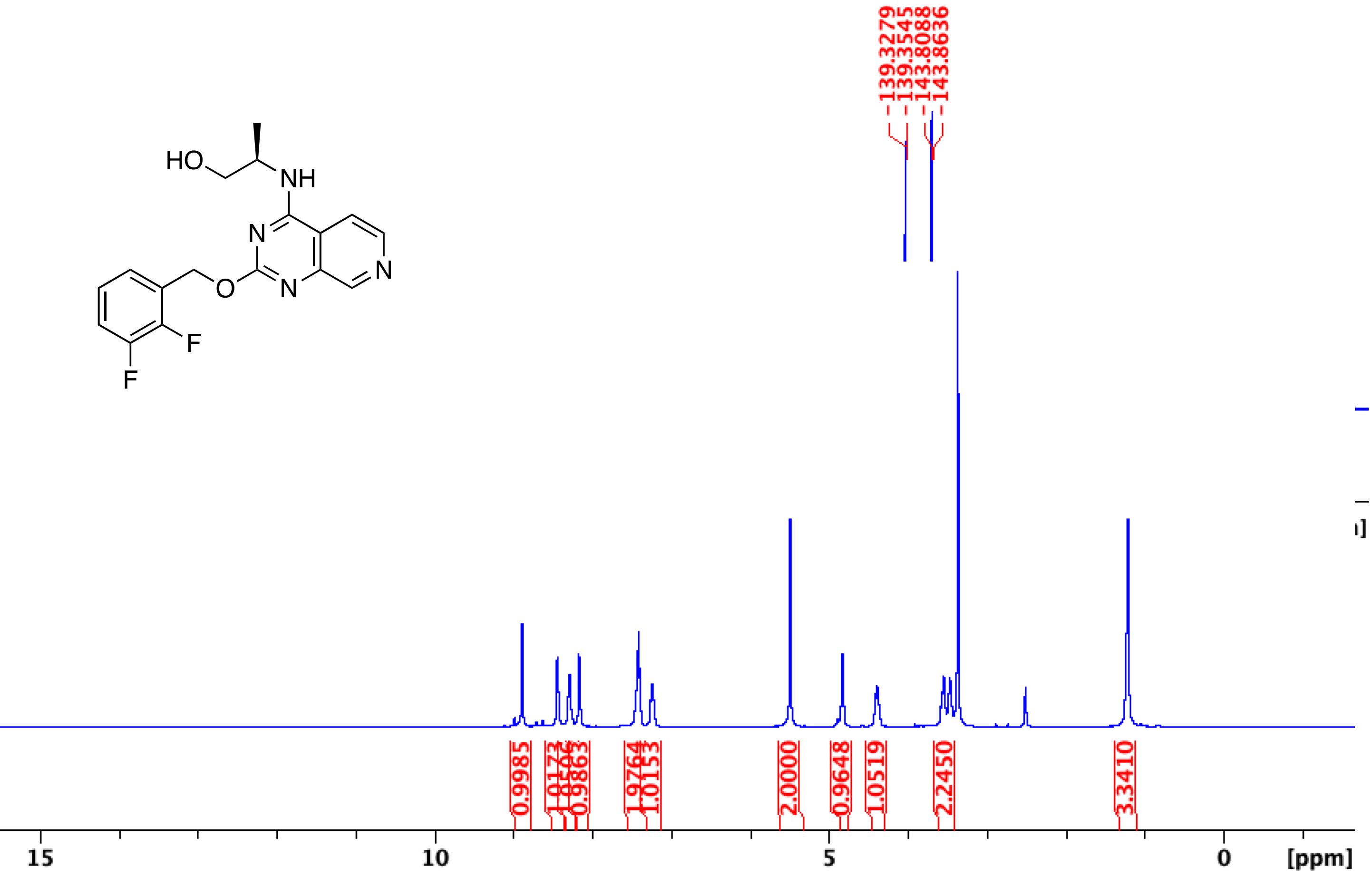

(2*R*)-2-[(2-(2,3-Difluorobenzyloxy)-pyrido[3,4-*d*]pyrimidine-4-yl)amino]propanol (26b)

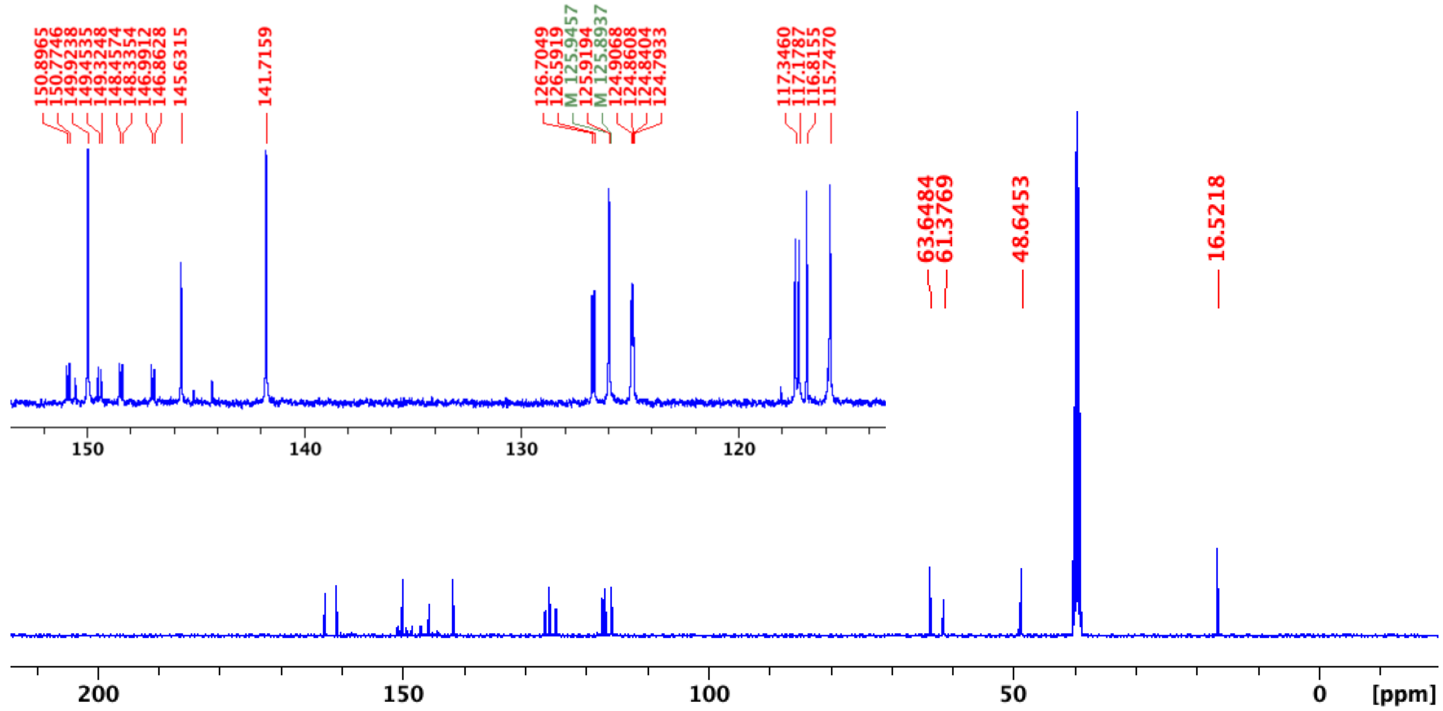

(2*R*)-2-[(2-(Phenylmercapto)-pyrido[3,4-*d*]pyrimidine-4-yl)amino]propanol (26c)

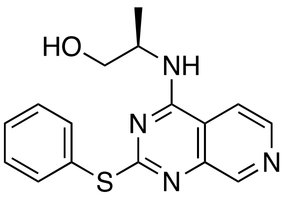

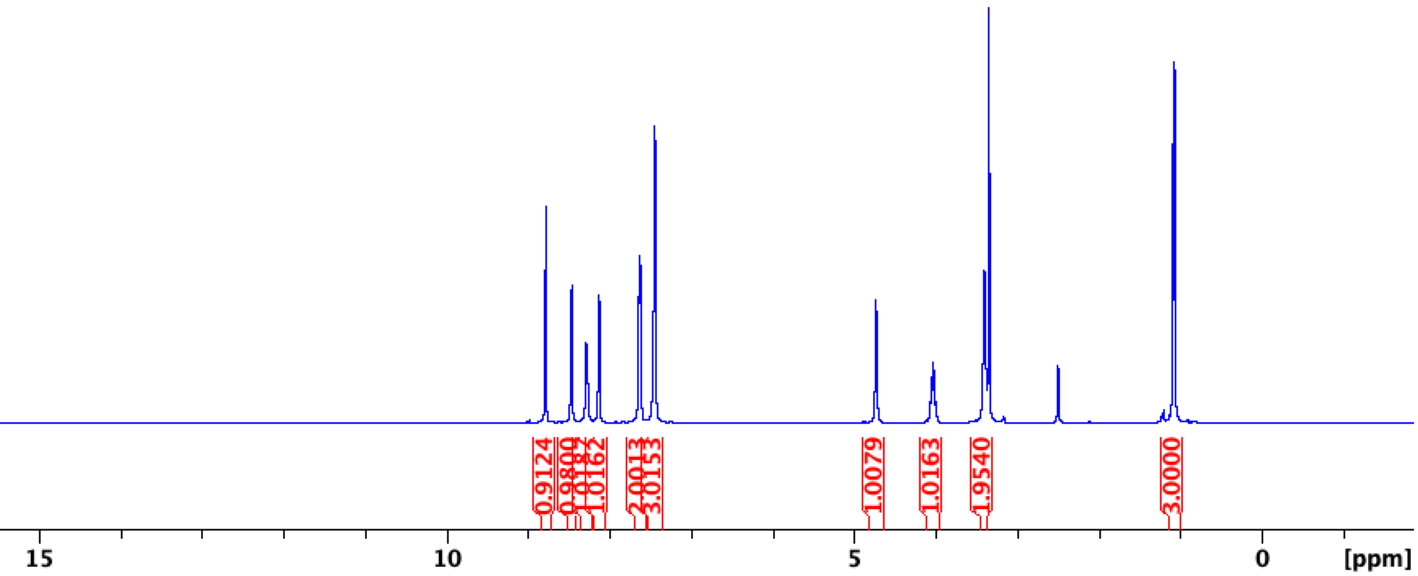

(2*R*)-2-[(2-(Phenylethylmercapto)-pyrido[3,4-*d*]pyrimidine-4-yl)amino]propanol (26d)

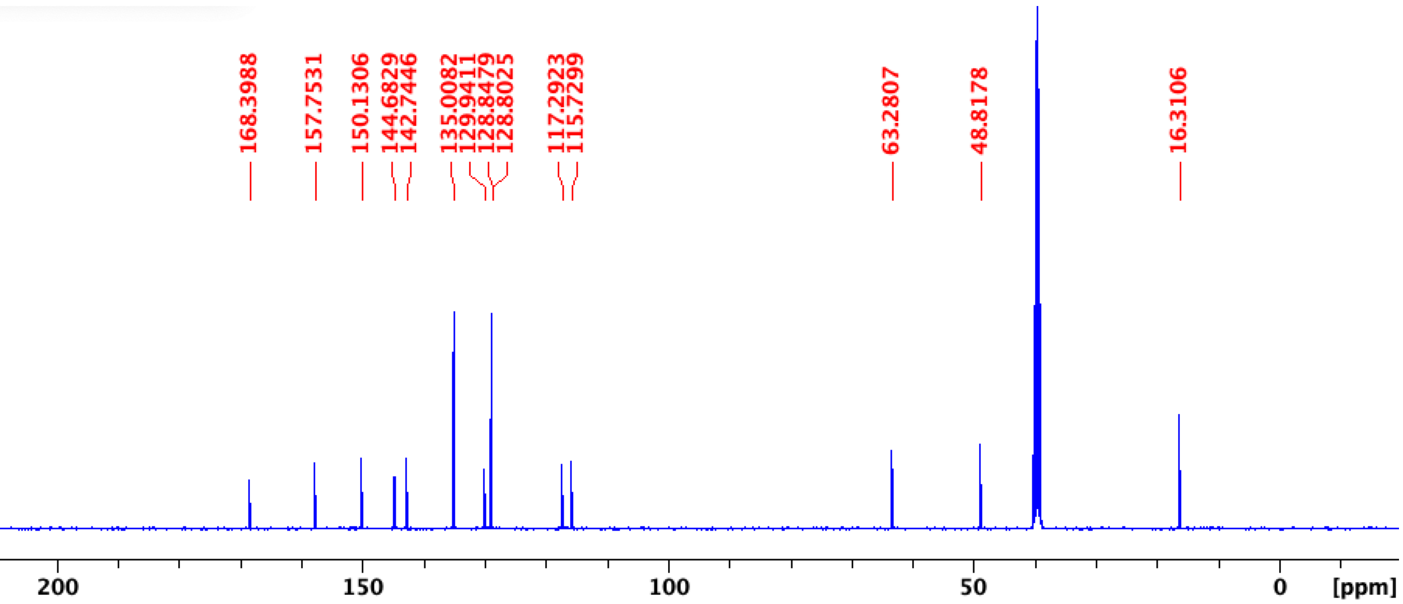

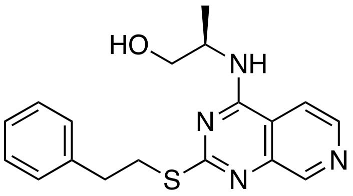

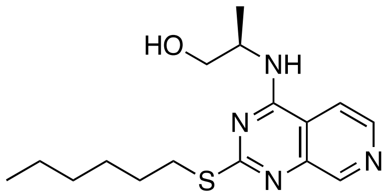

(2*R*)-2-[(2-(Hexylmercapto)-pyrido[3,4-*d*]pyrimidine-4-yl)amino]propanol (26e)

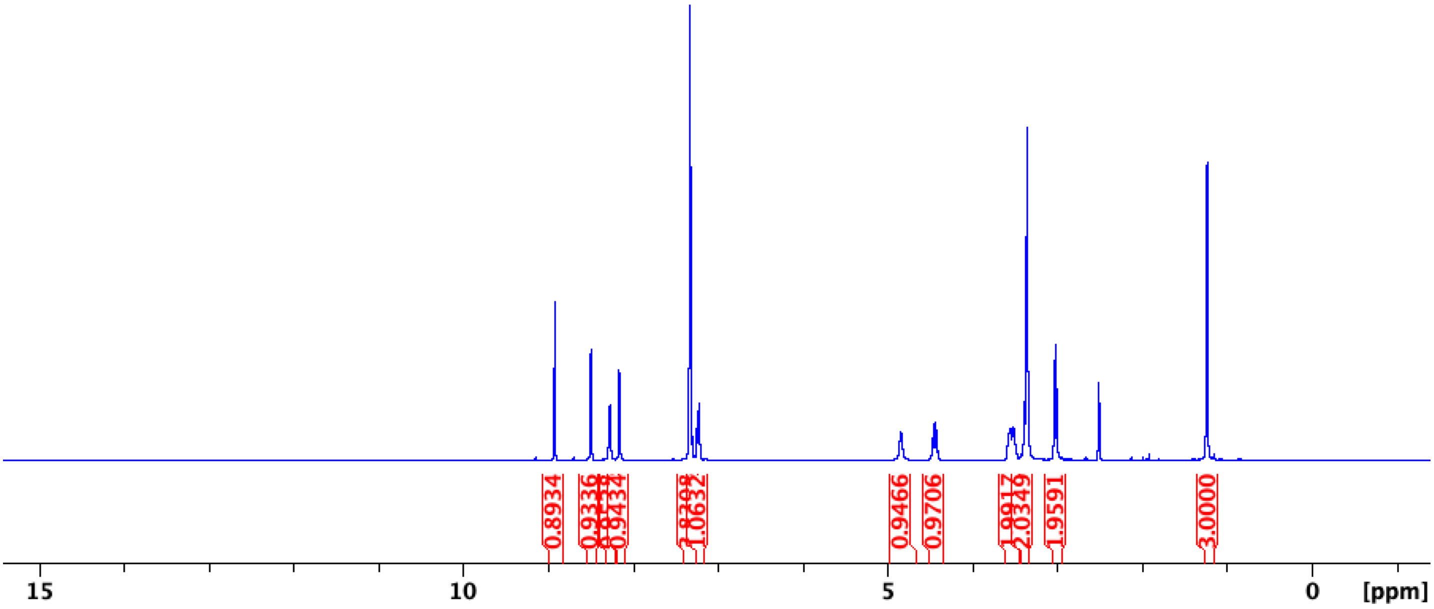

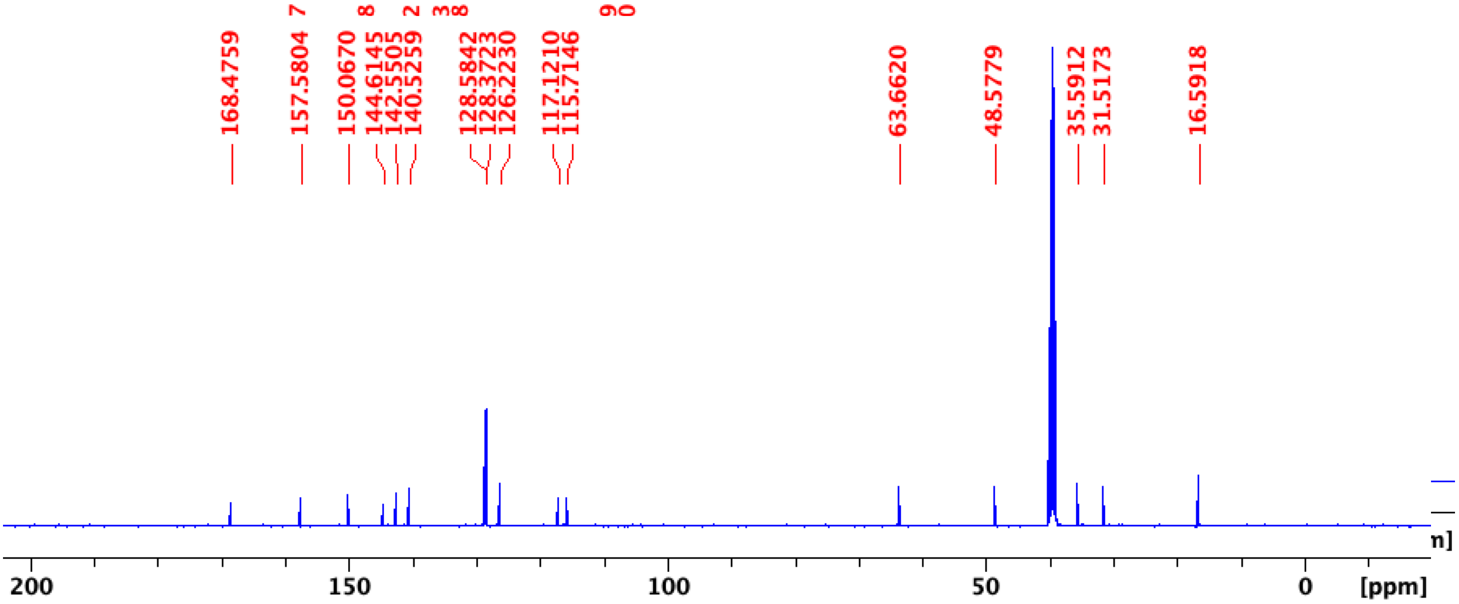

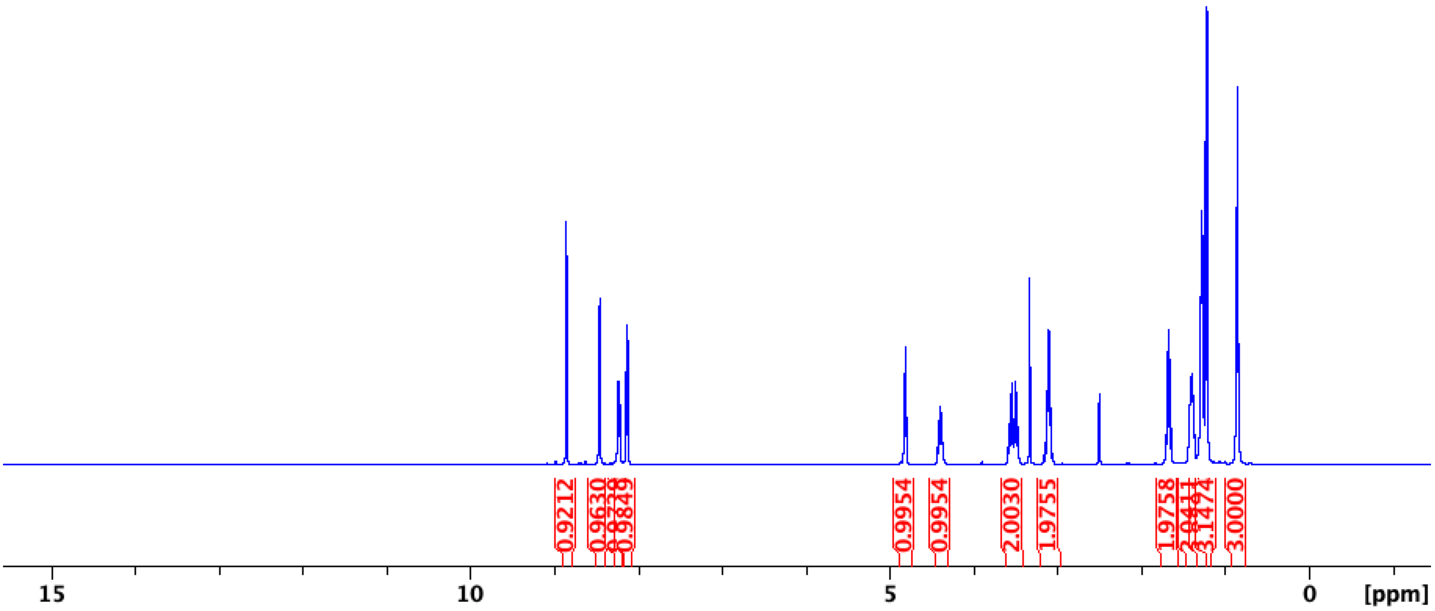
0.9212
0.9630
0.9738
0.9849
0.9954
0.9954
2.0030
1.9755
1.9758
2.0411
3.1474
3.0000
15
10
5
0
[ppm]
